# Placental cytotrophoblast microvillar stabilization is required for cell-cell fusion

**DOI:** 10.1242/dev.204619

**Published:** 2025-04-11

**Authors:** Wendy K. Duan, Sumaiyah Z. Shaha, Juan F. Garcia Rivas, Bethan L. Wilson, Khushali J. Patel, Ivan K. Domingo, Meghan R. Riddell

**Affiliations:** ^1^ Department of Physiology, University of Alberta, Edmonton, Alberta T6G 2S2, Canada; ^2^ Department of Obstetrics and Gynecology, University of Alberta, Edmonton, Alberta T6G 2S2, Canada

**Keywords:** Trophoblast, Cell fusion, Ezrin, Microvilli, Placenta

## Abstract

The placenta is an essential organ of pregnancy required for maternal-fetal transport and communication. The surface of the placenta facing the maternal blood is formed by a single giant multinucleate cell: the syncytiotrophoblast. The syncytiotrophoblast is formed and maintained via fusion of progenitor cytotrophoblasts. Cell-cell fusion is a tightly regulated process, and in non-trophoblastic cells is accompanied by stereotypical alterations in cell shape by cells that have attained fusion-competence. The most prominent feature is the formation of actin-based membrane protrusions, but whether stereotypic morphological changes occur in fusion-competent cytotrophoblasts has not been characterized. Using a human placental explant model and trophoblast organoids, we identify microvilliation as a morphological feature that is enriched prior to fusion of cytotrophoblasts. Disruption of microvilli using an inhibitor of the actin-membrane cross-linker protein ezrin blocked cytotrophoblast fusion in both models. We provide evidence that cytotrophoblast microvilli are enriched in early endosomes and a pro-fusogenic protein. Thus, we propose that the polarized assembly of microvillar domains is crucial for mediating efficient syncytiotrophoblast development.

## Teaser

**Highlighted Article:** Fusion-competent cytotrophoblasts undergo dynamic changes in cell morphology, including the acquisition of apically localized microvilli, the stabilization of which facilitates effective fusion and differentiation.

## INTRODUCTION

The placenta is an embryonically derived organ of pregnancy that carries out crucial functions such as maternal-fetal nutrient exchange, hormone production, and protection from pathogens and maternal leukocytes. Trophoblasts are the placental epithelial cells. In humans, the syncytiotrophoblast (ST), a giant multinucleate single cell, covers nearly the entire maternal blood-facing surface. This embryonically derived cell serves as both a fetal sentinel and nutrient exchanger within the maternal compartment. By the end of gestation, the ST has been estimated to be ∼12 m^2^ and contain billions of nuclei (Simpson et al., 1992). Importantly, the ST is post-mitotic, and it is maintained and expands through the incorporation of its underlying progenitor villous cytotrophoblasts (vCTs) via cell-cell fusion. Adequate formation and renewal of the ST via vCT fusion is crucial for proper placental function. Altered vCT fusion is a feature of the common pregnancy complications intrauterine growth restriction (IUGR) and pre-eclampsia (Gauster et al., 2009; Langbein et al., 2008; Ruebner et al., 2010), which have a shared aetiology of placental malformation. Yet much remains to be understood about the regulation of vCT fusion.

Cell-cell fusion (hereafter referred to as cell fusion) is a relatively uncommon but essential event in biology. It occurs between gametes when the sperm and oocyte fuse, in myoblasts to maintain and form myofibers, and in the fusion between cells of the villous trophoblast lineage. Fusion is highly regulated to ensure it occurs between the right cells at the correct location and time within a tissue, and requires the expression of fusogens, proteins that catalyse the local disruption and hemi-fusion of the lipid bilayers. It has been proposed that fusion can be broken down into three stages: (1) attainment of fusion competence; (2) commitment to fusing; and (3) the fusion event (Aguilar et al., 2013). In myoblasts, these stages have been extensively characterized in both mammalian and *Drosophila* cells (Abmayr and Pavlath, 2012; Kim and Chen, 2019; Petrany and Millay, 2019), but our understanding of the regulation and execution of trophoblast fusion is less developed.

Single cell RNA-seq and single nuclei RNA-seq analyses have revealed that multiple transcriptionally distinct vCT populations exist concurrently in the human placenta (Arutyunyan et al., 2023; Shannon et al., 2022; Wang et al., 2024). These analyses have identified a trophoblastic subpopulation assumed to be fusion-competent due to the high expression of ST markers such as placental fusogen syncytin 2 (*ERVFRD-1*) (Arutyunyan et al., 2023; Keenen et al., 2024; Shannon et al., 2022; Wang et al., 2024). Similarly, ultrastructural examination of first trimester and term placenta has revealed a vCT subpopulation that more strongly resemble the ST in their nuclear structure and organellular composition (Jones and Fox, 1991). It is well established that fusion commitment is accompanied by polarization at the site of fusion. For example, myoblasts are known to change their cell shape to laterally align with the myofiber with which they will fuse (Lehka and Redowicz, 2020), and the appearance of membrane protrusions on fusion competent cells is also a highly conserved feature among fusogenic cell types (Brukman et al., 2019; Wilson and Snell, 1998). These shape changes appear to have the purpose of bringing fusing membranes into closer approximation (Brukman et al., 2019; Kim and Chen, 2019; Wilson and Snell, 1998), and alterations in cell surface area immediately post-fusion have been proposed to regulate transcriptional commitment to a differentiated state via an AMPK-YAP1 signalling pathway (Feliciano et al., 2021). Bundled filamentous-actin (F-actin) is a core cytoskeletal component of membrane protrusions observed in myoblasts where the formation of invasive membrane podosomes allows for efficient fusogen engagement and mechanical coordination between the membranes of apposing cells (Kim and Chen, 2019; Kim et al., 2015; Petrany and Millay, 2019; Sens et al., 2010; Shilagardi et al., 2013). Membrane projections have been observed by brightfield live cell imaging between fusing BeWo choriocarcinoma cells (Wang et al., 2014). The variable presence of membrane protrusions in primary vCTs undergoing spontaneous fusion has been observed by scanning electron microscopy (SEM) (Bax et al., 1989), but the cytoskeletal components driving these protrusions and characterization of other shape factors that may distinguish fusion-competent vCTs have not been identified.

Here, we have characterized cellular shape parameters of the vCT layer in human first trimester placental tissue. To identify the shape characteristics that are associated with fusion commitment, we exploited a first trimester explant model, where widespread spontaneous vCT fusion occurs after the removal of the overlying ST layer (Baczyk et al., 2009, 2013; Miller et al., 2005). We identified that the population of vCTs stimulated to fuse by ST denudation are enriched in cells that acquire polarization and accumulate surface area in the apical region via microvilli. We provide evidence that vCT microvilli serve to accumulate fusion-promoting proteins and polarize endocytic trafficking. By inactivating the key microvillar scaffolding protein ezrin, we discovered that vCT fusion requires the formation or stabilization of microvillar membrane projections.

## RESULTS

### A ST regeneration explant model recapitulates syncytialization *ex vivo*

**Fig. 1.**
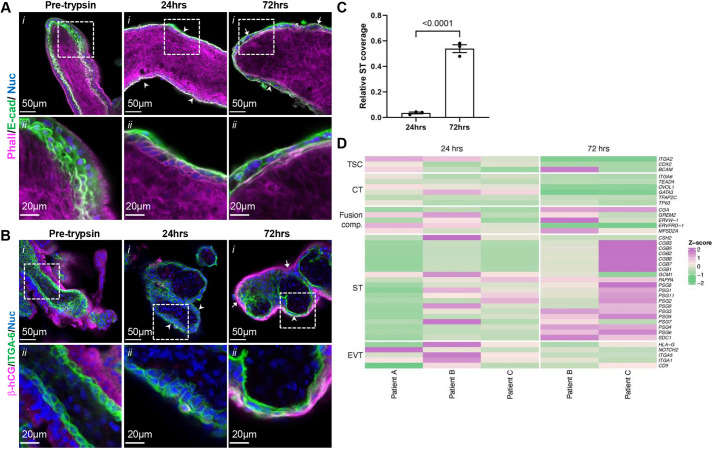
**The syncytiotrophoblast regeneration model synchronizes syncytiotrophoblast development.** (A) Representative *xy*-plane confocal microscopy images of 12 weeks gestational age (GA) placental tissue in pre-trypsinized controls, at 24 h post-trypsinization and at 72 h post-trypsinization. (i) Merged images of phalloidin (magenta), anti-E-cadherin (green) and Hoechst (nuclei; blue). (ii) Higher magnification image of indicated region in i. (B) Representative *xy*-plane confocal microscopy images of 9.5 weeks GA placental tissue in pre-trypsinized controls at 24 h post-trypsinization and 72 h post-trypsinization. (i) Merged images of anti-β-hCG (magenta), anti-ITGA6 (green) and Hoechst (nuclei; blue). (ii) Higher magnification images of indicated regions in i. Scale bars: 50 µm in i; 20 µm in ii. Arrows indicate ST regions; arrowheads indicate villous cytotrophoblast (vCT) regions. (C) Summary data of syncytiotrophoblast (ST) coverage (ST:vCT) at 24 h and 72 h normalized to pre-trypsin controls. Data are mean±s.e.m., unpaired parametric Student's *t*-test, *n*=3. ST coverage (ST:vCT) was calculated using Eqn 2 (se Materials and Methods). (D) Heatmap of explants at 24 h and 72 h post-trypsinization based on z-score of trophoblast subpopulation marker genes. Images are representative of three placentas.

To enrich a fusion-competent vCT subpopulation and examine vCT morphological alterations during the fusion process, we established a first trimester human placental explant model. Tissue was briefly trypsinized to promote ST denudation and then cultured to allow for ST regeneration. This model allows observation of 3D spontaneous syncytialization from pre-existing vCT subpopulations on an intact basement membrane. Removal of the pre-existing ST was achieved by 24 h of culture after trypsinization (Fig. 1A,B), exposing mononucleate anti-E-cadherin- and anti-integrin-α-6 (ITGA6)-positive vCTs (Aghababaei et al., 2015; Aplin, 1993; Longtine et al., 2012). By 72 h post-trypsinization, a multinucleate structure displaying strong anti-β-human chorionic gonadotropin (β-hCG) signal (a ST marker) atop or interspersed with mononucleate vCTs was observed (Fig. 1A,B), resulting in a significant increase in both donor-normalized (Fig. 1C) and non-normalized ST coverage (Fig. S1A). Formation of multinucleate structures often led to the appearance of discrete regional exhaustion of the vCT layer within a villus, resulting in areas without a continuous underlying vCT layer but instead containing seemingly isolated vCTs interspersed within or between regenerated ST. ST denudation was found to be very complete and reproducible, but the degree of ST regeneration post-denudation varied between donors (Fig. S1A).

We performed bulk RNA-seq analysis on explant cultured tissues to confirm the ST regeneration observed via imaging at the transcriptional level. Principle component analyses (PCA) on donor-matched explants at 24 h and 72 h post-trypsinization revealed close clustering of the samples by time in culture (Fig. S1B). After removal of outliers, differential gene expression analyses (DEG) revealed a relative increase in ST marker transcripts (*CGB1*, *CGB2*, *PSG3*, *PSG4*, *PSG5* and *SDC1*) and a decrease in trophoblast stem cell [TSC (*ITGA2* and *CDX2*)] and vCT marker transcripts (*ITGA6*, *TEAD4*, *GATA3*, *TFAP2C* and *TP63*) (Shannon et al., 2022, 2024) at 72 h in culture (Fig. 1D). Relative expression of *ERVW-1*, *CGA*, *GREM2* and *MFSD2A*, transcripts that have been shown to have enriched expression within fusion-competent vCT subclusters (Keenen et al., 2024; Shannon et al., 2022, 2024), had higher relative expression after 72 h in culture (Fig. 1D). Markers for non-villous trophoblasts [extravillous trophoblasts (EVT)] were relatively decreased at 72 h. Thus, the transcriptomic profiling supports our imaging analyses indicating that the ST is regenerated in our model.

Single cell RNA-seq analyses have revealed that of the multiple CT subpopulations in the human placenta, ST precursor vCTs (another label used for fusion competent vCTs) have limited expression of proliferative marker genes compared to progenitor CT subpopulations (Shannon et al., 2022). EdU incorporation assays revealed that explant vCT proliferation was significantly decreased at 48 h and 72 h compared to 24 h (Fig. S1C,D), and that highly variable amounts of EdU positive vCTs were present 24 h after trypsinization. All these data indicate that this model reproducibly allows the *de novo* formation of the ST and contains a mixed population of vCT subtypes.

### ST denudation leads to enrichment of a morphologically distinct and polarized vCT subpopulation

**Fig. 2.**
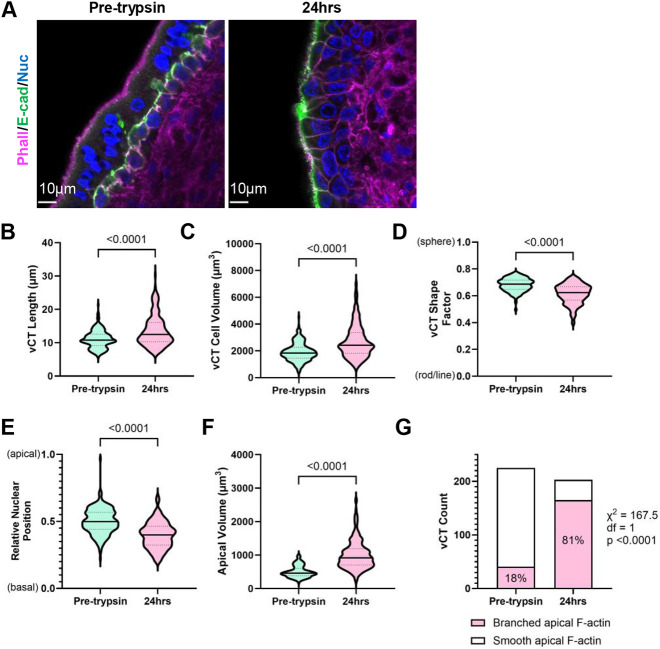
**The syncytiotrophoblast regeneration model enriches a morphologically distinct villous cytotrophoblast subpopulation.** (A) Representative *xy*-plane confocal microscopy images of 12 weeks gestational age (GA) pre-trypsinized control tissue and tissue 24 h post-trypsinization stained with phalloidin (magenta), anti-E-cadherin (green) and Hoechst (nuclei; blue). Scale bars: 10 µm. (B) Summary data of villous cytotrophoblast (vCT) cell length (µm). *n_pretrypsin_*=210 cells; *n_24hrs_*=178 cells. (C) vCT cell volume (µm^3^). *n_pretrypsin_*=237 cells; *n_24hrs_*=290 cells. (D) vCT shape factor. *n_pretrypsin_*=237 cells; *n_24hrs_*=290 cells. (E) Relative position of the nucleus along the apical-basal axis was calculated using Eqn 1 (Materials and Methods). *n_pretrypsin_*=210 cells; *n_24hrs_*=178 cells. (F) vCT apical volume (µm^3^). *n_pretrypsin_*=195 cells; *n_24hrs_*=220 cells. (B-F) Unpaired Mann–Whitney test; solid line indicates the median; dashed lines indicate quartile 1 and quartile 3. (G) Summary counts of vCTs with smooth and branched apical F-actin signal. χ-squared test with Yate's correction. *n_pretrypsin_*=225 cells; *n_24hrs_*=203 cells. Images are representative of three placentas.

Since the removal of the ST layer led to spontaneous ST regeneration and an apparent diminishment of the vCT pool, we surmised there was an enrichment in fusion-competent vCTs in ST-denuded compared to ST-intact tissue, allowing the identification of their characteristic morphology. We acquired high-magnification confocal images of uncultured placental tissue and explants at 24 h post-trypsinization (Fig. 2A) to quantify population-wide shifts in morphological parameters observed with ST denudation. ST-denuded explants were enriched with hypertrophic vCTs, resulting in upward shifts in median cell length (Fig. 2B) and cell volume (Fig. 2C) compared to ST-intact placental tissue. ST-denuded vCTs tended to elongate along one axis, resulting in a downward shift in sphericity (Fig. 2D). This elongation event tended to be asymmetrical, resulting in a stronger polarized phenotype in ST-denuded vCTs. The median position of the nucleus in ST-intact vCTs was found to be halfway between the apical and basal membrane; however, the nuclear position in ST-denuded vCTs shifted closer to the basal membrane (Fig. 2E). On the apical surface, ST-denuded vCTs were found to have significantly increased apical volumes and higher frequencies of highly branched F-actin apical surface structure compared to ST-intact vCTs (Fig. 2F,G). Thus, ST denudation pushes vCTs to undergo morphological alterations, including changes in cell size, shape and polarity, thereby enriching a structurally distinct vCT subpopulation.

### Microvilli decorate the vCT apical surface *ex vivo*, *in vitro* and *in vivo*

**Fig. 3.**
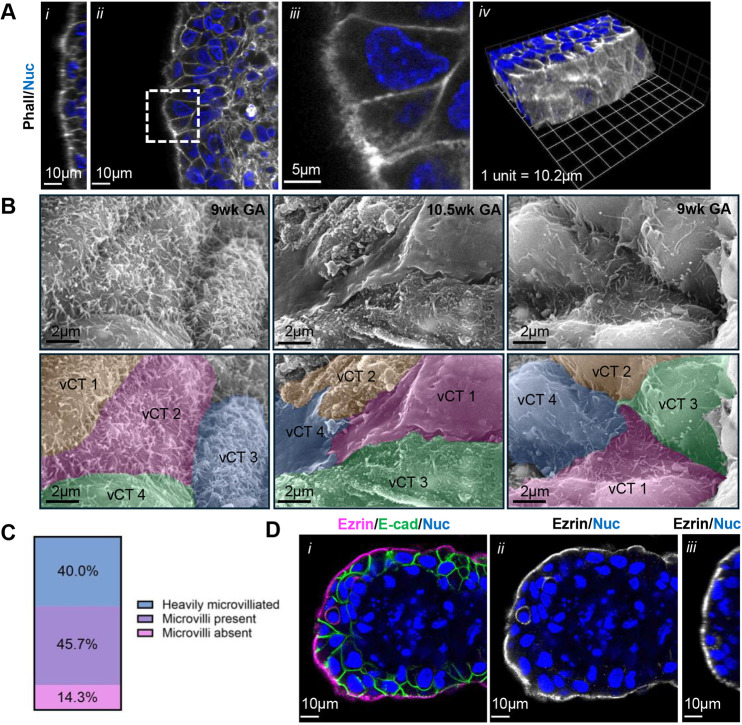
**Syncytiotrophoblast denuded villous cytotrophoblasts accumulate microvilli on their apical surface.** (A) Representative *zy*-plane (i), *xy*-plane (ii, iii) and 3D reconstituted (iv) confocal microscopy images of 12 weeks gestational age (GA) placental tissue 24 h post-trypsinization. Merged image of phalloidin (greyscale) and Hoechst (nuclei; blue) signals. Scale bars: 10 µm in i, ii; 5 µm in iii. One unit represents 10.2 µm in iv. (B) Representative SEM images of 9 weeks and 10.5 weeks GA explant tissue 24 h post-trypsinization. Lower panels are false-coloured images with colours marking individual villous cytotrophoblasts (vCTs). Scale bar: 2 µm. (C) Summary data for the proportion of heavily microvilliated vCTs, vCTs with microvilli present and vCTs with microvilli absent in placental explants 24 h post-trypsinization. *n*=70 cells. (D) Representative *xy*-plane confocal microscopy images (i, ii) of 9.3 weeks GA placental tissue 24 h post-trypsinization. (i) Merged image of anti-ezrin (magenta), anti-E-cadherin (green) and Hoechst (nuclei; blue) signals. (ii) Isolated anti-ezrin (greyscale) and Hoechst (nuclei; blue) signals. (iii) *zy* plane isolated anti-ezrin (greyscale) and Hoechst (nuclei; blue) signals. Scale bars: 10 µm. Images are representative of three placentas.

One of the most highly enriched features of vCTs in denuded explants was an increase in apical volume mostly attributable to the increased appearance of F-actin-positive hair-like projections at the apical surface (Figs 2A,F and 3A, Movie 1). Bundled F-actin in fusion-competent cells is central to membrane protrusion formation (Kim and Chen, 2019; Kim et al., 2015; Petrany and Millay, 2019; Sens et al., 2010; Shilagardi et al., 2013); therefore, we examined whether membrane projections were accumulating on the apical surface of explant vCTs. Scanning electron micrographs (SEMs) of the vCT surface at 24 h post-trypsinization revealed membranous protrusions ∼100 nm in diameter on the apical surface and bridging the intercellular gaps between individual cells (Fig. 3B). Microvilli are fine actin-based membrane projections (50-350 nm in diameter) (Orbach and Su, 2020), whereas podosomes, which are formed on fusion competent myoblasts, are broad actin-based membrane projections (0.5-2 μm in diameter) (Murphy and Courtneidge, 2011). Thus, based on their size, the vCT membrane projections fit the definition of microvilli. Interestingly, a wide variation in the degree of microvilliation of individual cells was observed (Fig. 3C).

Berryman et al. have previously reported immunogold labelling of ezrin, a core component of epithelial microvilli, within apical membrane projections of sporadic vCTs in intact placenta using transmission electron microscopy (TEM) (Berryman et al., 1993). Ezrin is crucial for the formation and stabilization of membrane protrusions (Saotome et al., 2004; Welf et al., 2020), particularly epithelial microvilli (Pelaseyed and Bretscher, 2018). Ezrin requires activation via phosphorylation of its Thr-567 residue to bind both the F-actin filament and overlying membrane (Zhu et al., 2007). Therefore, we stained ST-denuded explants to confirm whether a similar signal pattern of apically accumulated ezrin existed in our model. As expected, anti-ezrin signal strongly localized to the apical surface of both mononucleate vCTs and fused binucleate cells (Fig. 3D). Anti-ezrin signal was also strongly localized to the apical domain in explants at 72 h (Fig. S2), and variable signal was observed in the remaining mononucleate vCTs.

**Fig. 4.**
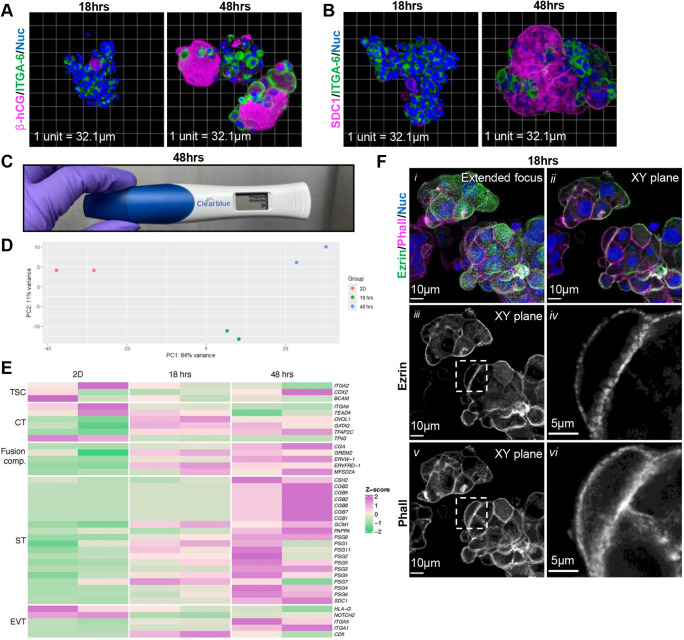
**Organoid model recapitulates apical villous cytotrophoblast microvilliation and syncytiotrophoblast development.** (A,B) Representative 3D reconstituted confocal microscopy images of organoids cultured for 18 h and 48 h in rotational culture stained with (A) anti-β-hCG (magenta), anti-ITGA6 (green) and Hoechst (nuclei; blue), and (B) anti-SDC1 (magenta), anti-ITGA6 (green) and Hoechst (nuclei; blue). One unit represents 32.1 µm. (C) Over-the-counter pregnancy test after application of 48 h organoid conditioned medium. (D) PCA plot of 2D trophoblast stem cell (TSC) culture, organoids at 18 h, and organoids at 48 h; PC1=variance due to culture conditions; PC2=variance due to sample. (E) Heatmap plot of 2D TSC culture, organoids at 18 h and organoids at 48 h based on z-score of trophoblastic markers. (F) Representative extended focus (i) and *xy*-plane (ii-vi) confocal microscopy images of organoids cultured for 18 h in rotational culture. (i, ii) Merged image of phalloidin (magenta), anti-ezrin (green) and Hoechst (nuclei; blue). (iii, iv) Isolated anti-ezrin (greyscale) signals. (iv) Higher magnification image of indicated region in iii. (v, vi) Isolated phalloidin (greyscale) signals. (vi) Higher magnification image of indicated region in v. Scale bars: 10 µm in i-iii; 5 µm in iv-vi. Images are representative of four experimental replicates (A,B,F). Data are pooled from two experimental replicates (D,E).

To identify if polarized microvillar localization is a conserved feature of differentiating vCTs, we used a TSC organoid model. In this model, TSCs (CT29) were passaged and cultured in a rotating wall vessel bioreactor in trophoblast organoid medium (Turco et al., 2018) for up to 48 h. 18 h after moving TSC into the bioreactor, cell aggregates were found to predominantly contain anti-ITGA6-positive mononucleate cells with isolated anti-β-hCG-positive or syndecan 1 (SDC1)-positive cells observed (Fig. 4A,B). After 48 h in the bioreactor, large cell aggregates with widespread anti-β-hCG- and anti-SDC1-positive multinucleate structures overlaying or adjacent to anti-ITGA6-positive mononucleate cells were consistently produced (Fig. 4A,B, Movies 2, 3), and secreted β-hCG could be readily detected in conditioned medium (Fig. 4C). To further characterize this model, we performed bulk RNA-seq analyses on 2D cultured TSCs and organoids after 18 h and 48 h in the bioreactor culture. PCA revealed segregation of these samples primarily by their culture conditions (Fig. 4D). DEG analyses revealed that, over time, bioreactor culture led to relative increases in the expression of transcripts associated with: fusion-competent vCTs (*CGA* and *GREM2*); fusogens, including syncytin 1 (*ERVW-1*), and syncytin 2 (*ERVFRD-1*) and its receptor *MFSD2A* (Malassine et al., 2007; Mi et al., 2000; Muir et al., 2006); and ST (*GCM1*, *CGB1*, *CGB2*, *PSG3*, *PSG4*, *PSG6*, *PAPPA*, *CSH2* and *SDC1*) (Fig. 4E). The expression of key EVT markers (*HLA-G* and *NOTCH2*) were relatively decreased by 48 h in bioreactor culture. Therefore, this model leads to the rapid formation of extensive ST and most likely mixed populations of vCTs. To identify if polarized microvilli on vCTs are also a feature of this model, cell aggregates were stained after 18 h in bioreactor culture (prior to ST development), and extensive F-actin and anti-ezrin signal could be observed primarily on cells on the outer surface of the aggregates (Fig. 4F, Movie 4) on their apical (outward facing) membranes. Interestingly, polarized anti-ezrin and branched F-actin positive structures were also observed at the borders between two or multiple cells in select regions within the organoids (Fig. 4F). Therefore, abundant polarized vCT microvilli are a morphological feature in this *in vitro* model of ST differentiation.

To compare our two models, we further analysed the bulk RNA-seq data from organoids at 18 h and 48 h, and explants at 24 h and 72 h. As expected, PCA and hierarchical cluster analyses revealed that samples clustered primarily by model type and secondarily by time in culture (Fig. S3A,B). Interestingly, DEG analyses comparing organoids to explants (Figs S4A and S5A) revealed that organoids had relatively higher expression of the ST markers *CGB, GCM1* and *SDC1* while explants had relatively higher expression of the ST markers *PAPPA, PSG* and *CSH2*. This suggests that the different models may bias vCT differentiation trajectory into transcriptionally distinct ST and/or that ST gene transcription varies in response to culture conditions. Gene ontology enrichment (GO pathway) terms associated with DEGs enriched in explants (Figs S4B and S5B) included ‘extracellular matrix components’ and ‘actin binding’, likely contributed by the non-trophoblastic stromal cells. GO pathway terms associated with DEGs enriched in organoids (Figs S4C and S5C) included ‘cadherin binding’, ‘histone kinase activity’ and ‘DNA helicase activity’. Thus, as expected, there are significant transcriptional differences between organoids and explants, with non-epithelial transcripts being relatively over-represented in explant transcriptomes, and organoid transcriptomes displaying the more proliferative and epithelial nature of the constituent cells.

**Fig. 5.**
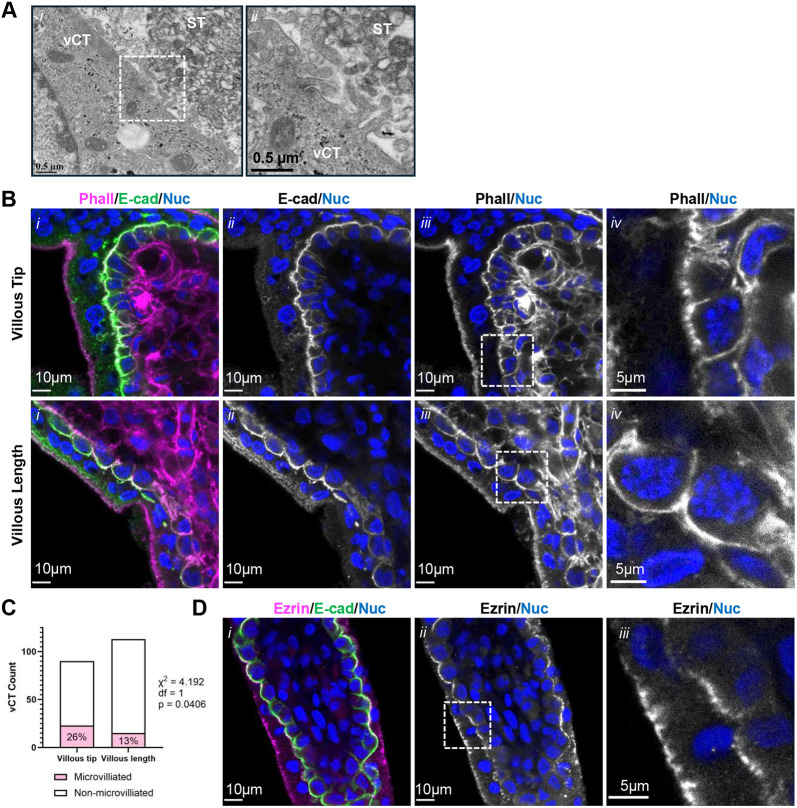
**Apical microvilli are present at the syncytiotrophoblast-villous cytotrophoblast interface in intact placental tissue.** (A) Representative TEM images of uncultured 12 weeks gestational age (GA) placental tissue. (ii) Higher magnification of the indicated region in i. Scale bars: 0.5 µm. Images are representative of one placenta. (B) Representative *xy*-plane confocal microscopy images of uncultured 10 weeks GA placental tissue at the villous tip and the villous length. (i) Merged images of phalloidin (magenta), anti-E-cadherin (green) and Hoechst (nuclei; blue) signals. (ii) Isolated anti-E-cadherin (greyscale) and Hoechst (nuclei; blue) signals. (iii) Isolated phalloidin (greyscale) and Hoechst (nuclei; blue) signals. (iv) Higher magnification images of indicated regions of isolated phalloidin (greyscale) and Hoechst (nuclei; blue) signals. Scale bars: 10 µm in i-iii; 5 µm in iv. (C) Summary counts of microvilliated and non-microvilliated villous cytotrophoblast (vCT) in uncultured placental tissue at the villous tips and the villous lengths. χ-squared test with Yate's correction. *n_villous tip_*=90 cells; *n_villous length_*=113 cells. (D) Representative *xy*-plane confocal microscopy images of uncultured 9.3 weeks GA placental tissue. (i) Merged images of anti-ezrin (magenta), anti-E-cadherin (green) and Hoechst (nuclei; blue). (ii) Isolated anti-ezrin (greyscale) and Hoechst signals. (iii) Higher magnification image of the indicated region in ii. Scale bars: 10 µm in i,ii; 5 µm in iii. Images are representative of three placentas*.*

We then sought to confirm whether vCT microvilli could be observed in intact first trimester placenta. Microvilliation of the vCT apical (ST-facing) membrane of a subset of cells has been observed in TEM studies of both term and first trimester placenta (Jones and Fox, 1991; Tashev et al., 2022). As expected, we observed vCTs with apical membrane projections at the vCT-ST interface with approximately the same diameter of those observed on the apical surface of vCTs in explants in TEM micrographs (Fig. 5A), though extensive microvilli were not clearly visible in the majority of vCTs, aligning with published data (Jones and Fox, 1991; Tashev et al., 2022). Staining intact tissue revealed that individual vCTs possess varying degrees of highly branched apical F-actin projections (Fig. 5B, Movies 5, 6). Towards the tip of individual villi, there was a doubling in the occurrence of highly branched and fine apical phalloidin signal in vCTs, while vCTs along the villus length more often displayed a smooth, unbranched apical phalloidin staining pattern (Fig. 5B,C). Consistent with the findings in explants, anti-ezrin signal variably localized to the apical surface of individual vCTs and, as previously reported, in a discontinuous pattern at the apical surface of the ST (Fig. 5D) (Berryman et al., 1993; Patel et al., 2023). Altogether, these *ex vivo*, *in vitro* and *in vivo* data suggest that the formation of extensive polarized microvilli on the vCT surface represents a morphological adaptation most likely associated with a fusion-competent state.

### Microvillar stabilization via activated ezrin is needed for vCT fusion and differentiation

**Fig. 6.**
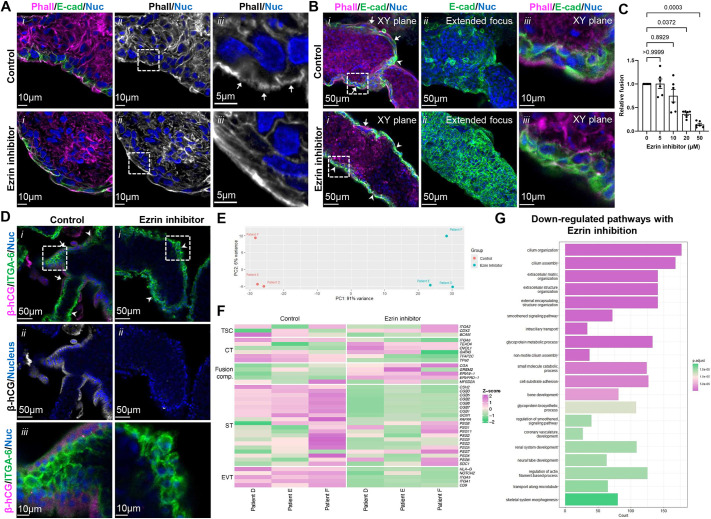
**Disruption of microvilli via ezrin inhibition impairs villous cytotrophoblast fusion and markers of functional villous cytotrophoblast differentiation.** (A) Representative *xy*-plane confocal microscopy images of 9 weeks gestational age (GA) explants pulsed for 2 h with or without 50 µM ezrin inhibitor. (i) Merged images of phalloidin (magenta), anti-E-cadherin (green) and Hoechst (nuclei; blue) signals. (ii) Isolated phalloidin (greyscale) and Hoechst (nuclei; blue) signals. (iii) Higher magnification images of regions indicated in ii. Scale bars: 10 µm in i,ii; 5 µm in iii. Arrows indicate protrusive microvilli. (B) Representative *xy*-plane (i, iii) and extended focus (ii) confocal microscopy images of 9.3 weeks GA explants cultured for 72 h with or without 50 µM ezrin inhibitor. (i) Merged images of phalloidin (magenta), anti-E-cadherin (green) and Hoechst (nuclei; blue) signals. (ii) Isolated anti-E-cadherin (green) and Hoechst (nuclei; blue) signals. (iii) Higher magnification images of regions indicated in i. Scale bars: 50 µm in i,ii; 10 µm in iii. Arrows indicate regenerated syncytiotrophoblast (ST) regions; arrowheads indicate unfused villous cytotrophoblast (vCT) regions. (C) Summary data of dose-dependent ezrin inhibitor treatment on fusion (change in ST:vCT) normalized to vehicle controls. Data are mean±s.e.m. Unpaired Kruskal–Wallis test with Dunnett's multiple comparisons. *n*=6. Fusion (change in ST:vCT) was calculated using Eqn 3 (see Materials and Methods). (D) Representative *xy*-plane confocal microscopy images of 11.0 weeks GA explants cultured for 48 h with or without 50 µM ezrin inhibitor. (i) Merged images of anti-β-hCG (magenta), anti-ITGA6 (green) and Hoechst (nuclei; blue) signals. (ii) Isolated anti-β-hCG (greyscale) and Hoechst (nuclei; blue) signals. (iii) Higher magnification image of regions indicated in i. Scale bars: 50 µm in i,ii; 10 µm in iii. Arrows indicate regenerated ST regions; arrowheads indicate unfused vCT regions. (E) PCA plot of explants cultured for 48 h in total with or without 50 µM ezrin inhibitor. PC1 indicates variance due to treatment; PC2 indicates variance due to sample. (F) Heatmap plot of explants cultured for 72 h in total with or without 50 µM ezrin inhibitor based on the z-score of trophoblastic markers. (G) GO pathway analyses on downregulated biological processes in explants cultured for 48 h with or without 50 µM ezrin inhibitor. All DEGs analysed had a *P*-adjusted value of <0.05 and a log2 fold change <−0.5. Images are representative of three (A,D) and six (B) placentas. (C,E-G) Data are from six (C) and three (E-G) experimental replicates.

Since vCT microvilliation is a conserved feature *in vivo*, *in vitro* and *ex vivo* across multiple models, we hypothesized that formation of microvilli may be supportive of trophoblast fusion. To target microvilli without disrupting whole-cell actin dynamics, we treated vCTs with an ezrin inhibitor (NSC668394), which binds directly to ezrin to prevent its phosphorylation at the Thr-567 residue that is necessary for its activation, membrane binding and microvillar stabilization (Viswanatha et al., 2012; Welf et al., 2020). When ST-denuded explants were treated with ezrin inhibitor for 2 h, there was significant loss of apical anti-phospho-ezrin signal (Fig. S6) and a smoothening of the F-actin network at the apical membrane (Fig. 6A), showing that ezrin inhibition leads to the destabilization of apical microvilli. Ezrin inhibitor treatment did not significantly alter explant vCT EdU incorporation (Fig. S7) or vCT death, as determined by anti-active caspase 3 signal (Fig. S8). Crucially, treatment of ST-denuded explants with ezrin inhibitor for 48 h dose-dependently blocked vCT fusion by up to 86% (Fig. 6B,C), disrupted the appearance of β-hCG-positive ST (Fig. 6D) and significantly inhibited *CGB1*, *CGB2* and *CGB3* transcription (Fig. 6F, Fig. S9A). Therefore, blocking ezrin activation strongly inhibits vCT fusion in explants and leads to a loss of apical microvilli.

Cell shape or, more specifically, a decrease in cell surface area post-fusion, has been shown to reinforce transcriptional regulation of cell differentiation in fused cells (Feliciano et al., 2021). Multiple trophoblast fusion and differentiation studies using the BeWo cells and primary vCTs have identified that trophoblast fusion and functional differentiation, or at least the production of β-hCG, are isolatable processes (Collett et al., 2012; Johnstone et al., 2005; Orendi et al., 2010). Therefore, we performed bulk RNA-sequencing to examine the relative expression of additional trophoblastic genes between control and ezrin inhibitor-treated explants. PCA revealed a strong effect of ezrin inhibition on gene expression profiles (Fig. 6E). DEG analyses revealed that ezrin inhibitor treatment decreased the relative abundance of transcripts associated with ST and EVT, and increased transcript abundance associated with fusion competent vCTs (Fig. 6F). Changes in the expression of *CGB*, *GCM1* and the fusogens *ERVFRD-1* and *ERVW-1* were recapitulated using RT-PCR (Fig. S9). GO pathway terms for downregulated genes with ezrin inhibition included ‘regulation of actin filament-based processes’, ‘glycoprotein biosynthetic processes’, and ‘extracellular matrix and structure organization’ (Fig. 6G). GO pathway terms for upregulated genes with ezrin inhibition included ‘ribosome biogenesis’, ‘RNA splicing’ and ‘protein-RNA complex organization’ (Fig. S10). Taken together with the significant decrease in cell fusion, these data suggest destabilization of microvilli blocks both functional and transcriptional ST differentiation in explants.

**Fig. 7.**
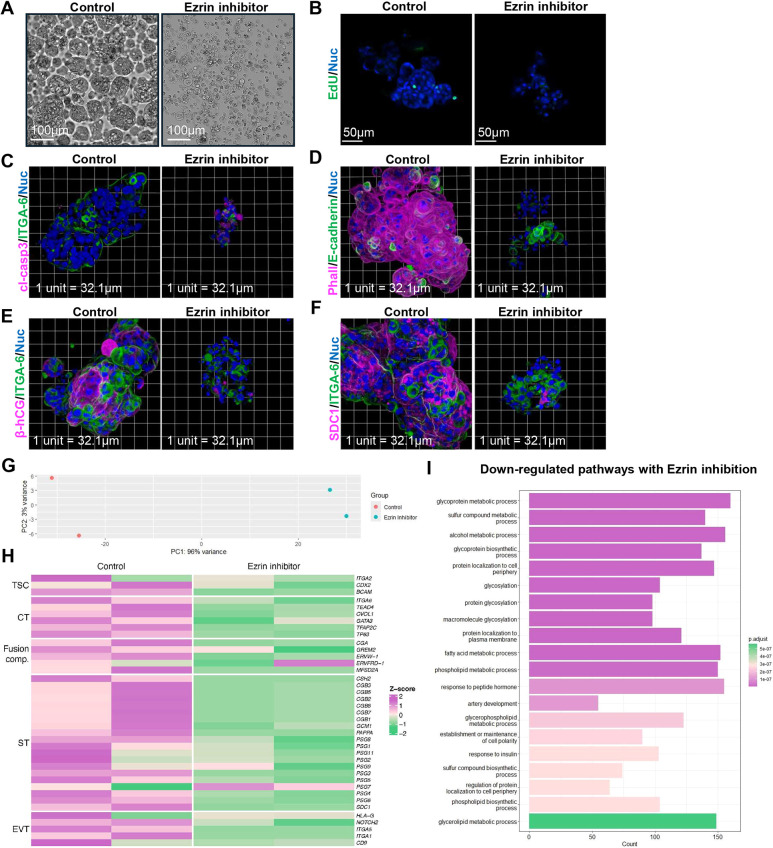
**Ezrin inhibition disrupts syncytiotrophoblast formation in organoids.** (A) Representative bright-field images of organoids cultured for 48 h with or without 25 µM ezrin inhibitor in rotational culture. Scale bars: 100 µm. (B-F) Representative *xy* plane (B) and 3D reconstituted (C-F) confocal microscopy images of organoids cultured for 48 h with or without 25 µM ezrin inhibitor in rotational culture stained with Hoechst (nuclei; blue) and (B) EdU (green), (C) anti-cleaved-caspase-3 (magenta) and anti-ITGA6 (green), (D) phalloidin (magenta) and anti-E-cadherin (green), (E) anti-β-hCG (magenta) and anti-ITGA6 (green), and (F) anti-SDC1 (magenta) and anti-ITGA6 (green). Scale bars: 50 µm in B. One unit represents 32.1 µm in C-F. (G) PCA plot of organoids at 48 h with or without 25 µM ezrin inhibitor. PC1 indicates variance due to treatments; PC2 indicates variance due to sample. (H) Heatmap plot of organoids at 48 h with or without 25 µM ezrin inhibitor based on z-score of trophoblastic markers. (I) GO pathway analyses on downregulated biological processes of organoids at 48 h with or without 25 µM ezrin inhibitor. All DEGs analysed had a *P*-adjusted value of <0.05 and a log2 fold change <−0.5. Images are representative of four experimental replicates (A-F). (G-I) Data are from two experimental replicates.

In organoids, exposure to ezrin inhibitor profoundly and reproducibly decreased organoid size at 48 h (Fig. 7A). Proliferation, as visualized by EdU incorporation, was reduced (Fig. 7B), and increased cell death determined by anti-active-caspase-3 signal (Fig. 7C) was observed with ezrin inhibitor treatment. With ezrin inhibition, organoids were a mix of anti-ITGA6- or anti-E-cadherin-positive mononucleate cells and aggregated cells with hyper-condensed nuclei (Fig. 7C-F). These cells with hyper-condensed nuclei are characterized by anti-active-caspase 3 positivity and a weak anti-ITGA6, anti-E-cadherin or phalloidin signal (Fig. 7C-F). Crucially, limited signals for anti-β-hCG (Fig. 7E) and anti-SDC1 were observed with ezrin inhibitor treatment (Fig. 7F). Bulk RNA-sequencing was performed on control and ezrin inhibitor-treated organoids where PCA revealed that sample clustering was primarily attributable to treatment condition (Fig. 7G). DEG analyses revealed a broad relative decrease in transcript abundance for all trophoblastic markers (vCTs, EVTs and ST) with ezrin inhibition (Fig. 7H). Interestingly, GO pathway terms for downregulated genes with ezrin inhibition included ‘glycosylation’, ‘protein localization to the cell periphery’ and the ‘maintenance and establishment of cell polarity’ (Fig. 7I), suggesting that ezrin activation has strong effects on the trafficking of proteins to the plasma membrane. Like the explant model, GO pathway terms for upregulated genes in organoids with ezrin inhibition included ‘ribosome biogenesis’, ‘RNA splicing’ and ‘protein-RNA complex organization’ (Fig. S11). In summary, despite differences in the effect of ezrin inhibition between models, both explants and organoids had profoundly disrupted ability to form ST with microvillar destabilization.

### Disruption of microvilli alters polarized endosomal trafficking

**Fig. 8.**
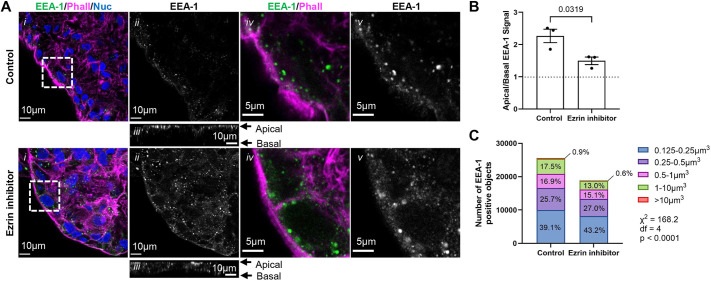
**Ezrin inhibitor treatment disrupts polarized endocytosis at the apical villous cytotrophoblast domain.** (A) Representative *xy* plane (i, ii, iv, v) and *xz* plane (iii) confocal microscopy images of 9 weeks gestational age (GA) explants pulsed for 2 h with or without 50 µM ezrin inhibitor at 24 h post-trypsinization. (i) Merged image of anti-EEA-1 (green), phalloidin (magenta) and Hoechst (nuclei; blue) signals. (ii) Isolated anti-EEA-1 (greyscale) signal. (iii) *xz* plane isolated anti-EEA-1 (greyscale). (iv) Higher magnification image of regions indicated in i with isolated anti-EEA-1 (green) and phalloidin (magenta) signals. (v) Higher magnification image of regions indicated in i with isolated anti-EEA-1 (greyscale) signal. Scale bars: 10 µm in i-iii; 5 µm in iv, v. (B) Summary data of the ratio of apical:basal EEA-1 signal of explants pulsed for 2 h with or without 50 µM ezrin inhibitor at 24 h post-trypsinization. Apical:basal signal was calculated using Eqn 4 (see Materials and Methods). Data are mean±s.e.m., unpaired parametric Student's *t*-test, *n*=3. (C) Summary counts of the EEA-1-positive objects binned by size. χ-squared test; *n_control_*=25,573 objects; *n_ezrin inhibitor_*=18,858 objects. Images are representative of three placentas.

Having shown that microvillar stabilization facilitates ST formation, we were interested in investigating how microvilli may be supporting vCT fusion. Given the established role of microvilli as signalling platforms in other microvilliated cell types (Kim et al., 2018; McConnell et al., 2009), it is possible that microvilli serve to spatially restrict or amplify specific signalling cascades within trophoblasts. Pidoux et al. (2014) have previously shown that ezrin functions as an A-kinase anchoring protein (AKAP) in fusing 2D primary vCT cultures, serving as a scaffolding protein to protein kinase A (PKA), an important regulator of vCT fusogen expression (Gerbaud et al., 2015; Pidoux et al., 2014). Therefore, PKA activity may be spatially restricted to microvilli. Ezrin, in its role as a tethering molecule between the cytoskeleton and the membrane, is also very important for regulating membrane tension (Rouven Bruckner et al., 2015). It is well established that changes in membrane tension alter endocytosis and exocytosis (Dai and Sheetz, 1995; Diz-Munoz et al., 2013; Ferguson et al., 2017; Morris and Homann, 2001; Pontes et al., 2017; Rouven Bruckner et al., 2015). Hence, the polarized accumulation of vCT ezrin may function to regulate vesicular trafficking. Trafficking vesicles mediate and regulate key cell signalling molecules, including, but not restricted to, receptor tyrosine kinases, G-protein coupled receptors and mediators of the MAPK pathway (Dobrowolski and De Robertis, 2011). High-magnification z-stack confocal images of denuded explants pulsed with vehicle control or ezrin inhibitor were stained using anti-early endosome antigen 1 (EEA-1), a tethering molecule that localizes to early endosomes and has polarized distribution in non-trophoblastic epithelia (Wilson et al., 2000). Anti-EEA-1 signal was strongly localized within microvillar structures, indicating that vCT microvilli are important sites for endocytic uptake and endosomal-localized signalling (Fig. 8A). In ezrin inhibitor-treated explants, anti-EEA-1 signals were less restricted to the apical domain compared to controls (Fig. 8A,B) and increased numbers of small puncta were observed in the lateral and basal regions of vCT. Indeed, there was a significant shift towards smaller volume EEA-1-positive puncta with ezrin inhibitor treatment (Fig. 8C). Altogether, these data suggest that apical microvilli are important for organizing endocytic activity.

### Apically polarized CD98 is essential for vCT fusion and differentiation

**Fig. 9.**
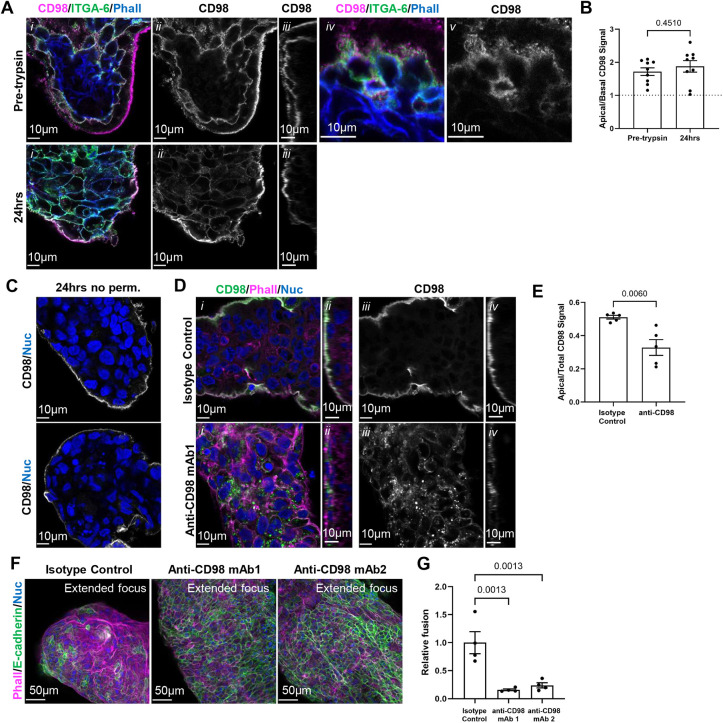
**Disrupting apically localized CD98 impairs villous cytotrophoblast fusion.** (A) Representative *xy*-plane (i, ii, iv, v) and *zy*-plane (iii) confocal microscopy images of pre-trypsinized 10 weeks and 9 weeks gestational age (GA) tissue and 24 h post-trypsinization tissue. (i) Merged image of anti-CD98 (magenta), anti-ITGA6 (green) and phalloidin (blue) signals. (ii) Isolated anti-CD98 (greyscale) signals. (iii) Isolated anti-CD98 (greyscale) signals. (iv) Higher magnification merged image of anti-CD98 (magenta), anti-ITGA6 (green) and phalloidin (blue) signals. (v) Higher magnification isolated anti-CD98 (greyscale) signal. Scale bars: 10 µm. (B) Summary data of the ratio of apical:basal CD98 signal of placental tissue in pre-trypsinized control tissue versus 24 h post-trypsinization. Data are mean±s.e.m., unpaired parametric Student's *t*-test, *n*=9. Apical:basal signal was calculated using Eqn 4 (see Materials and Methods). (C) Representative *xy*-plane confocal microscopy images of non-permeabilized 12 weeks GA placental tissue at 24 h post-trypsinization stained with anti-CD98 (greyscale) and Hoechst (nuclei; blue). Scale bars: 10 µm. (D) Representative confocal microscopy images of 10 weeks GA placental tissue cultured for 48 h total with or without cross-linked anti-CD98 mAb 1 or isotype control. (i, ii) Merged image of anti-CD98-FITC (green), phalloidin (F-actin; magenta) and Hoechst (nuclei; blue) signals. (iii, iv) Isolated anti-CD98-FITC (greyscale) *xy*-plane signal. Scale bars: 10 µm. (E) Summary data of the ratio of apical:total CD98 signal of explants cultured for 48 h with or without cross-linked anti-CD98 mAb 1 or isotype control. Data are mean±s.e.m., unpaired Student's *t*-test, *n*=5. Apical:total signal was calculated using Eqn 5 (see Materials and Methods). (F) Representative extended focus confocal microscopy images of 10 weeks GA explants cultured with or without cross-linked anti-CD98 mAb 1 and anti-CD98 mAb 2, or an isotype control stained with phalloidin (magenta), anti-E-cadherin (green) and Hoechst (nuclei; blue). Scale bars: 50 µm. (G) Summary data of anti-CD98 treatment on fusion [change in syncytiotrophoblast (ST): villous cytotrophoblast (vCT)] normalized to isotype controls. Data are mean±s.e.m., unpaired one-way ANOVA with Tukey's multiple comparisons, *n*=4. Fusion (change in ST:vCT) was calculated using Eqn 3 (see Materials and Methods). Images are representative of nine (A), five (C,D) and four (F) placentas. (B,E,G) Data are from nine (B), five (E) and four (G) experimental replicates.

Apical microvilli may also be crucial for the accumulation of fusion-promoting proteins. GO pathway analyses of bulk RNA-seq data from ezrin inhibitor-treated explants and organoids highlighted that disruption of microvillar stability altered glycosylation and processing of glycoproteins (Figs 6G and 7I). Transmembrane glycoproteins play crucial roles in membrane fusion (Gong et al., 2005; Vargas et al., 2009; White et al., 2008). CD98 (fusion regulatory protein 1) is a transmembrane glycoprotein with pleiotropic functions, including the regulation of cell and viral fusion, amino acid transport, and integrin signalling (Cantor and Ginsberg, 2012; Kudo et al., 2003; Renaud and Jeyarajah, 2022). Knockdown of CD98 in BeWo cells impaired fusion (Kudo et al., 2003). Previous work has shown that anti-CD98 signal strongly localized to the apical surface of the ST and to the ST-vCT interface of term tissue (Kudo et al., 2003). Therefore, we posited that CD98 would be localized to vCT microvilli and that disruption of the localization of this microvilli-accumulated protein would impact fusion. Consistent with previous findings, anti-CD98 signal was enriched at the apical, microvillar domain and sparsely present in the vCT basolateral domain in both ST-denuded and ST-intact tissue (Fig. 9A,B). There was also a visible increase in anti-CD98 signal within densely branched microvillIi on vCT in intact tissue and in ST-denuded explants (Fig. 9A). To selectively relocalize apical CD98, we performed monoclonal antibody cross-linking experiments that have been shown to influence β1-integrin signalling and lead to loss of CD98 membrane localization (Dalton et al., 2007; Liao and Cantor, 2016; Rintoul et al., 2002). Explants were treated with isotype controls or anti-CD98 at 24 h post-trypsinization followed by a cross-linking secondary antibody in culture. To confirm that this would selectively target the apical pool of CD98 in vCTs, we stained non-permeabilized untreated explants at 24 h with the same monoclonal anti-CD98 and found that the signal was restricted to the apical domain under these conditions (Fig. 9C). Anti-CD98 cross-linking caused a decrease in the apical anti-CD98 signal and the appearance of bright anti-CD98-positive puncta within the vCT, as observed with a separate anti-CD98-FITC antibody (Fig. 9D,E). Importantly, apical CD98 cross-linking strongly impaired vCT fusion at 72 h (Fig. 9F,G). Therefore, the apical microvillar localization of this membrane glycoprotein is crucial for regulating vCT fusion. Altogether, these data and the results showing the enrichment of endosomes within the apical domain highlight that polarization of membrane glycoproteins, and endosome-localized cell signalling at the apical microvillar domain, may be requisite steps for efficient syncytialization.

## DISCUSSION

Proper formation and maintenance of the ST throughout gestation is crucial for placental function and significantly contributes to healthy pregnancy progression. Diminished vCT fusion capacity is a feature of pregnancy complications like IUGR and pre-eclampsia (Gauster et al., 2009; Langbein et al., 2008; Ruebner et al., 2010), which are associated with increased perinatal morbidity, mortality, and lifelong increased risk of cardiovascular and neurological disease for affected infants (Burton et al., 2016). Therefore, a thorough understanding of the molecular regulation of vCT fusion could allow the development of treatments for placental pathologies and improve lifelong health. We found that the stabilization of microvillar membrane protrusions within vCT is necessary for successful fusion between neighbouring cells for ST formation and/or regeneration, and suggest that vCT microvilli may serve to spatially focus endosomal localized signalling. Additionally, we found that apical microvillar localization of a key membrane glycoprotein is necessary to support fusion. All these data together have unveiled that assembly of microvillar domains is a crucial process to support efficient ST formation.

Here, we have presented two 3D models of ST development: a first trimester human placental explant model and an organoid model. Our explant model exploits the spontaneous fusion capacity of vCTs when overlying syncytium is lost. Regions of localized ST denudation and subsequent fibrin deposition are a feature of both healthy and pathological placentas (Scifres and Nelson, 2009); therefore, this model more accurately represents ST regeneration than ST maintenance. A key consideration with this model is the inherent donor-to-donor variability in fusion capacity and transcriptional markers of ST differentiation. Variations in transcriptional signatures could reflect nuclear heterogeneity and varying stages of ST nuclear development, which has been recently seen in single nuclei sequencing in human placenta (Wang et al., 2024). Therefore, explants require proper normalization to donor-matched controls and sufficient biological replicates. Despite this, there is the advantage of preserving native villous structures, including, but not limited to, the composition of heterogeneous trophoblastic subpopulations, the presence of non-trophoblastic cells and the extracellular matrix. Biological sex can also influence cellular processes (Mank and Rideout, 2021). Though we could not determine the sex of every sample used in the study (Table 1), no sex-specific trends were observed in any analyses where sex data were available. The TSC organoids are a comparatively more reductionist 3D model of ST development. This model also leads to spontaneous ST formation and has the advantage of being composed entirely of trophoblastic cells, simplifying global analyses by eliminating stromal cell contributions. It is also useful for vCT proliferation experiments. A physiological aspect that we did not emulate with either model was the hypoxic environment of the first trimester placental compartment, which is known to affect trophoblast differentiation (Chang et al., 2018) and will be important to consider in the future.

**Table 1.** Tissue and donor characteristics

	Placentas*	Donors
Age (mean±s.d.)	10.3±1.1 weeks	26.3±5.4 years
Range (min, max)	(9, 13)	(18, 38)
Count	51	50

*The biological sex of the 26 placentas used was 53.8% female and 46.2% male.

Our analyses and the distinction between results obtained with our models highlight the pleiotropic and context-dependent functions of ezrin. Ezrin has been shown in other cells to interact with the EGF/EGFR (Saygideger-Kont et al., 2016), PI3K/Akt (Gautreau et al., 1999; Li et al., 2019) and Rho GTPase (Hirao et al., 1996; Prag et al., 2007; Takahashi et al., 1997) pathways, thereby potentially affecting cell proliferation, survival and motility. In the organoid model, ezrin inhibition significantly impaired organoid size via decreased cell proliferation, viability and extracellular matrix component production, and likely decreased cell aggregation through the loss of actin-mediated membrane projections necessary for effective cell adhesion (Gershman and Rosen, 1978). Comparison of the up- and downregulated GO pathways shows conserved and non-conserved terms between the explant and organoid models with ezrin inhibition. A key consideration when comparing these two models transcriptionally is the contribution of non-trophoblastic populations present in the explant model, as highlighted by our comparison of the models. Hence, use of multiple models to investigate conserved pathways necessary for ST development is crucial.

Importantly, although our cell shape factor analyses revealed that ST denudation shifts vCT median characteristics towards extremes, the majority of vCTs in denuded tissue still falls within the range of those observed in intact tissue. Thus, cellular features associated with fusion competence are likely accentuated by a lack of negative feedback from the ST. Data from explants and organoids support the conserved formation of microvilli prior to fusion. Microvilliation has also been observed by others during 2D vCT fusion (Bax et al., 1989), and microvilliated vCT have been observed in intact term placental sections with TEM (Jones and Fox, 1991), supporting that this may be a conserved feature of vCT fusion competence across gestation. Importantly, the apical polarization of vCT microvilli was not exclusively seen in organoids. Instead, areas of accumulated microvilli between adjacent cells were observed. This supports our conclusion that polarized localization of microvilli is crucial for vCT differentiation and fusion, though the necessity for apical localization does not appear to be wholly conserved. Our functional analyses show that disrupting microvilli blocks fusion and impairs differentiation. It has also recently been shown that increasing microvillar formation via flow-mediated shear stress enhances fusion in iPSC-derived trophoblasts (Inohaya et al., 2024). Hence, microvilliation is obligate for trophoblast fusion and likely for further ST development. But our *ex vivo* and *in vitro* data do not support that the absence and/or presence of polarized microvilli could be used to identify fusion competent vCT, or at least not a discrete population of transcriptionally unified cells like those revealed by single-cell and single-nuclei RNA-sequencing analyses.

Our analyses highlight that vCT accumulation of microvilli may serve to localize crucial pro-fusogenic glycoproteins and endosomal signalling within a highly specialized cellular compartment in a polarized manner within the cell. Intestinal microvilli contain their own sub-proteome (McConnell et al., 2011) and the polarized localization of proteins within highly specialized structures may be driven by the differential composition of the microvillar lipid bilayer within highly curved and tightly packed structures (Cebecauer, 2021; Ikenouchi et al., 2013). The concept of microvilli as signalling centres is established in non-fusing cell types such as intestinal epithelia and T-cells (Kim et al., 2018; McConnell et al., 2009). Pidoux et al. (2014) have previously shown the role of activated ezrin in localizing PKA and/or connexin 43 binding, allowing the formation of gap junctions between adjacent trophoblasts to facilitate signal synchronization prior to fusion. Therefore, our data support a broad base of knowledge across cell types about the importance of this cellular compartment for overall function and differentiation. Our RNA-seq analyses in explants and organoids unexpectedly linked ezrin activation to the regulation of glycosylation at the transcriptional level. Whether this is due to direct functions of ezrin itself or due to indirectly regulated changes in cytoskeletal dynamics caused by changing the degree of cytoskeletal-membrane tethering will be important to determine in the future. Our work and that of Pidoux et al. have highlighted that ezrin is a key regulator for vCT differentiation, but ezrin clearly regulates multiple simultaneous pathways and processes. Future work is necessary to understand the relative contribution of ezrin and its effects on glycosylation to vCT fusion, particularly considering the importance of glycosylation for the fusogenic activity of syncytin 2 (Cui et al., 2016). Our data showing that disruption of the polarized and microvillar localization of the glycoprotein CD98 impairs vCT fusion supports the hypothesis that not only ezrin activation, but also the molecular organization of multiple factors within the microvillar compartment are necessary to support ST development. RNA-seq analyses also revealed a conserved effect of ezrin inhibitor treatment on RNA processing and ribosome biogenesis. Work with another ezrin inhibitor identified that ezrin directly interacts with an RNA-binding protein when in its inactive form (Celik et al., 2015). Therefore, the accumulation of inactivated ezrin in our experiments may also negatively influence vCT differentiation, highlighting that the polarized localization and activation of ezrin may underpin efficient vCT differentiation via multiple mechanisms.

Other fusing cell systems have shown the importance of increasing membrane-membrane contact sites for fusion (Brukman et al., 2019; Kim and Chen, 2019; Wilson and Snell, 1998). Hence, microvilli may serve to increase the apical surface area of vCT for this purpose. E-cadherin signal was strongly accumulated in proximity to the observed apical ezrin signal in our experiments, and others have observed the gap junction protein connexin 43 in a similar pattern (Cronier et al., 2002); thus, microvilli may facilitate increased contact points by concentrating junctional protein interactions between the vCT and overlying ST. Due to the proximity of vCT microvilli to the ST membrane, it is tempting to suggest that fusion pores may also arise in microvillar tips. Syncytin 2 has a non-polarized membrane localization in select vCTs *in vivo* (Esnault et al., 2008), but as a class I fusogen (Vance and Lee, 2020), it requires engagement with its receptor, major facilitator superfamily domain containing 2 (MFSD2) (Esnault et al., 2008), to insert into the adjacent cell membrane and facilitate fusion pore formation. Thus, microvilliation may allow more efficient syncytin 2 and MFSD2 engagement due to increased contact sites. MFSD2 is expressed on both the ST and CT (Esnault et al., 2008), therefore increasing the contact points between vCT syncytin 2 and ST MFSD2 would increase the likelihood of fusion.

The polarized accumulation of microvilli at the apical surface or between adjacent cells supports a model whereby the initiation of a polarity network amplifies a pro-fusogenic vCT state and dictates the position of the newly formed ST. But ezrin localization in 2D cultured primary vCT was dispersed throughout the membrane (Pidoux et al., 2014). This suggests that substrate stiffness or vCT interactions with extracellular matrix components may support polarization and enhance fusion. Our previous work has shown that the canonical apical-basal polarity regulating factor atypical protein kinase-c (aPKC) is dispersedly expressed within first trimester vCT (Shaha et al., 2022), suggesting an alternative pathway may be activated to reinforce vCT polarity during differentiation. Understanding the factors that initiate vCT polarity and microvillus formation will be an interesting future direction.

We chose to perform our analyses in 9-13 weeks gestational age first trimester tissue due to the continuous nature of the vCT layer at this point in gestation, thereby allowing us to analyse the greatest number of cells. However, throughout gestation it is known that the placental microenvironment greatly changes, likely affecting vCT morphology and differentiation. One such example is the changing oxygen tension and shear stress dynamics that occurs with altered blood flow through the remodelled spiral arteries in the second trimester (Burton, 2009; Greenbaum et al., 2023). With advancing gestation it is known that vCT cell shape changes from a cuboidal to a flattened phenotype (Jones and Fox, 1991). Despite changes in cell shape parameters, certain obligate processes in fusion such as fusogen expression and engagement must be conserved. Microvilliated vCTs in term tissue have been observed in intact term placental sections with TEM (Jones and Fox, 1991), supporting that microvilliation may be a conserved feature of vCT fusion competence across gestation. Future work is necessary to explore whether fusion competent vCTs beyond the first trimester have morphological features and fusion mechanisms that are shared with first trimester cells. Differences in the way vCTs execute fusion across gestation are possible, and understanding their conserved and divergent regulation would inform development of treatments in the future.

In summary, we show that the stabilization of vCT microvilli and the polarized localization of specific proteins within the microvillar compartment is crucial for fusion and formation of the ST. Microvilli are a conserved site of fusion throughout evolution seen in cell-fusion events in species as diverse as sea urchin and *Drosophila*. Yet understanding why these structures are necessary and how their emergence and stabilization is regulated to control one of the most dramatic events in cell biology is lacking. Future work to address the temporal and spatial regulation of the cortical actin cytoskeleton to regulate microvilli emergence and localization will be particularly important.

## MATERIALS AND METHODS

### Tissue collection

Nine to 12 weeks gestational age human placental samples were collected by methods approved by the University of Alberta Human Research Ethics Board (Pro00089293). First trimester placental tissue was obtained from elective surgical pregnancy terminations following informed consent by donors. For donor characteristics, please see Table 1. A list of all samples used in the study is included in Table S1.

### ST regeneration model and treatments

Placental samples were cut into 2-3 mm^3^ explants in ice-cold phosphate-buffered saline (PBS) and then either fixed immediately as uncultured pre-trypsin controls or trypsinized and cultured. For trypsinization, tissue was digested with 0.25% trypsin-EDTA (Gibco, 25200-072) for 7 min at 37°C. The reaction was quenched using 10% heat-inactivated fetal bovine serum (FBS; Wisent, 098150) and a single explant was placed in each well of a 48-well plate in medium consisting of Iscove's Modified Dulbecco's Medium (IMDM) (Gibco, 12440-053), 5% heat-inactivated FBS (Wisent, 098150), 50 µg/ml gentamicin (Gibco, 15710-064) and 1× ITS-X supplement (Gibco, 51500-056) at 37°C in 5% CO_2_. Medium was changed 24 h post-trypsinization to remove stripped ST debris. Explants were cultured for up to 72 h before processing for downstream analyses. Three to 12 explants (technical replicates) from different areas of the placenta were cultured per treatment group for all experiments. Twelve explants/treatment were used for downstream RNA isolation and analyses.

For ezrin inhibitor treatments, 5-50 µM of NSC668394 (EMD Millipore, 341216, lot 3934373) or vehicle control (DMSO) was added to medium 24 h post-trypsinization for 2-48 h.

For anti-CD98 cross-linking experiments, 20 µg/ml of anti-CD98 monoclonal antibody 1 (Biolegend, 315602, lot B331096), anti-CD98 monoclonal antibody 2 (BD Biosciences, 556074, lot 4015742) or isotype controls (Biolegend, 400102, lot B379844) was added to medium 24 h post-trypsinization for 1 h at a concentration of 0.5 mg/ml. Explants were washed three times with ice-cold medium. Goat anti-mouse IgG (Jackson ImmunoResearch, 115-005-062, lot 169750) was added to medium and incubated for 1 h at a concentration of 1.8 mg/ml. Explants were washed with ice-cold medium and then incubated for 24-48 h.

Proliferation was determined via incorporation of EdU into the nuclei using a Click-iT Plus EdU Alexa Fluor 488 Imaging kit (Invitrogen, C10637, lot 2146725). 10 µM of EdU was added to medium and explants were incubated for 1 h. Explants were fixed using 4% paraformaldehyde (PFA) on ice for 2 h. EdU was detected according to kit instructions.

### Tissue staining

Placental tissue (uncultured and cultured explants) was fixed using 2% PFA overnight at 4°C or 4% PFA on ice for 2 h. When necessary, heat-induced antigen retrieval was conducted using 0.01 M sodium citrate buffer at pH 6.0. Fixed tissue was blocked and permeabilized in blocking buffer (5% normal donkey serum, 0.5% Triton-X100, 1:100 human IgG (Invitrogen, 02-7102, lot WI327305). Tissue was then incubated overnight at 4°C in blocking buffer with primary antibodies. For antibody information, please see Table S2. Tissue was then washed with PBS and 0.05% Tween (PBST) and PBS, and incubated with the appropriate fluorescent secondary antibodies AlexaFluor 488 (Invitrogen) and AlexaFluor 594 (Invitrogen) and/or stained with 0.17 µM phalloidin-iFluor 594 (AAT Bioquest; Pleasanton, 23122). Tissue was then incubated with 10 µg/ml Hoechst 33342 (Thermo Fisher Scientific, H3570) followed by PBS washes and whole-mounted with imaging spacers and Fluoromount-G mounting medium (Southern Biotech, 0100-01).

### Organoid model and treatments

The human CT29 TSC line was obtained from Riken Biosource Resource Centre and maintained in human TSC culture medium on collagen IV-coated plates according to published methods (Okae et al., 2018). To establish 3D organoids, TSCs were cultured in organoid medium (Turco et al., 2018) at 2×10^6^ cells per 10 ml High Aspect Rotational Vessels (HARVs) on rotational speed 10.8 using the Synthecon Rotary Cell Culture System (RCCS-4D). For product details and the exact composition of the trophoblast stem cell and trophoblast organoid medium components, see Tables S3 and S4. After 18 h of rotation, the cell aggregates were treated with 25 µM ezrin inhibitor (EMD Millipore; cat:341216, lot:3934373) or vehicle control (DMSO). Organoids were collected after 48 h of total culture time for downstream analysis. To collect the organoids, vessels were washed with PBS and spun down at 500 ***g*** for 7 min. Organoids were then processed for immunofluorescent staining or bulk RNA sequencing.

Proliferation was determined via incorporation of EdU into the nuclei using a Click-iT Plus EdU Alexa Fluor 488 Imaging kit (Invitrogen, C10637, lot: 2146725). EdU (10 µM) was added to medium and organoids were incubated for 30 min and fixed according to organoid staining protocols (see below). EdU was detected according to kit instructions.

### Organoid staining

Organoids were fixed in 4% paraformaldehyde for 10 min in 2 ml Sigmacoted (Sigma, SL2) round-bottom tubes on a rocker, then washed three times in PBS. Organoids were permeabilized using 0.5% Triton X-100, then blocked with blocking buffer [5% normal donkey serum, 0.01% Tween-20 and 1:100 human IgG (Invitrogen)]. Organoids were incubated with primary antibodies overnight. For antibody information, please see Table S2. Tissue was then washed with PBS and 0.05% Tween (PBST) and incubated with the fluorescent secondary antibodies AlexaFluor 488 (Invitrogen) or AlexaFluor 594 (Invitrogen) and/or stained with 0.17 µM phalloidin-iFluor 594 (AAT Bioquest, 23122). Organoids were then incubated with 10 µg/ml Hoechst 33342 (Thermo Fisher Scientific, H3570) followed by PBS washes and mounted with imaging spacers and Fluoromount-G mounting medium (Southern Biotech, 0100-01).

### Confocal microscopy image capture and analyses

Triplicate *z*-stack images were acquired with a Zeiss LSM 700 confocal microscope using a Zeiss Plan Apochromat-20×/0.8 M27 or Zeiss Plan Apochromat-63×/1.4 M27 oil lens. *Z*-stack images (15-30 µm) with a 0.6 µm step-size were taken at 63× magnification. *Z*-stack images (60-120 µm) with a 4 µm step-size and single *xy*-plane snapshots were taken at 20× magnification. ST structures were identified as apically located multinucleate structures. vCTs were identified as E-cadherin-positive or ITGA6-positive mononucleate cells.

vCT cell characteristics were measured using 63× *z*-stack confocal microscopy images on placental samples stained with anti-E-cadherin, phalloidin and Hoechst. vCT cell length, vCT volume, vCT shape factor, relative nuclear positioning within vCT, and vCT apical volume were quantified using Volocity Imaging software (Quorum Technologies, version 7.0.0). Shape factor (or sphericity factor) is a numerical value for how similar an object is to a perfect circle or a perfect sphere, as calculated by Volocity. The more circular or spherical an object is, the closer the factor approaches 1. Apical volume was calculated by determining the midline of the cell and then tracing around the apical membrane. A volume for this region of interest (ROI) was calculated by Volocity. Relative positioning of the nucleus was calculated along the apical-basal axis by measuring the length between the centre of the nucleus to the basement membrane and then dividing it by the total length of the cell (Eqn 1):
(1)

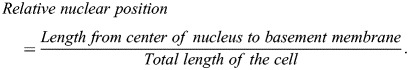
ST coverage and fusion capacity were measured using 20× single-plane confocal microscopy images on placental samples stained with anti-E-cadherin, phalloidin and Hoechst. VCTs were defined as by the presence of a single nuclei bounded by an anti-E-cadherin signal. STs were defined by the presence of multiple nuclei bounded by phalloidin and anti-E-cadherin signal. ST coverage was quantified using Volocity Imaging software by measuring the ratio of the cross-sectional area of the ST compared to the vCT (ST:vCT) (Eqn 2). Donor-normalized ST coverage was additionally calculated by dividing the ST coverage by the ST coverage of donor-matched non-trypsinized control tissue.
(2)

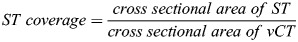
Time-dependent relative change in ST coverage was calculated as the relative ratio of ST:vCT coverage at 72 h compared to 24 h (Eqn 3). Relative fusion in treated explants was normalized to donor-matched vehicle-treated explants.
(3)

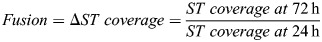
Apical:basal signal (Eqn 4) and apical:total signal (Eqn 5) were determined by the ratio of the signal sums between two cross-sectional regions of interest (ROIs). The apical ROI was defined as the outward facing area of the cell starting above the midline. The basal ROI was defined as the inward facing area of the cell starting below the midline.
(4)

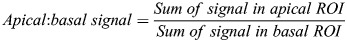

(5)

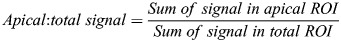


### Scanning electron microscopy (SEM)

At 24 h post-trypsinization, explants were collected, washed in PBS and fixed in EM fixative (2.5% glutaraldehyde, 2% PFA in PBS). Tissue was then washed with PBS and treated with 1% osmium tetroxide in PBS for 1 h. After washing, samples were dehydrated by incubation in a graded ethanol series and then in a graded series of increasing hexamethyldisilazane (HMDS) and decreasing ethanol concentrations. Samples were incubated in 100% HMDS and then air dried overnight. Tissue was then mounted onto standard aluminium stubs and sputter coated with Au/Pd using a Hummer 6.2 Sputter Coater (Anatech). Images were acquired using a Zeiss EVO 10 scanning electron microscope operating at 15 kV and SmartSEM software (Zeiss, version 6.06).

### Transmission electron microscopy (TEM)

PBS washed and trimmed placental tissue was fixed in EM fixative (2.5% glutaraldehyde and 2% PFA in PBS) upon collection. Tissue was then washed with PBS and treated with 1% osmium tetroxide in PBS for 1 h. After washing, tissue was then dehydrated by incubation in a graded ethanol series followed by incubation in 1:1 ethanol:Spurr resin (Electron Microscopy Sciences) overnight. The following day, the tissue was embedded in 100% resin and cured overnight at 70°C. Using a Reichert-Jung Ultracut_E Ultramicrotome, the resin blocks were cut into 70-90 nm sections. Sections were stained with uranyl acetate and lead citrate on 75 mesh grids. Images were acquired using a Philips/FEI (Morgagni) transmission electron microscope operating at 80 kV with Gatan camera and Digital Micrograph software (version, 1.81.78).

### Bright-field imaging

Bright-field images were taken on a Zeiss Cell Discoverer 7 wide-field microscope using an objective Plan-Apochromat 20×/0.7 Autocorr lens (Zeiss).

### RNA isolation and processing

Explant cultured tissue or organoids were collected, rinsed in PBS and then homogenized in TRIzol (Thermo Fisher Scientific, 15596026) using a tissue lyser. RNA from tissue, cultured explants and organoids was extracted using TRIzol-chloroform extraction. RNA was isolated and purified using a PureLink RNA Mini Kit (Ambion, 12183025). Conversion into cDNA was performed using reverse transcription (iScript cDNA Synthesis Kit, 1708890) with 1000 ng RNA.

### Reverse-transcriptase and polymerase chain reaction

RT-PCR was performed using an Applied Biosystems SYBR Green PCR Master Mix (Thermo Fisher Scientific, 4309155) on a QuantStudio 3 Real-Time PCR System (Thermo Fisher Scientific). Primer sequences are indicated in Table S5. Relative change in mRNA expression was calculated using the 2^ΔΔ^CT method to paired samples using the mean CT from *CYC1* and *TOP1* as housekeeping genes (Kaitu'u-Lino et al., 2014; Livak and Schmittgen, 2001; Shaha et al., 2022).

Biological sex of placentas was determined using PCR amplification of the Y-chomosomal *SRY* locus. Samples were amplified using ReadyMix Red Taq PCR (Sigma, R2648, lot SLCF2549) according to the manufacturer's instructions. Primer sequence is indicated in Table S5. Samples were run on a 2% agarose gel at 150 V for 45 min. Afterwards, the gel was stained in SYBR Green I Nucleic Acid Gel Stain (Thermo Fisher Scientific, S7563, SLCD9404) for 30 min, and analysed in an UV imager to observe the presence/absence of bands.

### Bulk RNA-sequencing

Libraries were prepared using the Illumina Stranded mRNA prep kit. Partial lane sequencing was performed by Novogene via Illumina next generation sequencing (NovaSeq X Plus Series PE150). Sequencing data have been deposited in GEO under accession number GSE279087.

Quality control and library alignment of the samples was performed in Ubuntu via the FastQC and STAR distros, respectively (Dobin et al., 2013). We utilized R for the downstream analysis of the data. Counts were obtained utilizing the Rsubread package (Liao et al., 2019). Analysis of the data, estimation of fold changes and dispersion was performed using DESeq2 (Love et al., 2014). The GO pathways were generated using the clusterprofiler package (Wu et al., 2021).

### Statistical analyses

Statistical analyses were conducted using GraphPad Prism (version 9.3.1) with a *P*<0.05 threshold for significance. Exact statistical analyses are provided within the figure legends. Statistical outliers were determined using a ROUT outlier analyses in GraphPad Prism and by PCA analysis for RNA-seq. All graphs and representative images are representative of three to six biological replicates (placental tissue from different donors) and at least three technical replicates.

## Supplementary Material



10.1242/develop.204619_sup1 Supplementary information

Table S1. Full placental sample list with gestational age and associated experiments.

Table S2. Antibody information data table.
