# Supplementary material for: Placental cytotrophoblast microvillar stabilization is required for cell-cell fusion
Source: Development. 2025 Apr 11;152(7):dev204619. doi: 10.1242/dev.204619 (PMC12045602; doi:10.1242/dev.204619)
Supplement: Supplementary information [file develop-152-204619-s1.pdf]

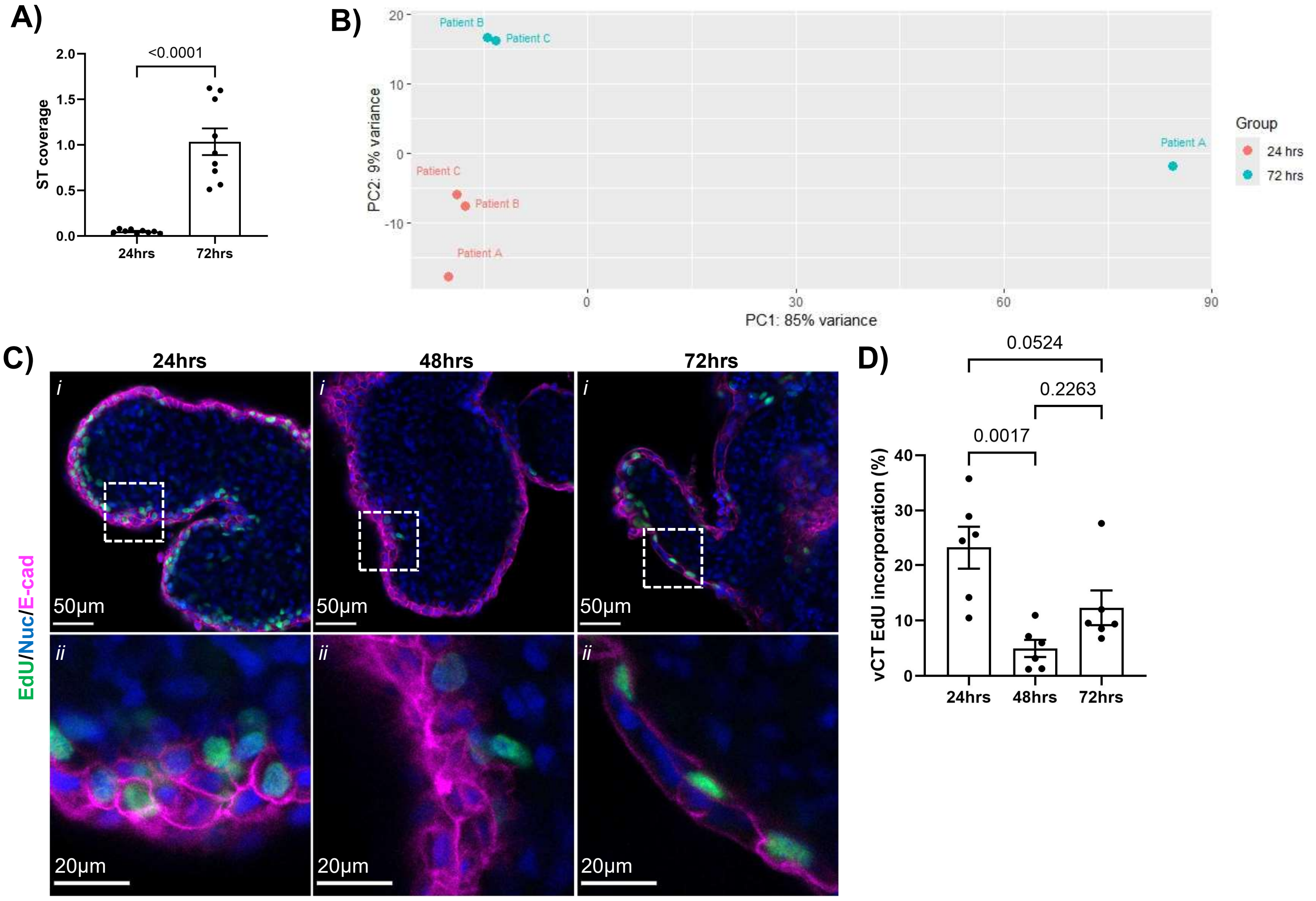

Fig. S1. Explant ST regeneration model characterization. A) Summary data of ST coverage at 24hrs and 72hrs post-trypsinization; mean ± S.E.M., unpaired student's t-test, n=9; B) PCA plot of explants at 24hrs and 72hrs post-trypsinization; PC1 = variance due to sample; PC2 = variance due to culture conditions; C) Representative single XY-plane confocal microscopy images of 10wk GA placental tissue at 24hrs, 48hrs, 72hrs post-trypsinization; *i* = merged images of EdU (green), anti-E-cadherin (magenta), and Hoechst (nuclei; blue); *ii* = higher magnification image of indicated region in *i;* (*i*) scale bar = 50μm; (*ii*) scale bar = 20μm; D) Percent of vCT nuclei incorporated with EdU at 24hrs, 48hrs, and 72hrs post-trypsinization; mean ± S.E.M., unpaired one-way ANOVA with Tukey's multiple comparisons, n=6.

Development • Supplementary information

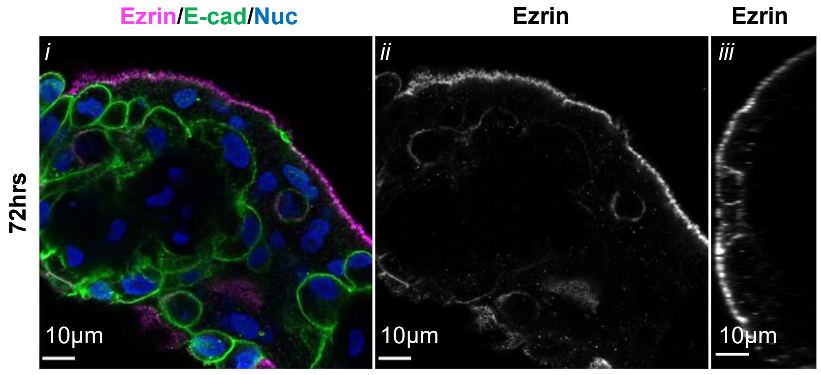

Fig. S2. Ezrin is apically localized in regenerated ST structures. Representative single XY-plane (*i, ii*) and ZY-plane (*iii*) confocal microscopy images of 9.3wk GA tissue 72hrs post-trypsinization; *i* = merged image of anti-ezrin (magenta), anti-E-cadherin (green), and Hoechst (nuclei; blue) signals; *ii* = isolated anti-ezrin (greyscale) signal; *iii* = ZY-plane isolated anti-ezrin (greyscale) signal; scale bar = 10µm.

Development • Supplementary information

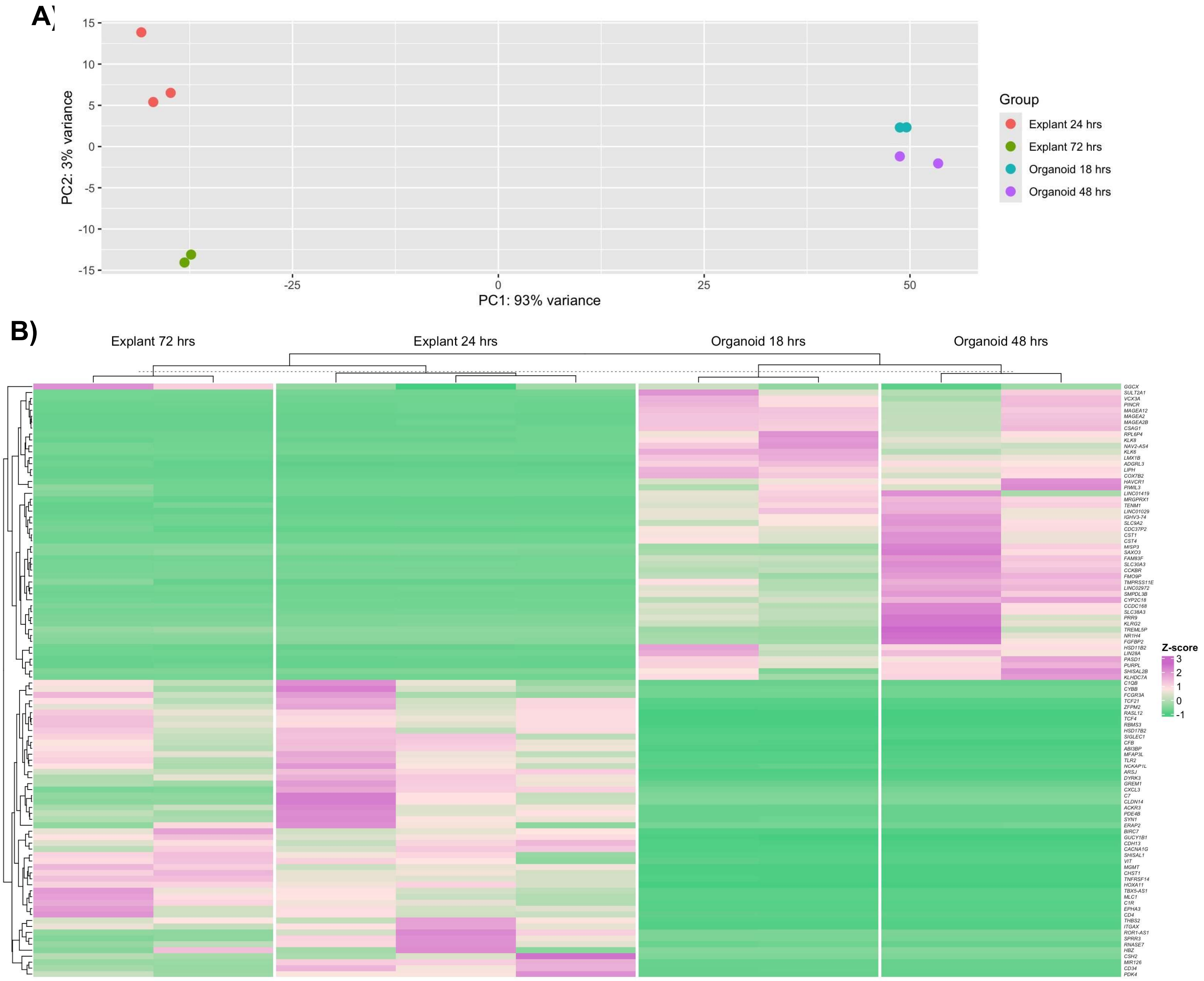

Fig. S3. Transcriptional comparison of TSC organoids and first trimester placental explants. A) PCA plot of explants cultured for 24hrs and 72hrs with organoids cultured for 18hrs and 48hrs; PC1 = variance due to model; PC2 = variance due to time in culture; B) Heatmap plot of the top 50 up-regulated and down-regulated DEG between organoids cultured for 18hrs and 48hrs and explants cultured 24hrs and 72hrs.

Development • Supplementary information

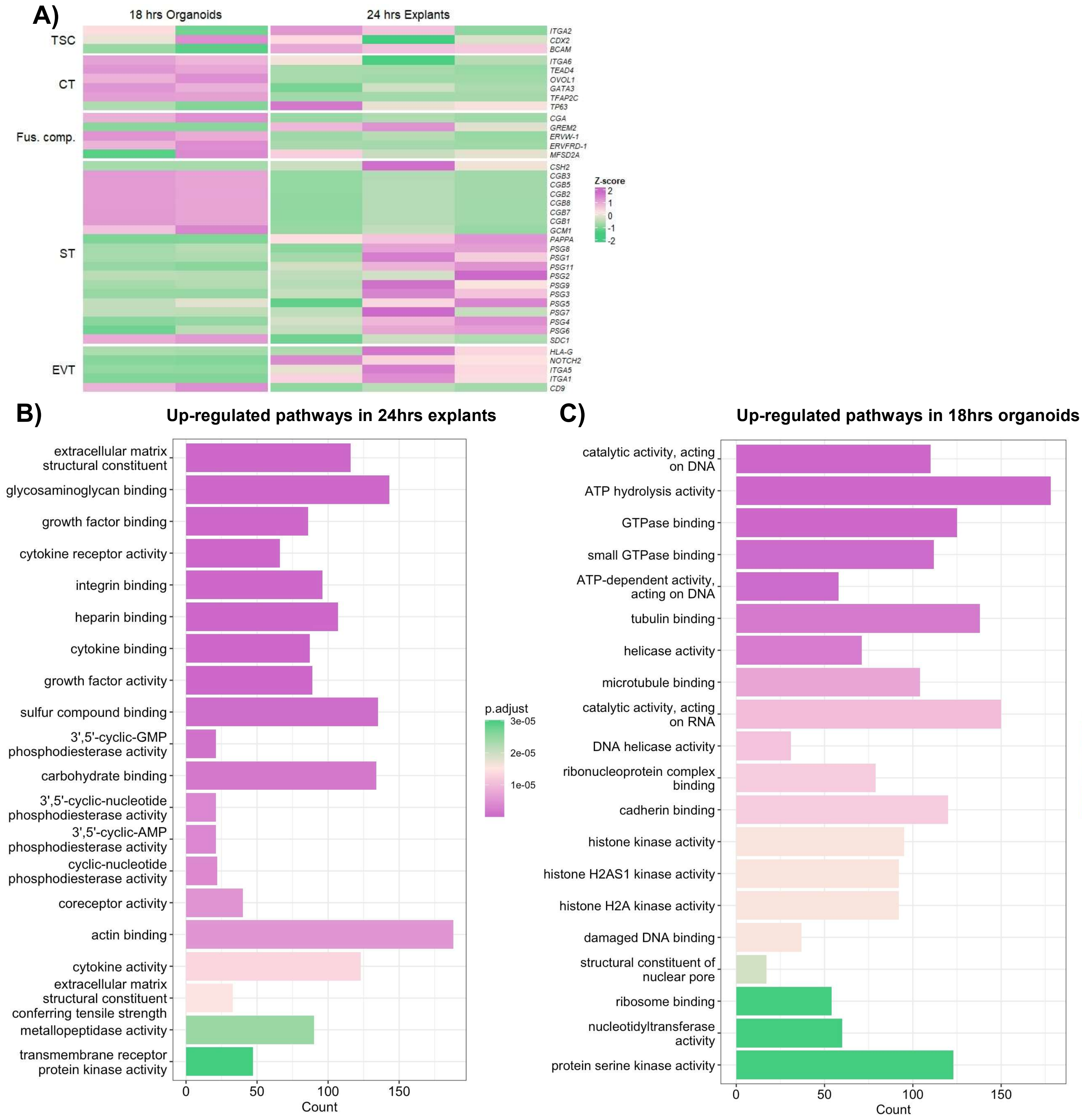

Fig. S4. 18hrs organoids and 24hrs explants are transcriptionally distinct. A) Heatmap plot of organoids cultured for 18hrs vs explants cultured for 24hrs based on z-score of trophoblastic markers; B) GO pathway analyses on enriched biological processes in explants at 24hrs compared to organoids at 18hrs; C) GO pathway analyses on enriched biological processes in organoids at 18hrs compared to explants at 24hrs; all GO pathways were produced using the top 50 DEG.

Development • Supplementary information

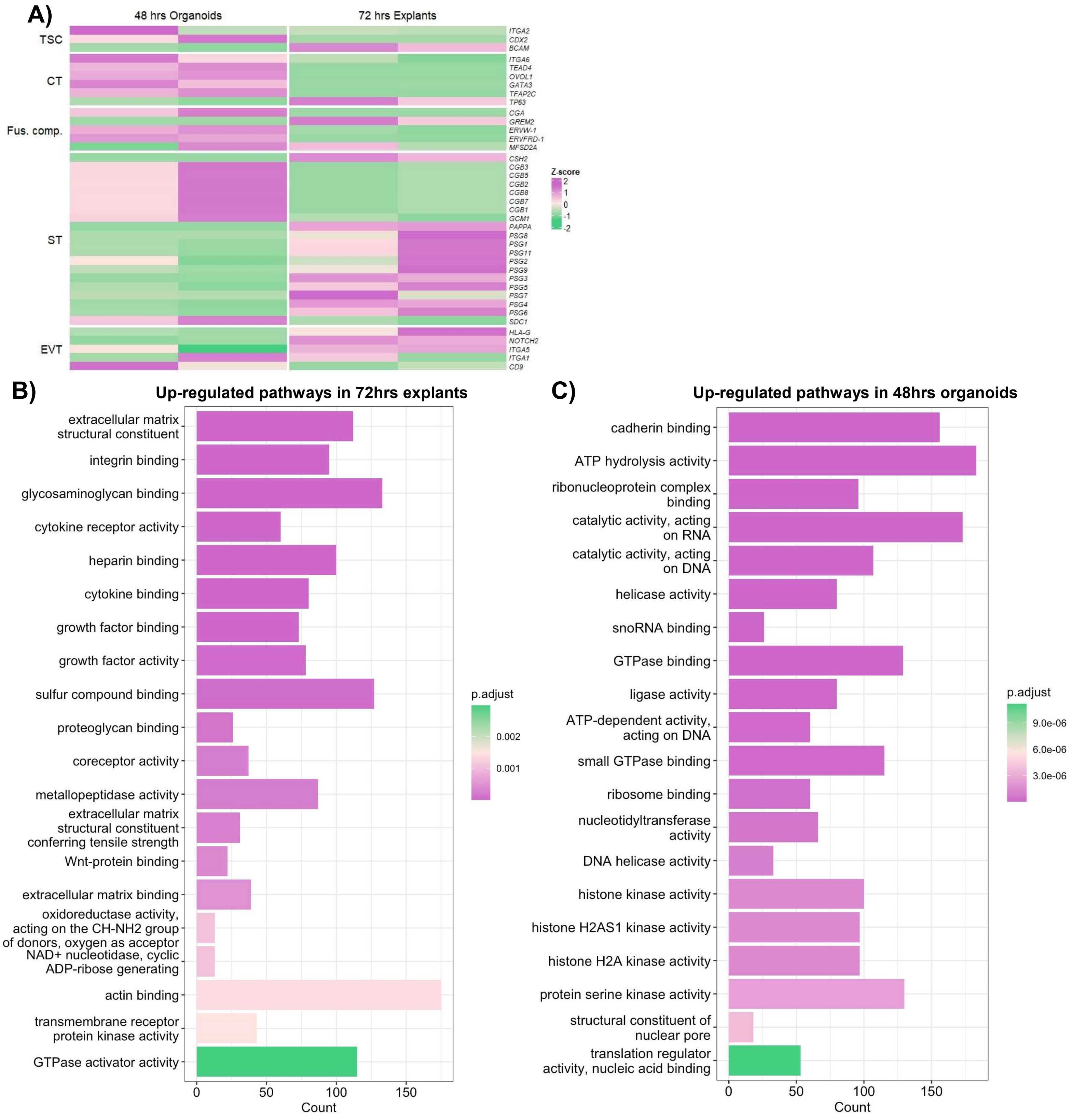

Fig. S5. 48hrs organoids and 72hrs explants are transcriptionally distinct. A) Heatmap plot of organoids cultured for 48hrs vs explants cultured for 72hrs based on z-score of trophoblastic markers; B) GO pathway analyses on enriched biological processes in explants at 72hrs compared to organoids at 48hrs; C) GO pathway analyses on enriched biological processes in organoids at 48hrs compared to explants at 72hrs; all GO pathways were produced using the top 50 DEG.

Development • Supplementary information

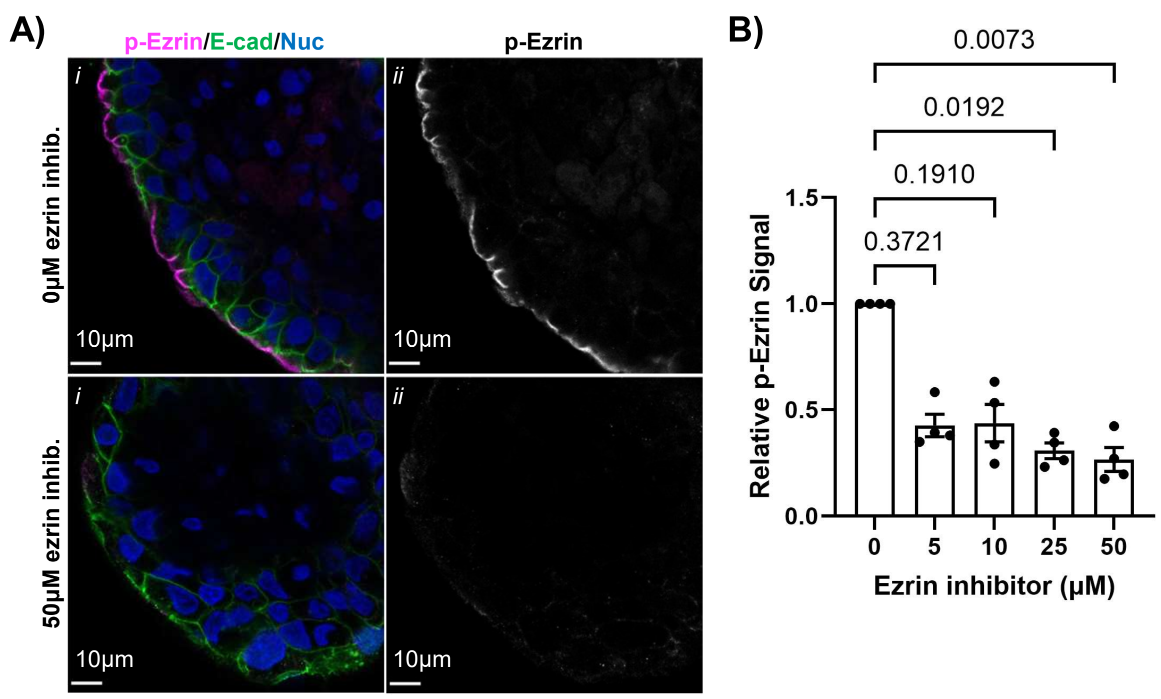

Fig. S6. Ezrin inhibitor treatment decreases active ezrin signal in vCTs. A) Representative single XY-plane confocal microscopy images of 10wk GA explants pulsed for 2hrs ± 50μM ezrin inhibitor at 24hrs post-trypsinization; *i* = merged image of anti-phospho-Thr567-ezrin (magenta), anti-E-cadherin (green), and Hoechst (nuclei; blue) signals; *ii* = isolated anti-phospho-Thr567-ezrin (greyscale) signal; scale bar = 10μm; B) Summary data of dose-dependent ezrin inhibitor treatment on anti-phospho-Thr567-ezrin signal, normalized to vehicle control; mean ± S.E.M., unpaired Kruskal-Wallis test with Dunnett's multiple comparisons, *n*=4.

Development • Supplementary information

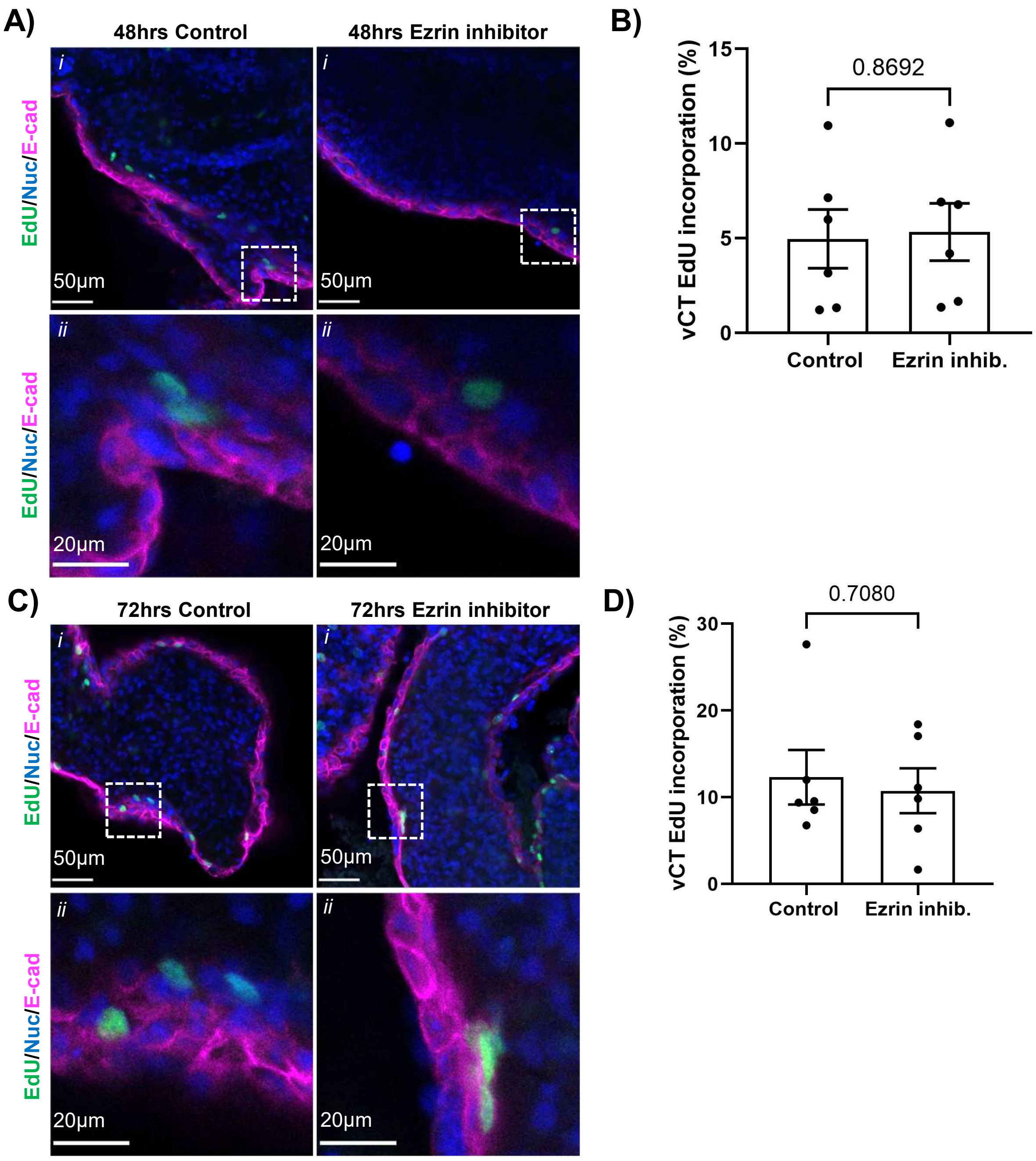

Fig. S7. Ezrin inhibition does not affect vCT proliferation in explants. A) Representative single XY-plane confocal microscopy images of 10wk GA placental explants cultured for 48hrs total ± 50µM ezrin inhibitor added 24hrs post-trypsinization; *i* = merged images of EdU (green), anti-E-cadherin (magenta), and Hoechst (nuclei; blue); *ii* = higher magnification image of indicated region in *i*; (*i*) scale bar = 50µm; (*ii*) scale bar = 20µm; B) Percent of vCT nuclei incorporated with EdU in explants cultured for 48hrs total ± 50µM ezrin inhibitor added 24hrs post-trypsinization; mean ± S.E.M., unpaired student's t-test, *n*=6; C) Representative single XY-plane confocal microscopy images of 10wk GA placental explants cultured for 72hrs total ± 50µM ezrin inhibitor added 24hrs post-trypsinization; *i* = merged images of EdU (green), anti-E-cadherin (magenta), and Hoechst (nuclei; blue); *ii* = higher magnification image of indicated region in *i*; (*i*) scale bar = 50µm; (*ii*) scale bar = 20µm; D) Percent of vCT nuclei incorporated with EdU in explants cultured for 72hrs total ± 50µM ezrin inhibitor added 24hrs post-trypsinization; mean ± S.E.M., unpaired student's t-test, *n*=6.

Development • Supplementary information

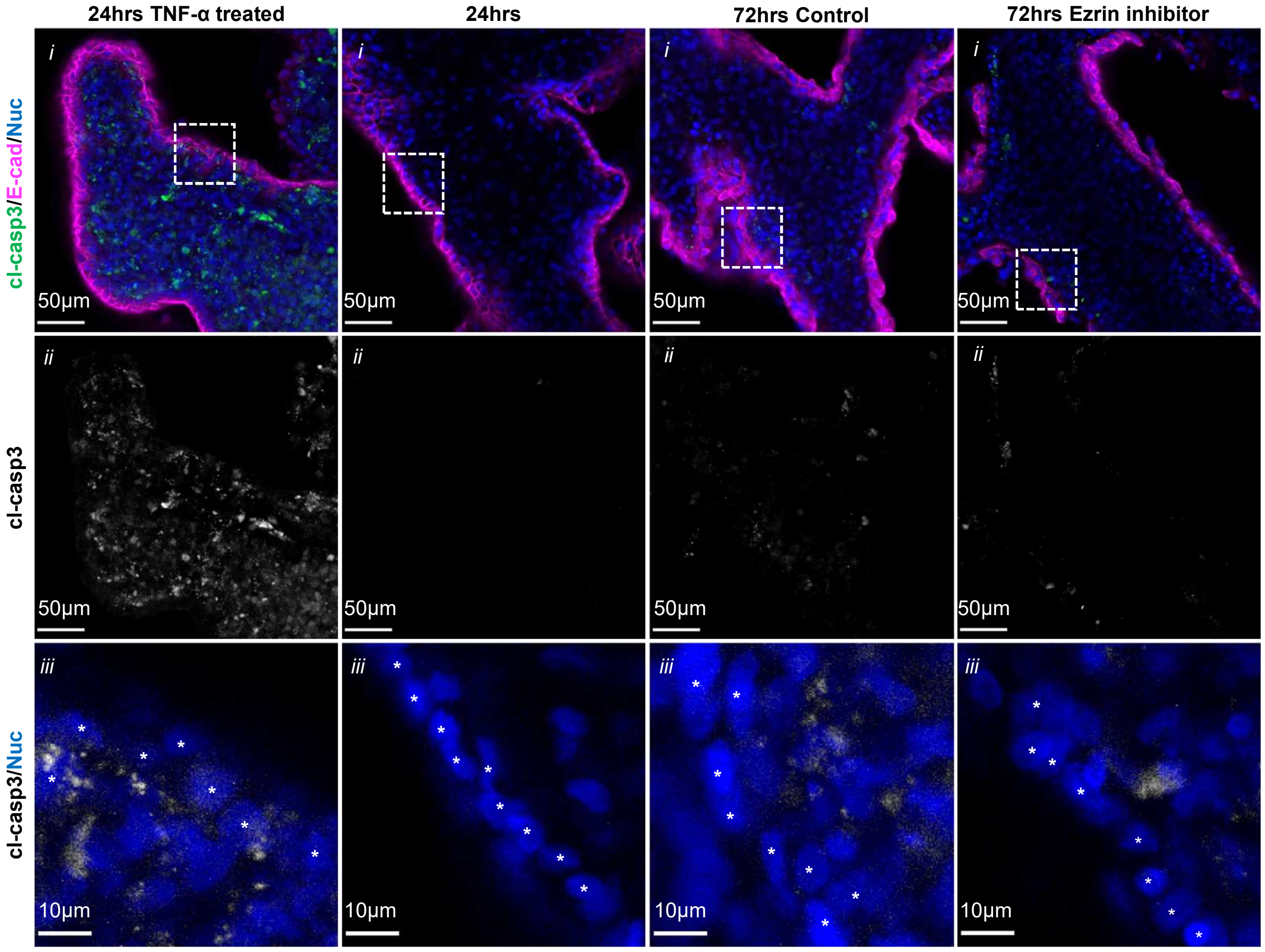

Fig. S8. Ezrin inhibition does not affect vCT viability in explants. Representative single XY-plane confocal microscopy images of 10.5wk GA placental tissue cultured ± 100pg/mL TNF-α and cultured for 72hrs total ± 50µM ezrin inhibitor; *i* = merged images of anti-cleaved-caspase-3 (green), anti-E-cadherin (magenta), and Hoechst (nuclei; blue); *ii* = isolated anti-cleaved-caspase-3 (greyscale) signals; *iii* = higher magnification image of indicated region in *i* with isolated anti-cleaved-caspase-3 (greyscale) and Hoechst (nuclei; blue) signals; asterisks indicate vCT nuclei; (*i, ii*) scale bar = 50µm; (*iii*) scale bar = 10µm.

Development • Supplementary information

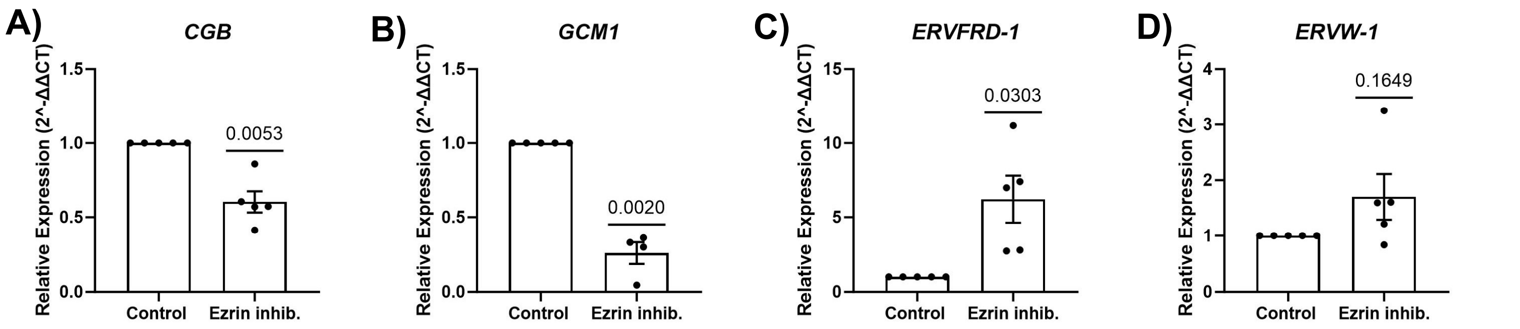

Fig. S9. Ezrin inhibition alters gene transcription as quantified via RT-PCR in explants. Relative A) *CGB* B) *GCM1* C) *ERVFRD-1* and D) *ERVW-1* expression in explants cultured for 48hrs total ± 50µM ezrin inhibitor added 24hrs post-trypsinization; Data represented as $2^{-\Delta\Delta}$CT to mean of *TOP1* and *CYC1* normalized to controls; mean ± S.E.M., one-sample t-test, n=4-5.

Development • Supplementary information

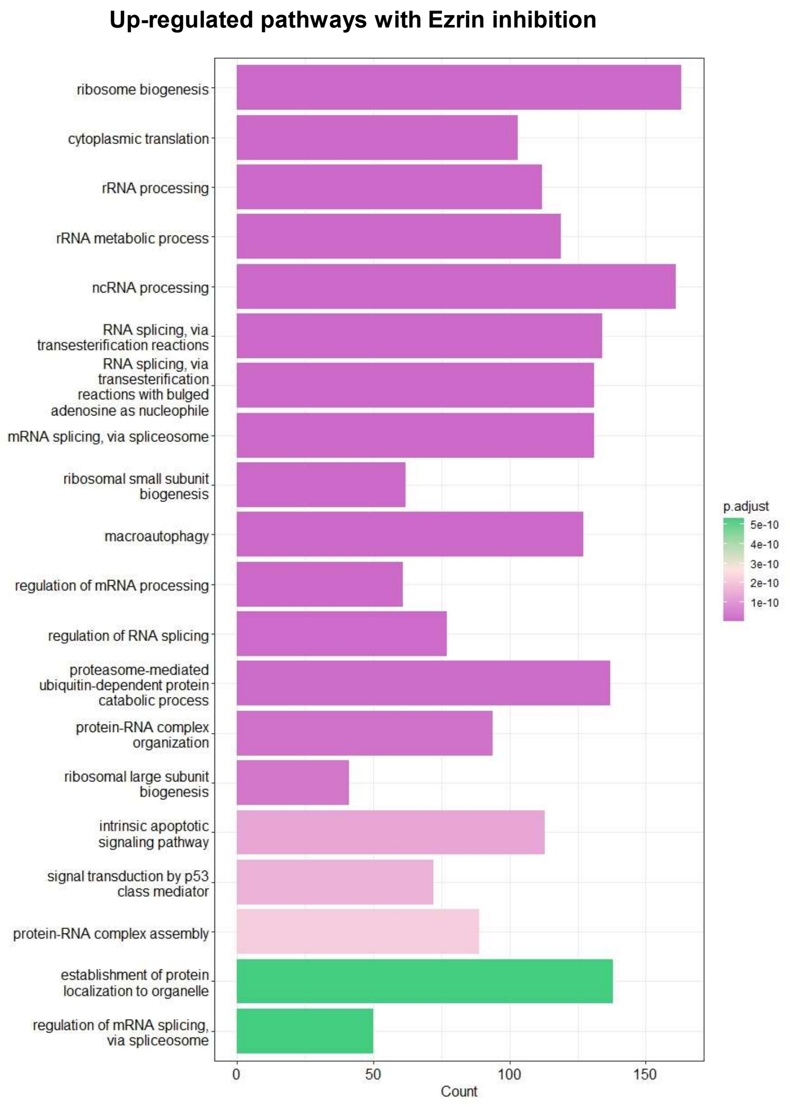

Fig. S10. Ribosome biogenesis, RNA splicing, and protein-RNA complex regulation is up-regulated in explants with ezrin inhibition. GO pathway analyses on upregulated biological processes in explants at 48hrs ± 50μM ezrin inhibitor; all DEG analyzed had a p-adjusted value of <0.05 and a log2 fold change of below -0.5.

Development • Supplementary information

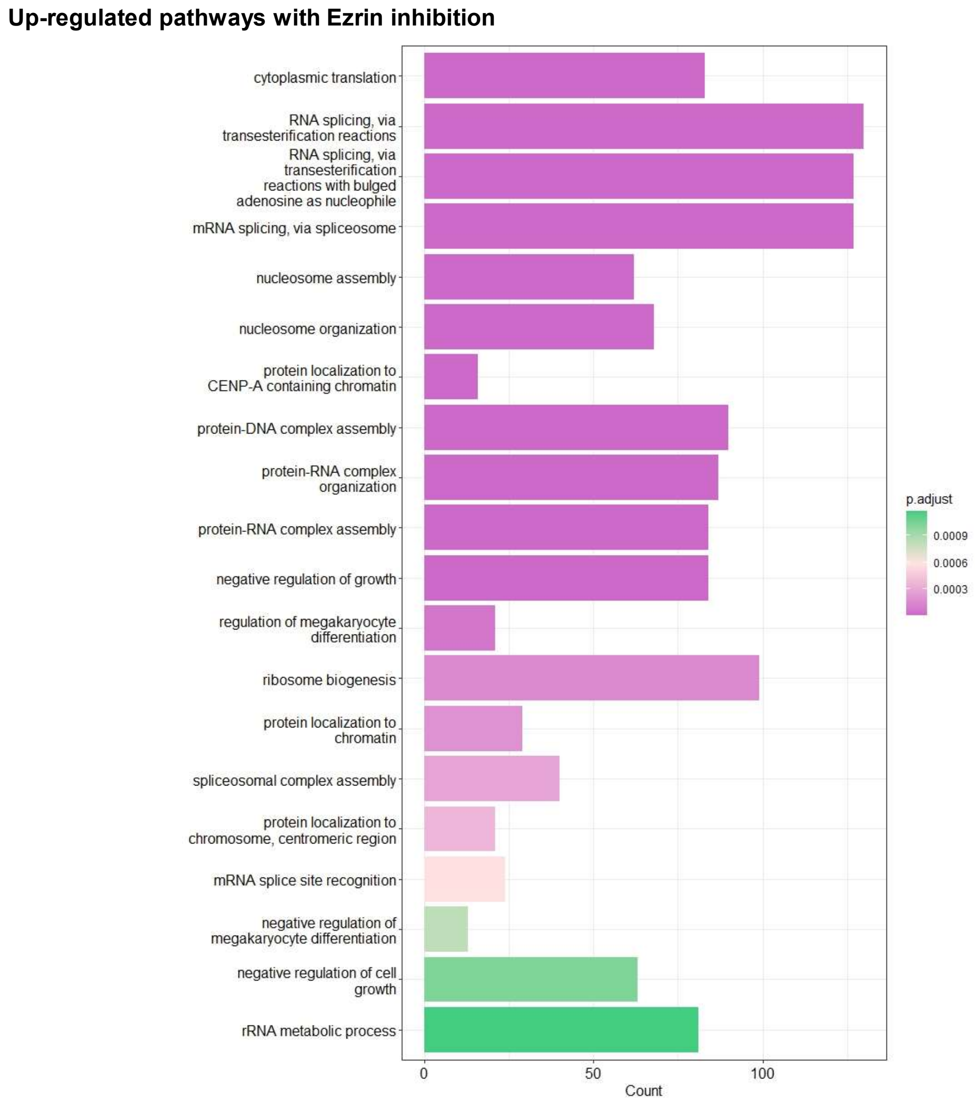

Fig. S11. Ribosome biogenesis, RNA splicing, and protein-RNA complex regulation is up-regulated in organoids with ezrin inhibition. GO pathway analyses on upregulated biological processes of organoids at 48hrs ± 25μM ezrin inhibitor; all DEG analyzed had a p-adjusted value of <0.05 and a log2 fold change of below -0.5.

Table S1. Full placental sample list with gestational age and associated experiments.

Available for download at
https://journals.biologists.com/dev/article-lookup/doi/10.1242/dev.204619#supplementary-data

Table S2. Antibody information data table.

Available for download at
https://journals.biologists.com/dev/article-lookup/doi/10.1242/dev.204619#supplementary-data

Development • Supplementary information

Table S3. Trophoblast medium component data table.

Component	Final concentration	Product Information (catalogue #)
DMEM/F12		Gibco (11320-033)
BSA	0.3%	Sigma (A9085)
ITS-X	1X	Gibco (51500056)
Primocin	100ug/mL	Invivogen (ant-pm-1)
2-Mercaptoethanol	0.1mM	Sigma (M3148)
Human EGF	50ng/ml	Peprotech (AF-100-15)
FBS	0.2%	Wisent (98150)
L-ascorbic acid	1.5µg/ml	Sigma (A5960)
CHIR 99021	2µM	Biogems (2520691)
Y-27632	5µM	Biogems (1293823)
A 83-01	0.5µM	Biogems (9094360)
SB431542	1µM	Sigma (616464)
Valproic acid sodium salt	0.8mM	Biogems (1066656)
Mouse Collagen IV	5ug/mL coating for plates	Corning (CB-40233)

Table S4. Organoid medium component data table.

Component	Final concentration	Product Information (catalogue #)
Advanced DMEM/F12		Gibco (cat: 12634-101)
N-2 Supplement	1X	Gibco (cat: 17502048)
B-27 supplement, minus vitamin A	1X	Gibco (cat: 12587010)
Primocin	100ng/mL	InvivoGen (cat: ant-pm-1)
L-Glutamine	2mM	Gibco (cat: 21051-024)
Human R-Spondin-1 Recombinant Protein	80ng/mL	Peprotech (cat: 120-38)
Human EGF, Animal-Free Recombinant Protein	50ng/mL	Peprotech (cat: AF-100-15)
Prostaglandin E2	2.5μM	BioGems (cat: 3632464)
CHIR 99021	1.5μM	BioGems (cat: 2520691)
Human HGF Recombinant Protein	50ng/mL	Peprotech (cat: 100-39)
Y-27632 Dihydrochloride	2μM	BioGems (cat: 1293823)
A 83-01	500nM	BioGems (cat: 9094360)
Human FGF-basic (FGF-2/bFGF) Recombinant Protein	100ng/mL	Peprotech (cat: 100-18C)
N-Acetyl-L-cysteine	1.25 mM	Sigma (cat: A9165)

Table S5. RT-PCR primer information data table.

Gene name	Forward primer (5' to 3')	Reverse primer (5' to 3')
CGB	GCCTCATCCTTGGCGCTAGA	TATACCTCGGGGTTGTGGGG
CYC1	CAGATAGCCAAGGATGTGTG	CATCATCAACATCTTGAGCC
ERVFRD-1	CCAAATTCCCTCCTCTCCTC	CGGGTGTTAGTTTGCTTGGT
ERVW-1	GTCACTGTCTGTTGGACTTACT	CGGCTGAGTTGGGAGATTAC
GCM1	GTGCTGTCTGCTTCTCCGTA	GATAAGGTCAGGCCAGCCAA
SRY	CAGATCCCGCTTCGGTACTC	TTTGTCCAGTGGCTGTAGCG
TOP1	GATGAACCTGAAGATGATGGC	TCAGCATCATCCTCATCTCG

Development • Supplementary information

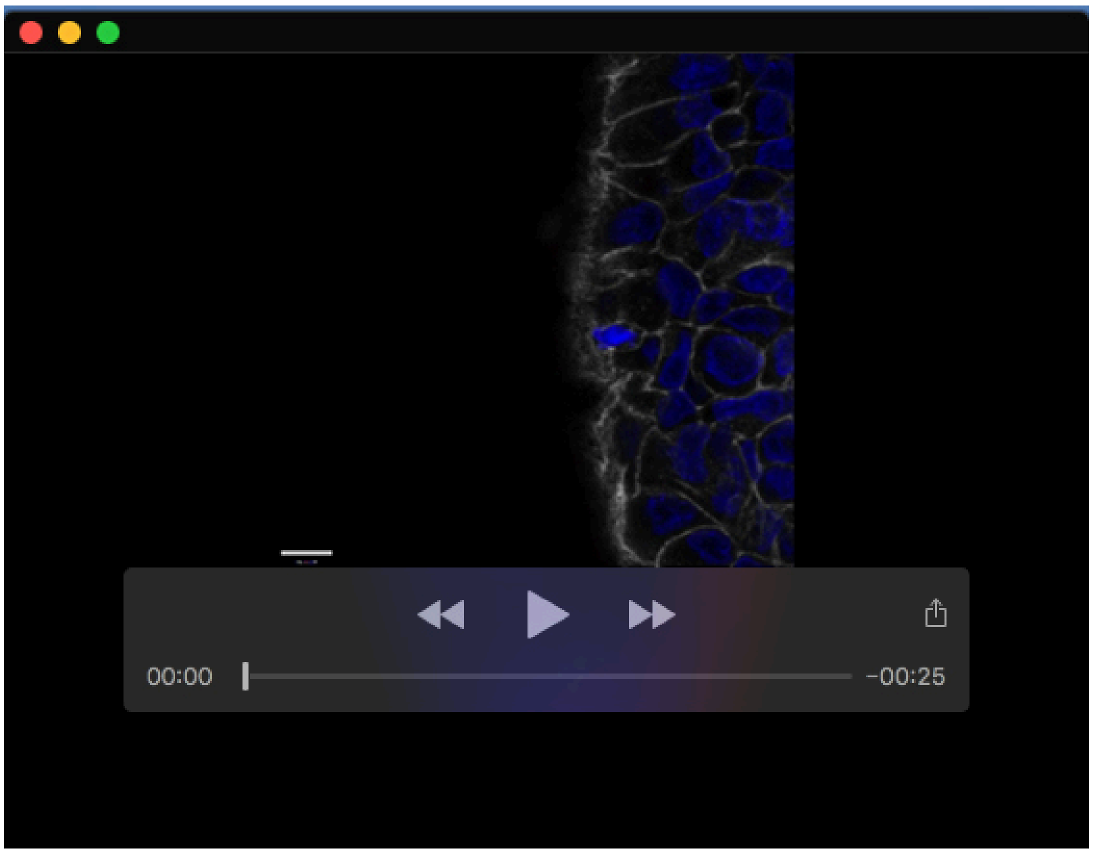

Movie 1. XY-plane confocal microscopy movie of 12wk GA placental tissue 24hrs post-trypsinization; merged phalloidin (greyscale) and Hoechst (nuclei; blue) signals; scale bar = 10μm.

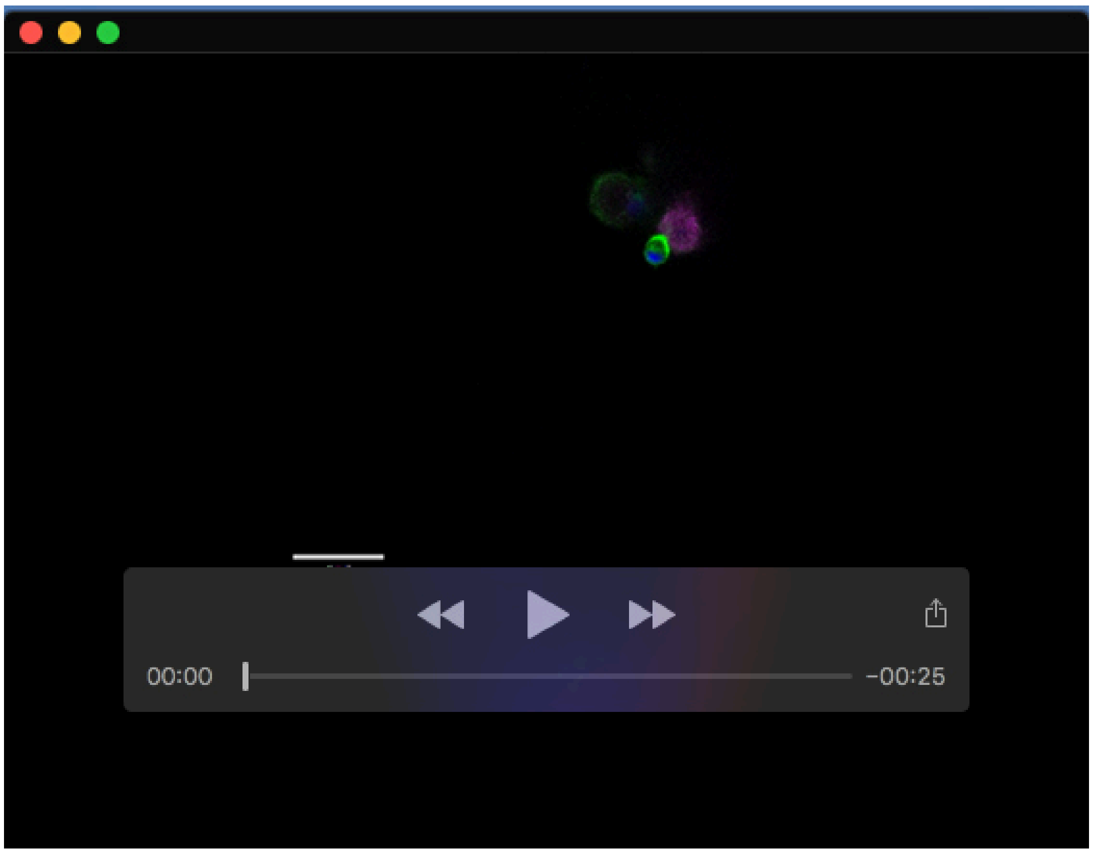

Movie 2. XY-plane confocal microscopy movie of organoids cultured for 48hrs; merged anti-β-hCG (magenta), anti-ITGA6 (green), Hoechst (nuclei; blue) signals; scale bar = 50μm.

Development • Supplementary information

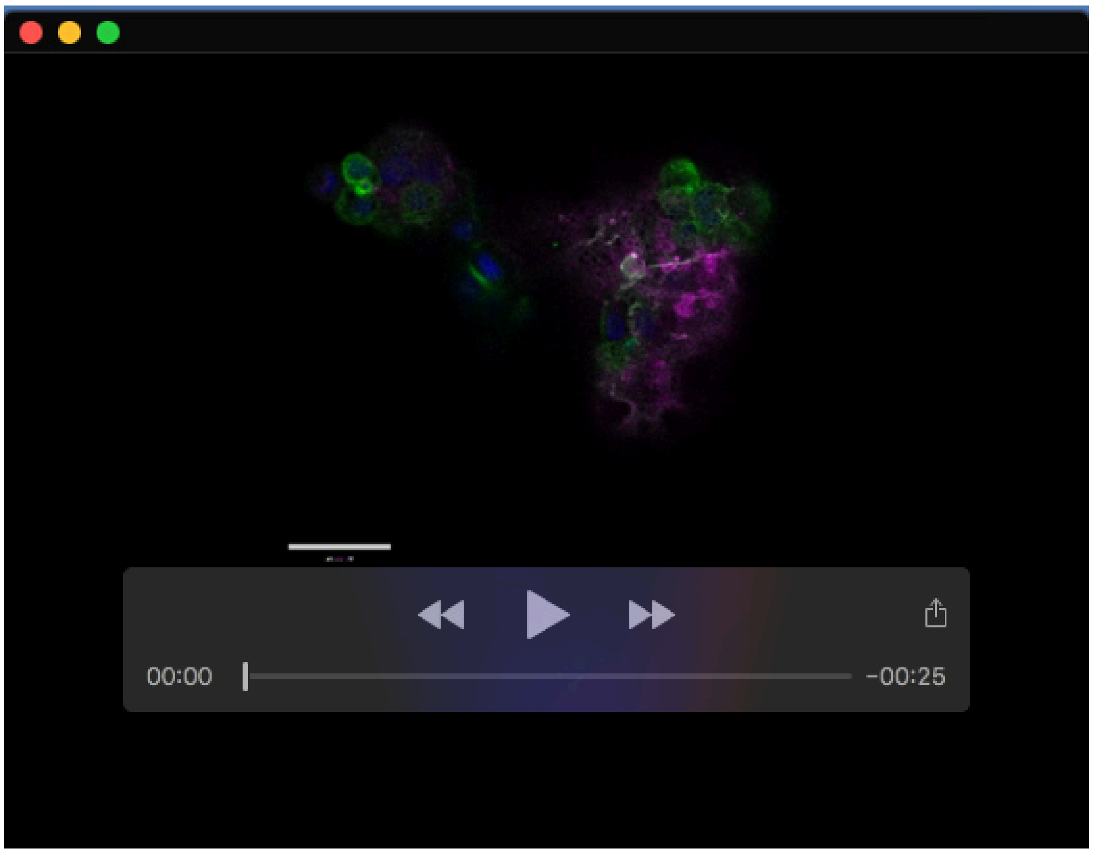

Movie 3. XY-plane confocal microscopy movie of organoids cultured for 48hrs; merged anti-SDC1 (magenta), anti-ITGA6 (green), Hoechst (nuclei; blue) signals; scale bar =50μm.

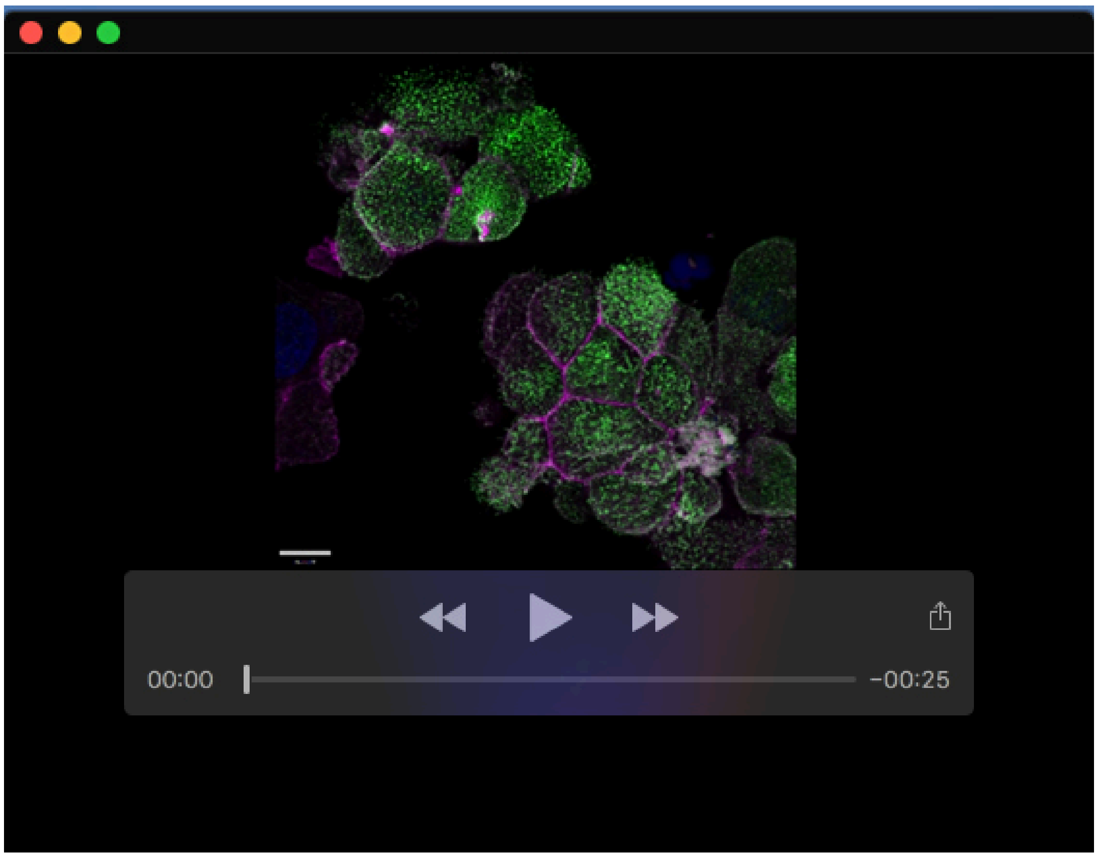

Movie 4. XY-plane confocal microscopy movie of organoids cultured for 18hrs; merged phalloidin (magenta), anti-ezrin (green), and Hoechst (nuclei; blue) signals; scale bar = 10μm.

Development • Supplementary information

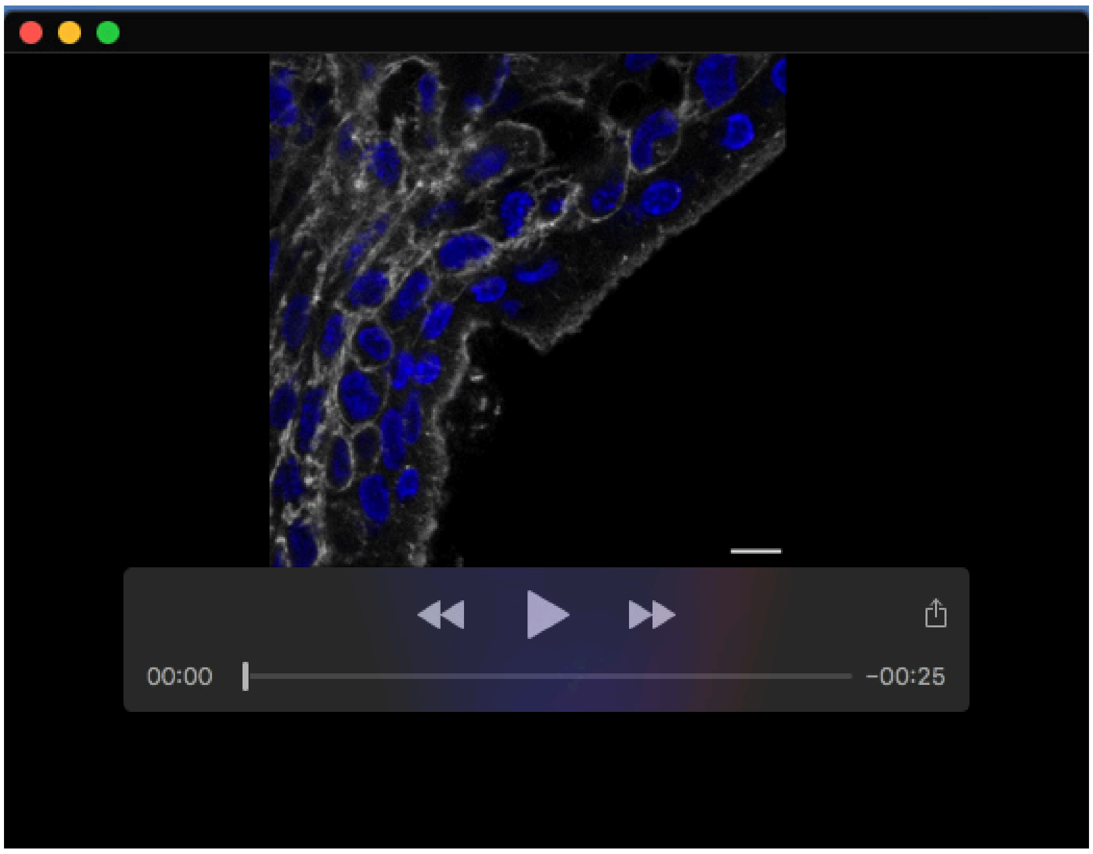

Movie 5. XY-plane confocal microscopy movie of uncultured 10wk GA placental tissue along the villous length; merged phalloidin (greyscale) and Hoechst (nuclei; blue) signals; scale bar = 10μm.

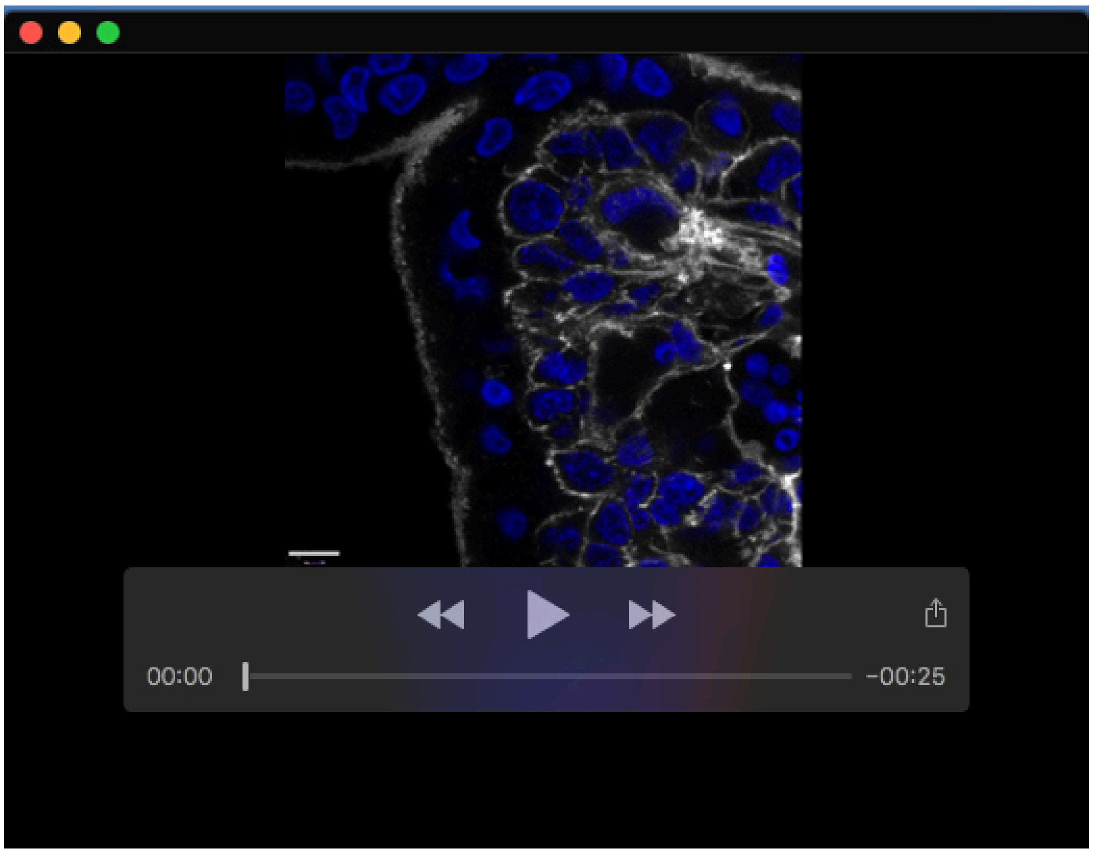

Movie 6. XY-plane confocal microscopy movie of uncultured 10wk GA placental tissue at the villous tip; merged phalloidin (greyscale) and Hoechst (nuclei; blue) signals; scale bar = 10μm.

Development • Supplementary information
