# Supplementary material for: Placental cytotrophoblast microvillar stabilization is required for cell-cell fusion
Source: Development. 2025 Apr 11;152(7):dev204619. doi: 10.1242/dev.204619 (PMC12045602; doi:10.1242/dev.204619)
Supplement: Supplementary file 1 [file develop-152-204619_review_history.pdf]

Development

Placental cytotrophoblast microvillar stabilization is required for cell-cell fusion

Wendy K. Duan, Sumaiyah Z. Shaha, Juan F. Garcia Rivas, Bethan L. Wilson, Khushali J. Patel, Ivan K. Domingo and Meghan Riddell

DOI: 10.1242/dev.204619

Editor: Peter Rugg-Gunn

Review timeline

Original submission:	22 February 2024
Editorial decision:	15 April 2024
First revision received:	15 October 2024
Editorial decision:	26 November 2024
Second revision received:	14 January 2025
Accepted:	3 March 2025

Original submission

First decision letter

MS ID#: DEVELOP/2024/202826

MS TITLE:Placental cytotrophoblast microvillar stabilization is required for cell-cell fusion

AUTHORS:Wendy K. Duan, Sumaiyah Z. Shaha, Khushali J. Patel, Ivan K. Domingo, and Meghan Riddell

I have now received all the referees' reports on the above manuscript, and have reached a decision. The referees' comments are appended below, or you can access them online: please go to BenchPress and click on the 'Manuscripts with Decisions' queue in the Author Area.

As you will see, the referees express considerable interest in your work, but have some significant criticisms and recommend a substantial revision of your manuscript before we can consider publication. If you are able to revise the manuscript along the lines suggested, which may involve further experiments, I will be happy receive a revised version of the manuscript. Your revised paper will be re-reviewed by one or more of the original referees, and acceptance of your manuscript will depend on your addressing satisfactorily the reviewers' major concerns. Please also note that Development will normally permit only one round of major revision. If it would be helpful, you are welcome to contact us to discuss your revision in greater detail. Please send us a point-by-point response indicating your plans for addressing the referees' comments, and we will look over this and provide further guidance.

Please attend to all of the reviewers' comments and ensure that you clearly highlight all changes made in the revised manuscript. Please avoid using 'Tracked changes' in Word files as these are lost in PDF conversion. I should be grateful if you would also provide a point-by-point response detailing how you have dealt with the points raised by the reviewers in the 'Response to Reviewers' box. If you do not agree with any of their criticisms or suggestions please explain clearly why this is so.

Reviewer 1 Advance Summary and Potential Significance to Field:

© 2025. Published by The Company of Biologists under the terms of the Creative Commons Attribution License (https://creativecommons.org/licenses/by/4.0/).

Using a human placental explant model, the authors characterized the morphology of fusion-competent cytotrophoblasts were changed , that form microvilli at the apical membrane. The actin-membrane cross linker protein ezrin within cytotrophoblast microvilli was important to cytotrophoblast fusion. Inhibition of ezrin activation destabilized cytotrophoblast microvilli and prevented cytotrophoblast fusion.

Reviewer 1 Comments for the Author:
The following comments may help to better illustrate the project.
1、Fig1A. The picture is not clear, please improve the quality of the image.
2、Fig1A. The staining of E-cad can only see monolayers of cells, which does not indicate cell identity, and it is also recommended that the multinucleated areas on the graph be zoomed.
3、Fig2B. The content of the picture lacks specific instructions, please add labels to the picture
4、Whether ezrin changes in the placental villi at different periods of time
5、Fig3B. Please indicate the period of placental villi on the image
6、Page 9, line 191, : Please explain why extensive microvilli were not clearly visible in the majority of vCT.
7、Page 11, line 241, The data results of a significant increase in relative ERVFRD-1 expression and a non-significant increase in ERVW-1do not directly lead to the conclusions ofblocking ezrin activation stalled vCT differentiation into ST. Please adjust the language logic.
8、The use of NSC668394 to inhibit ezrin and study its effect on cytotrophoblast fusion is innovative. However, a rescue experiment using an ezrin variant that is resistant to NSC668394 inhibition could provide compelling evidence for the specificity of the observed effects.

Reviewer 2 Advance Summary and Potential Significance to Field:
The manuscript by Duan et al uses a first trimester placental villi floating system to study actin stabilization, localization, and the importance of the actin-scaffold protein, ezrin, in villous cytotrophoblast (vCT) cell-cell fusion. The explant system is an excellent 3D approach that retains ex vivo morphology and cell-cell communication, aspects that are critical for understanding syncytiotophoblast (ST) development and maintenance.

Using the explant system, the authors show that following ST removal using a trypsin digest, vCT morphology is altered, and filamentous actin protrusions are formed pre ST. The actin scaffold protein ezrin was shown to localize to the apical surface of vCT and further localized to actin extensions. Inhibition of ezrin's ability to bind to actin polymers essentially blocked ST formation, showing that ezrin is essential in ST development. Parts of these findings are novel, while other findings (i.e., inhibition of ezrin on ST formation) are not entirely novel.

The work presented in this manuscript is overall high quality. The images are excellent, and the fundamental experimental design is appropriate. Additionally, the manuscript is well written and easy to follow.

However, the design does have inherent limitations (in detail below), and the conceptual advancement of this work (over previous work showing the requirement of ezrin in trophoblast cell-cell fusion) should be addressed more clearly and carefully. In the manuscript's current state, I feel the findings are overly descriptive, and while the findings do shed some novel insight into ezrin stabilization of actin protrusions important in ST formation, the conceptual and mechanistic advancements are currently limited.

Reviewer 2 Comments for the Author:
Specific comments:

1)More clarity on the clinical samples obtained would strengthen the manuscript. How many placentas were collected? Was biological sex considered? What was the breakdown of gestational age in the samples collected? This data could be tabulated, and individual samples could also be indicated to specific
experiments performed.

© 2025. Published by The Company of Biologists under the terms of the Creative Commons Attribution License (https://creativecommons.org/licenses/by/4.0/).

2)A central concern this reviewer has relates to the possible unintended effect that trypsin has on cell morphology and actin dynamics that is independent of pre ST/fusion vCT biology. This concern could underlie a lot of the study's findings. Trypsin may also lead to stress-induced protein-complex distributions that are typically not occurring in non-trypsin stressed cells. Would subjecting TSCs to a fusion assay as an alternative way to measure cell-cell fusion further support and strengthen the study's findings?

https://doi.org/10.1016/0014-4827(78)90029-0
https://doi.org/10.1016/j.ymeth.2013.02.014

3)There is also a concern about the effect of higher concentrations of NSC668394 impacting cell viability and proliferation (independent of the effects of this inhibitor on ezrin threonine phosphorylation). Can the authors show that the increasing doses of NSC668394 in SCT denuded explants is not inhibiting SCT formation in part due to impaired vCT viability?

4)Related to the above comment, while the authors show convincing evidence using the ezrin inhibitor that ezrin-mediated actin interactions and apical polarity (and perhaps ezrin's other function as an AKAP that influences PKA localization) are important for ST development, the concern that the inhibitor may be contributing to suboptimal cell viability is a limitation of this approach. Are there other complementary ways to inhibit ezrin that would ultimately support the study's findings? Is knock-down feasible in explants? If not, would this not justify the use of a non-explant model that is amenable to ezrin knock-down to verify these findings?

5)SCT coverage: In the methods line 420, the authors indicate that Ecad, phalloidin, and Hoechst stains were used to facilitate the quantification of SCT coverage. Indeed the equation for this was shown. However, what defining parts of this stain helped to identify the ST compared to the vCT? Also, in areas where vCT are predicated to have been exhausted (i.e., not present), how does this affect the ST coverage calculation?

6)The authors, through the execution of excellent imaging analyses, describe the morphology of vCT that are stated as pre-fusing. How do the authors know these are pre-fusing vCT? If this can't be done, perhaps a tempering of the language that describes these morphological changes may be needed.

7)Extensive microvilli versus podosomes; I am not sure that the apical structure of ezrin positive microvilli is sufficient to distinguish a microvilli from a podosome. Have the authors co-stained with podosome markers to confirm this?

8)What is the purpose or role of stabilized microvilli protrusions in ST formation? They seem to be required for cell fusion, but this study does not really provide insight into how these structures are contributing to cell fusion. Are these features providing a route for specific fusogenic proteins or other cargo like vacuoles? The relationship between ezrin and PKA is perhaps something worth examining as well as it is unclear to this reviewer what ezrin is doing: 1) controlling actin dynamics/stabilization; 2) functioning as an AKAP and modulating PKA localization and function. This comment relates to the overall impact of the paper and the conceptual advance the paper may/may not have.

9)Line 120: The authors provide a rationale for solely using the floating villous explant system to study cell fusion. The authors indicate that the explant system provides the advantage of a 3D system where progenitor vCT reside on top of a basement membrane, and where cell-cell fusion into SCT occurs spontaneously. These features are stated to not occur in 2D TSC cultures, current organoid systems, and 2D primary CTB cultures. I would argue that this interpretation is not 100% accurate. Many cells lay down matrix, including basement membrane-associated proteins like laminin in 2D culture; trophoblast organoids, while differentiating into SCT in an inverse orientation, nonetheless have a laminin-rich basement membrane for vCT like cells to situate on, and similar to primary vCT and floating explants models, cell fusion is also spontaneous. These nuances are important as there are also limitations to the explant system (exposure to trypsin, villous viability, lack of progenitor renewal, difficulties in mechanistic interrogation) that should be also considered and limit the overall scope of the study and the interpretation of the study's

© 2025. Published by The Company of Biologists under the terms of the Creative Commons Attribution License (https://creativecommons.org/licenses/by/4.0/).

findings. I would argue that applying the explant system in conjunction with another supporting cell-based system would provide important redundancy and improve overall reliability/robustness of the data.

10)Data shown in Figure 1A. Trypsin-mediated removal of the outer syncytial layer shows that it was effective. However, following 72 hrs, it is not entirely clear to this reviewer that a SCT layer spontaneously reformed. The image is not magnified enough to appreciate distinctions between vCT and newly-formed syncytiotrophoblast. Further, it appears that the arrows are pointing to Ecad positive cells; are not multinucleated SCT Ecad low or null relativeto vCT? The Cd49f signal is likewise hard to discern between vCT and new SCT. It is clear that hCG is robustly present by 72 hrs, but the magnification/clarity of the image make it hard to visualize a distinct SCT layer.

Minor:
Line 125: Sentence suggests that syncytial removal required 24 hrs of trypsin exposure. I don't think this is the case.

Line 329: The authors statement that in trophoblast organoids the matrix is surrounding a "ball" of progenitor trophoblasts is somewhat inaccurate. While the overall shape is ball-like, the vCT-like layers that form in organoids are often single layers (but not always). Nonetheless, the inside out nature of the organoid system, however is a major limitation of the organoid for studying ST development. However, a functional basal lamina that engages a vCT like progenitor is intact and a continuously renewed vCT is also present. I feel the authors are quick to point out the major limitations of other culture systems, but a balanced acknowledgement of the advantages of specific systems is not implemented.

Reviewer 3 Advance Summary and Potential Significance to Field:
In the manuscript "Placental cytotrophoblast microvillar stabilization is required for cell-cell fusion" Duan et al. employ a human placental explant model to characterize cell shape factors associated with the attainment of cytotrophoblast fusion competence. The authors use first-trimester human placental explants, remove the original syncytiotrophoblast (ST) by trypsinization, and follow the ST regeneration, powered by the spontaneous fusion of the pre-existing vCTB cells. Based on confocal imaging and morphological measurements, they report that the ST-denuded vCTS are the more hypertrophic, elongated cells with more basally located nuclei and increased apical volume, compared to intact, non-ST-denuded vCTs. The authors suggest that formation of extensive microvilli on the apical vCT is a morphological adaptation associated with the fusion competent state. Next, the authors demonstrate that inhibition of ezrin destabilised cytotrophoblast microvilli and affect cytotrophoblast fusion, as shown by reduction of ST markers hCGb and GCM1.
In my opinion, the study is superficial and lacks novelty as the role of ezrin in trophoblast fusion has been established previously (e.g., Pidoux et al., 2014, PMID:25052094). The reported results do not provide any significant advancement in our understanding of trophoblast syncytialization process. As such, I deem this manuscript unsuitable for publication in Development.

Reviewer 3 Comments for the Author:
1) I have some concerns regarding the explant model. How complete and reproducible is the trypsinization-
based ST-denudation process? The protocol does not seem to contain a quality-checkpoint. In vivo, the CT/ST layers remain in a very intimate balance, and the recent findings identified that both CT and ST populations consist of several subtypes. To what extent is this heterogeneity preserved in the explant model?
2) Along the same lines, I'm not convinced how representative and informative the differences in the morphogenic measurements between ST-denuded and control vCTs are due to the nature of this model.
3) The authors did not provide any global gene expression analysis comparing the ST-denuded vCT and their ST-derivatives, to the non-denuded vCT, and the CT and ST placental cells.
4) While the authors confirmed previous reports that ezrin is required for syncytialization, the analysis is very superficial, based on expression of 4 markers.
5) The authors do not corroborate their observations independently, using models such as 2D hTSC or 3D trophoblast organoids.

© 2025. Published by The Company of Biologists under the terms of the Creative Commons Attribution License (https://creativecommons.org/licenses/by/4.0/).

6) The authors suggested that formation of extensive microvilli on the apical vCT is morphological adaptation associated with the fusion competent state. Could the use of life-imaging provide additional insights into a better understanding of this process? An orthogonal stem cell model could be used to observe and corroborate this process. Could microvilli be induced (fluid shear, Miura et al., 2015) to demonstrate that this is required for fusion and not just associated?

Reviewer 4 Advance Summary and Potential Significance to Field:
The study by Duan et al. addresses the mechanisms responsible for fusion of villous cytotrophoblast into the overlying syncytiotrophoblast layer in the human placenta. This is a topic of fundamental translational and clinical importance, for maintaining the integrity of the syncytiotrophoblast layer is critical for successful pregnancy, as evidenced by alterations of this layer in several adverse pregnancy outcomes. Selection of an appropriate experimental model to study this process is challenging because the currently available models all have clear limitations. The authors chose to utilize first trimester explants, the strength of which lie in the fact that they constitute intact placental villi and thus contain all of the villous cells within the accompanying natural architecture. However, as they state in lines 308-311, this model may more accurately represent syncytiotrophoblast regeneration rather than fusion. Overall, it appears that the experiments were carefully performed with necessary controls. In addition, the immunofluorescence images are beautiful. The primary advances that this study provides to the field lies in the association of cytotrophoblast cell shape factors in fusion competence, and elucidation of the roles of ezrin in cytotrophoblast fusion. Overall, the study provides a useful contribution to our understanding of trophoblast biology.

Reviewer 4 Comments for the Author:
Prior to acceptance for publication, however, the authors should address the following points.

Can the authors quantify the results of Figures 2, 3B and 3C? As it currently stands, the observations are strictly qualitative in nature. For example, they state that apical microvilli are present in a “subset” of vCT in first trimester placenta.

The authors state in the methods that they generated z-stacks of the explants in their whole mount studies. It would add to the study if they could include supplemental figures of “z-stack movies” showing the progression of moving through the trophoblast layers, particularly from the 60x imaging.

It would be helpful for readers if the authors would include arrows to indicate the presence of the hair-like F actin projections in Figure 4A.

The statement in lines 241-243 i.e., that the changes in ERVFRD-1 and ERVW-1 expression indicate that blocking ezrin activation stalls vCT differentiation is seemingly a little strong and should be qualified.

As the authors acknowledge, it is currently unclear whether the specific mechanisms/steps underlying fusion of vCTs is the same or different across gestation (although it is reasonable to anticipate that it is highly similar). While their rationale for choosing first trimester placentas makes sense, there are significant differences in the placental microenvironment during the course of gestation. For example, a low oxygen/hypoxic environment prevails in the first trimester, while oxygen levels are higher throughout the remainder of gestation. Additionally, production of interferons increases midway through the second trimester, which corresponds with upregulated expression of antiviral genes, some of which can block vCT usion when expressed at higher levels. Since it appears that the authors cultured the explants at atmospheric oxygen levels, they should at least discuss the potential effects of this parameter, and the overall effects of the placental microenvironment, on their results.

First revision

© 2025. Published by The Company of Biologists under the terms of the Creative Commons Attribution License (https://creativecommons.org/licenses/by/4.0/).

Author response to reviewers' comments

Duan *et al*. (MS ID# DEVELOP/2024/202826)

We would like to extend our thanks to all the reviewers who have given us their feedback and fair assessments of our previous manuscript. Their enthusiasm for the work and input was sincerely appreciated. We have included our point-by-point responses to each comment below and believe that with these revisions that our manuscript is much improved.

In this revised manuscript, some of the most important additions includes: A) the characterization and use of a 3D organoid model of syncytiotrophoblast development to complement our work done in placental explants; B) bulk RNA sequencing data to bolster our transcriptional analyses in our ezrin inhibition experiments and to better characterize our explant and organoid models; C) analyses of polarized endosome trafficking and functional work to alter the localization of the pro-fusogenic glycoprotein CD98 away from the microvilli to identify molecular processes and factors that may be enriched in microvillar structures to support fusion and differentiation. We believe that these and other additions to this manuscript have improved the novelty and depth of knowledge greatly.

Sequencing data is available through the National Center for Biotechnology Information (NCBI) Gene Expression Omnibus (GEO), accession number GSE279087 or through the following reviewer link: https://www.ncbi.nlm.nih.gov/geo/query/acc.cgi?acc=GSE279087. To gain access to the sequencing data enter the following token: czwtesywblijdap.

Reviewer 1:

Reviewer 1 Advance Summary and Potential Significance to Field:
Using a human placental explant model, the authors characterized the morphology of fusion- competent cytotrophoblasts were changed, that form microvilli at the apical membrane. The actin-membrane cross linker protein ezrin within cytotrophoblast microvilli was important to cytotrophoblast fusion. Inhibition of ezrin activation destabilized cytotrophoblast microvilli and prevented cytotrophoblast fusion.

We thank this reviewer for their comments and have included our responses below.

Reviewer 1 Comments for the Author:
The following comments may help to better illustrate the project.
***1)** Fig1A. The picture is not clear, please improve the quality of the image.*
We apologize that these images were not of adequate quality in the original submission. Higher magnification insets have been added in Figure 1A, 1B to allow for better visualization of syncytiotrophoblast (ST) regenerated areas.

***2)** Fig1A. The staining of E-cad can only see monolayers of cells, which does not indicate cell identity, and it is also recommended that the multinucleated areas on the graph be zoomed.*
As indicated above we have included higher magnification inset images in the revised Figure 1A and 1B as recommended to make it easier for readers to visualize the regenerated syncytium. E-cadherin is a well validated villous cytotrophoblast (vCT) marker (PMIDS: 2774055, 1821848) and the signal observed in our experiments is consistent with the observation of others in our pre-trypsinized placental tissue staining and the staining of others (PMIDS: 2774055, 11006324, 12496700) where the underlying mononucleate vCT have an intense junctional signal. We also confirmed the presence of vCT with integrin-α-6 (ITGA6), a second validated vCT marker (PMIDS: 7685095, 1370295). The sustained expression of E-cadherin in the regenerated multinucleate structures is variable even throughout individual explants and appears to be a characteristic of the type of ST formed in the model. This reviewer is correct that there are regions where there is a monolayer of E-cadherin positive multinucleate cells with no underlying mononucleate E-cadherin cells observed after regeneration. This arrangement of multinucleate cells without underlying mononucleate vCT is observed with both dual staining for anti-E-cadherin and phalloidin and double staining with anti-ITGA6 and anti-β human chorionic gonadotropin (β-hCG) [Figure 1A and B]. Regions where compact flattened vCT persist below the regenerated ST or rounded mononucleate

© 2025. Published by The Company of Biologists under the terms of the Creative Commons Attribution License (https://creativecommons.org/licenses/by/4.0/).

cells protrude into the regenerated ST were also observed with both anti-E-cadherin and anti-ITGA6 staining.

3) *Fig2B. The content of the picture lacks specific instructions, please add labels to the picture*
To address this comment, we have updated the pseudo-coloured scanning electron microscopy images to include text labelling of the individual cells (Figure 3B).

4) *Whether ezrin changes in the placental villi at different periods of time*
It is unclear to us exactly what set of experiments this comment refers to; therefore, we thought that the reviewer was most likely to be referring to the localization of ezrin in explant cultured tissue over time. To address this, we have included a representative image of anti- Ezrin staining in explant cultured tissue at 72hrs (Figure S2) showing that anti-Ezrin signal is seen at the apical surface of the multinucleate regenerated ST and in mononucleate E- cadherin positive vCT. This new staining complements the data showing variable strong apically accumulated anti-Ezrin signal in mononucleate E-cadherin positive vCT after 24hrs of culture in Figure 3D.

5) *Fig3B. Please indicate the period of placental villi on the image*
To address this comment, we updated all figure legends to include the gestational age of the tissue displayed in the representative images and have updated Figure 3B with the same information on the upper panel of images. We appreciate that it was not as clear in the initial submission that we only used tissue between the gestational age of 9-13 weeks. We hope that by addressing this comment and including Table 1 and Table S1 which contains more details about the tissue samples used in this study this will be very clear to the readers. Thank you for letting us know this important feature of our data was not clearly communicated.

6) *Page 9, line 191: Please explain why extensive microvilli were not clearly visible in the majority of vCT.*
We thought that this reviewer was most likely asking for an explanation as to why extensively microvilliated vCT were not as abundant in ST-intact placental tissue compared to ST-denuded explants. To address this, we have included summary data of the proportion of microvilliated vCTs in non-trypsinized placental tissue and at 24hrs post-trypsinization (Figure 2G), based on high magnification (63x) confocal images. We have found that microvilliated vCTs are about four times more frequent proportionally at 24hrs post-trypsinization compared to non-trypsinized tissue. This analysis has been included in Lines 155-157. Since the sum of our data in the manuscript suggest microvilli are supportive of vCT fusion, it seems likely that there are negative feedback mechanisms remaining to be discovered within the ST to restrict microvilli formation in the vCT to regulate the position and timing of vCT differentiation to align with ST requirements.

7) *Page 11, line 241, The data results of a significant increase in relative ERVFRD-1 expression and a non-significant increase in ERVW-1 do not directly lead to the conclusions of blocking ezrin activation stalled vCT differentiation into ST. Please adjust the language logic.*
This is an important point to support our conclusions. Therefore, we performed bulk RNA sequencing on trypsin-stripped explant cultured tissue treated +/- 50μM NSC668394 or vehicle control for 48hrs. These new data are presented in Figure 6.

Our bulk RNA-seq analyses (Figure 6E-G) supported our RT-PCR analyses (Figure S6) showing decreased ST marker gene expression (*GCM1, CGB*) in ezrin inhibitor treated explants compared to controls (Figure 6F). It also recapitulated our RT-PCR analysis showing increased expression of fusogens *ERVW-1* And *ERVFRD-1*, markers that have been used by others (PMIDS: 35020896, https://www.biorxiv.org/content/10.1101/2024.07.01.601571v1.full) to identify fusion-competent vCT. Our new data revealed a variable increase in expression of *CGA* and *GREM2*, which have been identified as markers enriched in pre-fusion vCT [*CGA* (PMID: 35020896), *GREM2* (https://www.biorxiv.org/content/10.1101/2024.07.01.601571v1.full)]. Therefore, these new data support that we can reproducibly observe a decrease in ST marker

© 2025. Published by The Company of Biologists under the terms of the Creative Commons Attribution License (https://creativecommons.org/licenses/by/4.0/).

expression, and an increase in fusion-competent marker expression. Together these data support the conclusion that ezrin inhibitor treatment of denuded explants enriches a fusion-competent vCT subpopulation but blocks the development of ST.

8) *The use of NSC668394 to inhibit ezrin and study its effect on cytotrophoblast fusion is innovative. However, a rescue experiment using an ezrin variant that is resistant to NSC668394 inhibition could provide compelling evidence for the specificity of the observed effects.*
Though we agree that this is an excellent control, we feel the literature compellingly supports the specificity of this inhibitor. It has been confirmed that NSC668394 leads to the specific dephosphorylation of ERM proteins with the strongest inhibitory effect on ezrin (PMIDS:21706056, 25801911). The suggested experiment is very technically challenging, and we have thus far been unable to identify the conditions to allow for knockdown and subsequent exogenous protein expression in our explant model due to the speed with which the model regenerates syncytium. Therefore, we did not perform the suggested experiment.

<u>Reviewer 2:</u>
******** Reviewer 2 Advance Summary and Potential Significance to Field:***
The manuscript by Duan et al uses a first trimester placental villi floating system to study actin stabilization, localization, and the importance of the actin-scaffold protein, ezrin, in villous cytotrophoblast (vCT) cell-cell fusion. The explant system is an excellent 3D approach that retains ex vivo morphology and cell-cell communication, aspects that are critical for understanding syncytiotophoblast (ST) development and maintenance.

Using the explant system, the authors show that following ST removal using a trypsin digest, vCT morphology is altered, and filamentous actin protrusions are formed pre ST. The actin scaffold protein ezrin was shown to localize to the apical surface of vCT and further localized to actin extensions. Inhibition of ezrin's ability to bind to actin polymers essentially blocked ST formation, showing that ezrin is essential in ST development. Parts of these findings are novel, while other findings (i.e., inhibition of ezrin on ST formation) are not entirely novel.

The work presented in this manuscript is overall high quality. The images are excellent, and the fundamental experimental design is appropriate. Additionally, the manuscript is well written and easy to follow.

However, the design does have inherent limitations (in detail below), and the conceptual advancement of this work (over previous work showing the requirement of ezrin in trophoblast cell-cell fusion) should be addressed more clearly and carefully. In the manuscript's current state, I feel the findings are overly descriptive, and while the findings do shed some novel insight into ezrin stabilization of actin protrusions important in ST formation, the conceptual and mechanistic advancements are currently limited.

We would like to thank this reviewer for seeing the strengths in our initial submission and their fair and thoughtful assessment and suggestions to improve the manuscript.

Reviewer 2 Comments for the Author:
Specific comments:

1) *More clarity on the clinical samples obtained would strengthen the manuscript. How many placentas were collected? Was biological sex considered? What was the breakdown of gestational age in the samples collected? This data could be tabulated, and individual samples could also be indicated to specific experiments performed.*

This is a very reasonable request, and we apologize for not being more transparent about our samples in the initial submission. We have now included all the requested data that we can obtain in Table 1 and Table S1. This includes the clinical data that we can obtain (gestational age, maternal age) and the biological sex where it was available for the samples which was determined by gel electrophoresis for the *SRY* locus (please see line 695-701) in the material

© 2025. Published by The Company of Biologists under the terms of the Creative Commons Attribution License (https://creativecommons.org/licenses/by/4.0/).

and methods section for details). In total 49 individual placentas were used in our study. Table S1 has also been added to link individual samples to specific experiments. Unfortunately, we do not know the biological sex of the tissue for samples that were collected for the initial submission. Therefore, we have performed analyses to identify whether sex specific difference was observed in our experiments where this information was available, and no sex- specific effects were observed. A statement about this has been included in the discussion (Lines 398-401)

2) *A central concern this reviewer has relates to the possible unintended effect that trypsin has on cell morphology and actin dynamics that is independent of pre ST/fusion vCT biology. This concern could underlie a lot of the study's findings. Trypsin may also lead to stress- induced protein-complex distributions that are typically not occurring in non-trypsin stressed cells. Would subjecting TSCs to a fusion assay as an alternative way to measure cell-cell fusion further support and strengthen the study's findings?*
https://doi.org/10.1016/0014-4827(78)90029-0
https://doi.org/10.1016/j.ymeth.2013.02.014

The reviewer brings up very valid points. To address this, we have repeated key experiments using a trophoblast stem cell (TSC) organoid model developed in our lab. Since this is a new model, we included the characterization of it in Figure 4. In summary, we used the CT29 line derived by Okae *et al*. that were maintained in 2D before passaging and moving the cells into the trophoblast organoid medium first published by Turco *et al*. in bioreactor culture. The cells aggregate and display marker gene expression profiles and protein expression by 18hrs in bioreactor culture suggesting the cells are primarily vCT populations. By 48hrs in bioreactor culture there is the extensive formation of multinucleate cells that have protein expression of key ST markers (ß-hCG, syndecan-1) and bulk RNA-seq analyses show the up-regulation of ST marker genes. ß-hCG can also be detected in the culture medium of the organoids after 48hrs of culture.

Critically, after 18hrs in bioreactor culture, microvilli could be observed on the surface of many cells and between mono-nucleate vCT (Figure 4F), supporting our observations in explants and in intact tissue that microvilli accumulate in select vCT populations. Experiments were also performed with Ezrin inhibitor in the organoid model, and fusion was strongly inhibited in this model, aligning with our data in explants (Figure 7). Importantly, there were some key differences between the organoid model and the explants regarding the outcome of ezrin inhibitor treatment. This is further elaborated below in our response to point 3 from this reviewer. Overall, we believe the inclusion of this second model greatly strengthens our conclusions and we thank the reviewer for their constructive and, though challenging, fair comments to improve our manuscript in this aspect.

3)*There is also a concern about the effect of higher concentrations of NSC668394 impacting cell viability and proliferation (independent of the effects of this inhibitor on ezrin threonine phosphorylation). Can the authors show that the increasing doses of NSC668394 in SCT denuded explants is not inhibiting SCT formation in part due to impaired vCT viability?*

This is, again, a very fair concern. To address this explant cultured tissue was assessed for proliferation (EdU incorporation in E-cadherin positive cytotrophoblasts) in data that is now included in Figure S3. No difference in vCT proliferation was observed with NSC668394 treatment. To assess for viability, we stained with an anti-cleaved-caspase-3 antibody, which detects active caspase-3, a key mediator of vCT apoptosis (PMID: 23102999), and we quantified cleaved-caspase-3 positive E-cadherin positive cytotrophoblasts. This data is now included in Figure S4. Again, no change was observed between control vs. NSC668394 cl-caspase-3 positive vCT numbers. Therefore, in the explant model the inhibition of cell fusion observed with inhibitor treatment is not dependent on changes in vCT proliferation and viability.

However, 25µM NSC668394 treatment did affect proliferation and cell viability in our organoid model (Figure 7). Organoids treated with ezrin inhibitor had visibly reduced size

© 2025. Published by The Company of Biologists under the terms of the Creative Commons Attribution License (https://creativecommons.org/licenses/by/4.0/).

(Figure 7A). The same result was observed in pilot experiments with 50µM NSC668394. Due to the configuration of our bioreactor apparatus, we were only able to carry out control vs. a single dose of inhibitor at a time, therefore we selected to continue with 25µM NSC668394. Alongside the reduction in size, there was a decrease in proliferation (Figure 7B) and cell viability (Figure 7C) in cells within ezrin inhibitor treated organoids. We have also stained for E-cadherin and phalloidin to assess fusion (Figure 7D) and for ST markers like ß-hCG (Figure 7E) and syndecan-1 (Figure 7F). Critically, there is little to no signal only in ezrin inhibitor treated organoids stained with anti-ß-hCG and anti-SDC-1. Therefore, there are important differences in the outcomes of ezrin inhibition on trophoblastic cell populations between our explant model and the organoids, but the conserved feature being a block in ST formation. RNA-seq analyses also revealed multiple conserved GO terms between treated explants and organoids, showing that many conserved effects were observed between the models despite the additional effects on proliferation and death seen with only one model.

4) *Related to the above comment, while the authors show convincing evidence using the ezrin inhibitor that ezrin-mediated actin interactions and apical polarity (and perhaps ezrin's other function as an AKAP that influences PKA localization) are important for ST development, the concern that the inhibitor may be contributing to suboptimal cell viability is a limitation of this approach. Are there other complementary ways to inhibit ezrin that would ultimately support the study's findings? Is knock-down feasible in explants? If not, would this not justify the use of a non-explant model that is amenable to ezrin knock-down to verify these findings?*

This is an excellent point that we will be certain to incorporate into our studies in the future. Unfortunately, the explant model is not amenable to ezrin knock down studies due to the length of time required to achieve a significant decrease in ezrin in trophoblasts (PMID: 25052094). Pidoux et al. showed that 48hrs treatment time is necessary to achieve significant knockdown, meaning siRNA could be added 24h after explant trypsinization, but then KD wouldn't be achieved until 72hrs in culture when most of the ST has already regenerated. Another option would be to first knockdown ezrin via transfection of the CT29 TSC before moving them into organoid medium and bioreactor culture, but this would take more time to develop and optimize than we had in this revision period, since we had to validate the organoid model. Our ezrin inhibitor data in the TSC organoid model also suggests that ezrin knockdown may significantly affect proliferation and viability in this model, further complicating the optimization timeline. Therefore, we tried to address this point by treating our explants with SB633825, a LOK1/Tie2 inhibitor (PMID:26501955). LOK1 phosphorylates ezrin at the Thr567 residue, leading to ezrin activation and linkage of the actin cytoskeleton to the plasma membrane in many cell types (PMIDS: 19255442, 28430576). We hypothesized that by inhibiting this upstream kinase that we would observe the same findings as our ezrin inhibitor treatments (i.e. decrease in fusion). Unfortunately, at the doses reported to inhibit LOK1, we were unable to observe a substantial and reproducible decrease in signal intensity of anti- phospho-Thr 567 Ezrin signal (Figure 1 below). This suggests that LOK1 is not the primary kinase responsible for Ezrin phosphorylation and activation in vCT, aligning with data from Miura et al, who showed that AKT is the primary kinase that regulates Ezrin Thr-567 phosphorylation and therefore activation in Bewo (PMID: 26563429). AKT is a central kinase in cellular homeostasis, therefore using AKT inhibitors would not be specific enough to support our ezrin inhibitor data due to a lack of specificity. Therefore, we have been unable to address this point in the revised manuscript. In our future work we will incorporate ezrin knockdown experiments as our work with the inhibitor has identified many potential additional roles that this protein plays in vCT that will be followed up on.

NOTE: We have removed unpublished data that had been provided for the referees in confidence.

Figure 1: SB633825 (LOK1 inhibitor) does not significantly affect activated p-Ezrin levels in explants. Representative XY plane confocal microscopy images of explants treated ± 20µM SB633825 for 6hrs after ST denudation; i = merged anti-p-Ezrin (magenta), anti-E- cadherin (green), Hoechst 33342 (nuclei; blue) signals; ii = isolated anti-p-Ezrin (greyscale) signals.

© 2025. Published by The Company of Biologists under the terms of the Creative Commons Attribution License (https://creativecommons.org/licenses/by/4.0/).

***5)** SCT coverage: In the methods line 420, the authors indicate that Ecad, phalloidin, and Hoechst stains were used to facilitate the quantification of SCT coverage. Indeed, the equation for this was shown. However, what defining parts of this stain helped to identify the ST compared to the vCT? Also, in areas where vCT are predicated to have been exhausted (i.e., not present), how does this affect the ST coverage calculation?*

We appreciate that this was not explained clearly enough in the original submission. We have expanded our explanation of this analysis in the methods section under "Confocal microscopy image capture and analyses" (Lines 611-612). VCTs were defined as mononucleated cells with E-cadherin signal at the periphery. ST was defined as multinucleated cells, determined through the visualization of multiple nuclei within a phalloidin (F-actin) encircled apical structure.

We have not observed an any areas where vCTs are completely absent within a 20x confocal microscopy frame. Areas are frequently observed where there are discontinuous E-cadherin-positive mononucleate cells underlying multinucleate structures, and E-cadherin positive mononucleate cells are also observed within a multinucleated structure or between two multinucleated structures. We recognize now how our language describing these areas of ST regeneration was confusing. Therefore, we have also updated the text in the results section to be clearer.

***6)** The authors, through the execution of excellent imaging analyses, describe the morphology of vCT that are stated as pre-fusing. How do the authors know these are pre-fusing vCT? If this can't be done, perhaps a tempering of the language that describes these morphological changes may be needed.*
We appreciate that we cannot unequivocally prove that the cells are pre-fusing vCT in our morphological analysis and a similar comment was raised by Reviewer 1. To further support the hypothesis that ST denuded explants are enriched in pre-fusing vCT, we intend to perform deconvolution analyses of our bulk RNA-seq data to show changes in vCT populations in a separate methodological publication we are preparing on our explant model. For now, we have tempered our language to better convey that to unequivocally prove this is not possible with current knowledge and methodology.

***7)** Extensive microvilli versus podosomes; I am not sure that the apical structure of ezrin positive microvilli is sufficient to distinguish a microvilli from a podosome. Have the authors co-stained with podosome markers to confirm this?*
This is an interesting point. Podosome are defined as broad actin-based membrane projections (0.5-2µm in diameter) (PMID:21697900), versus a microvillus being defined as a fine actin-based membrane projection (50-350nm in diameter) (PMID: 33013920). In our SEM images the membrane protrusions that are observed on the vCT surface are <0.5µm in width and similarly the projections we observed by TEM in intact tissue are also <0.5µm in width.
We have updated the text to include more reasoning for our definition of microvilli in the manuscript.

***8)** What is the purpose or role of stabilized microvilli protrusions in ST formation? They seem to be required for cell fusion, but this study does not really provide insight into how these structures are contributing to cell fusion. Are these features providing a route for specific fusogenic proteins or other cargo like vacuoles? The relationship between ezrin and PKA is perhaps something worth examining as well as it is unclear to this reviewer what ezrin is doing: 1) controlling actin dynamics/stabilization; 2) functioning as an AKAP and modulating PKA localization and function. This comment relates to the overall impact of the paper and the conceptual advance the paper may/may not have.*
This comment is one of the most difficult to address, though we understand and agree with the reviewer's comments about the conceptual advancement that additional work would bring. Therefore, we performed two separate sets of experiments to query how microvilli may support vCT fusion.

Firstly, as suggested by the reviewer, we have stained for markers of the endo-lysosomal system to address whether vCT microvilli function as signaling hubs like T-cell microvilli and intestinal microvilli. We see abundant localization of early endosomal antigen-1 (EEA1) positive vesicles within the microvilli and accumulated at the apical region of vCT in our explant regeneration

© 2025. Published by The Company of Biologists under the terms of the Creative Commons Attribution License (https://creativecommons.org/licenses/by/4.0/).

model (Figure 8). Short term NSC668394 treatment (2hrs) leads to a re- localization of anti-EEA-1 signal within the cells so that they are significantly less polarized and changes the size distribution of these EEA-1-positive bodies were also observed (Figure 8A, 8B). Therefore, with the inclusion of this new data we propose that microvilli may function as signaling hubs to relay polarized messages within the cells. With the disruption of microvillar structure via ezrin inhibitor treatment, early endosomal distribution is less polarized, and this would result in spatially altered activation of signaling cascades since endosomes mediate and regulate the activity of key cell signalling mediators, including but not restricted to receptor tyrosine kinases, G-protein coupled receptors, and mediators of the MAPK pathway (PMID: 22108513).

Secondly, we performed experiments that support the idea that vCT microvilli allow for the enriched localization of fusogenic proteins. Unfortunately, we have been unable to identify antibodies that work for syncytin family proteins. So instead, we carried out experiments focused on CD98, a protein that supports Bewo trophoblastic cell line fusion (PMIDs:12740424, 17258169) and plays an important role in multiple other membrane fusion events in other cell types (PMIDs: 22499670, 8922468, 9138296, 12925876, 11243701). This work is presented in Figure 9. We found that CD98 is enriched at vCT microvilli in intact tissue and cultured explants and at the apical surface (Figure 9A,B). We then took advantage of the fact that CD98 signaling can be manipulated by cross-linking monoclonal antibody treatment (PMIDs:12181350,12716892,17258169). Antibody cross-linking experiments in explants selectively targeted apically localized vCT CD98 and led to decreased apical and microvillar CD98 expression (Figure 9D, 9E) and reduced vCT fusion (Figure 9F, 9G). Therefore, the sum of these data suggests that the apical localization of CD98 in these microvillar structures is necessary for fusion and support the idea that microvillar structures may serve to concentrate pro-fusogenic machinery.

9) *Line 120: The authors provide a rationale for solely using the floating villous explant system to study cell fusion. The authors indicate that the explant system provides the advantage of a 3D system where progenitor vCT reside on top of a basement membrane, and where cell-cell fusion into SCT occurs spontaneously. These features are stated to not occur in 2D TSC cultures, current organoid systems, and 2D primary CTB cultures. I would argue that this interpretation is not 100% accurate. Many cells lay down matrix, including basement membrane-associated proteins like laminin in 2D culture; trophoblast organoids, while differentiating into SCT in an inverse orientation, nonetheless have a laminin-rich basement membrane for vCT like cells to situate on, and similar to primary vCT and floating explants models, cell fusion is also spontaneous. These nuances are important as there are also limitations to the explant system (exposure to trypsin, villous viability, lack of progenitor renewal, difficulties in mechanistic interrogation) that should be also considered and limit theoverall scope of the study and the interpretation of the study's findings. I would argue that applying the explant system in conjunction with another supporting cell-based system would provide important redundancy and improve overall reliability/robustness of the data.*
This is a very fair point, and we were too strong in our previous comments. We agree that the addition of organoid data has strengthened our manuscript, and that using multiple models to support or refute observations is very important. We have tempered our language and have included in the discussion a section about the aspects of our analyses that were conserved between the explant and organoid model, and those that were not conserved. We were not able to perform all our work characterizing what microvilli may be doing (discussed in point 8 to this reviewer) in both models. This will be carried out in the future to further investigate how polarized endosomal trafficking and accumulation of CD98 influence fusion.

10) *Data shown in Figure 1A. Trypsin-mediated removal of the outer syncytial layer shows that it was effective. However, following 72 hrs, it is not entirely clear to this reviewer that a SCT layer spontaneously reformed. The image is not magnified enough to appreciate distinctions between vCT and newly-formed syncytiotrophoblast. Further, it appears that the arrows are pointing to Ecad positive cells; are not multinucleated SCT Ecad low or null relative to vCT? The Cd49f signal is likewise hard to discern between vCT and new SCT. It is clear that hCG is robustly present by 72 hrs, but the magnification/clarity of the image make it hard to visualize a distinct SCT layer.*
As for reviewer 1- this has been addressed with improved images and added insets. We hope that the new image panels make it easier for readers to visualize the ST layer.

© 2025. Published by The Company of Biologists under the terms of the Creative Commons Attribution License (https://creativecommons.org/licenses/by/4.0/).

Minor:

Line 125: Sentence suggests that syncytial removal required 24 hrs of trypsin exposure. I don't think this is the case.

This reviewer is correct: we meant to say that the pre-existing ST is removed by 24hrs of explant culture, after a 7min trypsin exposure. This sentence has been changed in Lines 103-104.

Line 329: The authors statement that in trophoblast organoids the matrix is surrounding a "ball" of progenitor trophoblasts is somewhat inaccurate. While the overall shape is ball-like, the vCT-like layers that form in organoids are often single layers (but not always). Nonetheless, the inside out nature of the organoid system, however is a major limitation of the organoid for studying ST development. However, a functional basal lamina that engages a vCT like progenitor is intact and a continuously renewed vCT is also present. I feel the authors are quick to point out the major limitations of other culture systems, but a balanced acknowledgement of the advantages of specific systems is not implemented.

This is an excellent point. In hindsight we were too eager to point out limitations in other models. With the addition of the TSC organoid model, we have added a section in the discussion to compare our explants with the organoids (Lines 381-398) and have removed the previous discussion.

Reviewer 3:

******** Reviewer 3 Advance Summary and Potential Significance to Field:***

In the manuscript "Placental cytotrophoblast microvillar stabilization is required for cell-cell fusion" Duan et al. employ a human placental explant model to characterize cell shape factors associated with the attainment of cytotrophoblast fusion competence. The authors use first-trimester human placental explants, remove the original syncytiotrophoblast (ST) by trypsinization, and follow the ST regeneration, powered by the spontaneous fusion of the pre-existing vCTB cells. Based on confocal imaging and morphological measurements, they report that the ST-denuded vCTS are the more hypertrophic, elongated cells with more basally located nuclei and increased apical volume, compared to intact, non-ST-denuded vCTs. The authors suggest that formation of extensive microvilli on the apical vCT is a morphological adaptation associated with the fusion competent state. Next, the authors demonstrate that inhibition of ezrin destabilised cytotrophoblast microvilli and affect cytotrophoblast fusion, as shown by reduction of ST markers hCGb and GCM1.

In my opinion, the study is superficial and lacks novelty as the role of ezrin in trophoblast fusion has been established previously (e.g., Pidoux et al., 2014, PMID:25052094). The reported results do not provide any significant advancement in our understanding of trophoblast syncytialization process. As such, I deem this manuscript unsuitable for publication in Development.

We appreciate this reviewer's viewpoint and believe that the extensively revised manuscript, including new data about other potential functions of microvilli in vCT fusion, has a much-improved novelty and reveals considerable new knowledge beyond the observations of Pidoux *et al*.

Reviewer 3 Comments for the Author:

1) *I have some concerns regarding the explant model. How complete and reproducible is the trypsinization-based ST-denudation process? The protocol does not seem to contain a quality-checkpoint. In vivo, the CT/ST layers remain in a very intimate balance, and the recent findings identified that both CT and ST populations consist of several subtypes. To what extent is this heterogeneity preserved in the explant model?*

This is an important concern, and we have included multiple new pieces of data to further characterize our explant model and address these concerns (please see Figure 1 and Figure S1). We have now included data showing ST coverage at 72hrs and 24hrs (Figure S1A),

© 2025. Published by The Company of Biologists under the terms of the Creative Commons Attribution License (https://creativecommons.org/licenses/by/4.0/).

characterizing vCT proliferation (Figure S1C, D), and performed bulk RNA-seq analysis on our explant model to expand our analysis of marker gene expression for the multiple trophoblast sub-types that exist in explant cultured tissue (Figure 1D, Figure S1B). This new data shows that our method for ST denudation using trypsin is very complete and reproducible (between 90-98% of the ST is removed) but the degree of ST regeneration post-denudation varies from donor to donor. As seen in Figure S1C, ST coverage (cross-sectional area of the ST divided by cross-sectional area of the vCT) at 24hrs is consistent at 0.02-0.10 but at 72hrs varies from 0.50 to 1.50. This is why fusion and RT-PCR data with explant tissue is displayed as normalized data to patient-matched controls.

2) *Along the same lines, I'm not convinced how representative and informative the differences in the morphogenic measurements between ST-denuded and control vCTs are due to the nature of this model.*

We have incorporated a response to this criticism into our discussion (Lines 433-435). Observations in our explant and organoid models support the conserved formation of F-actin- and ezrin-positive microvilli in vCT prior to fusion and the critical role of ezrin-mediated processes for fusion. We chose to focus exclusively on microvilli in this manuscript, and our experiments were not designed to directly test whether the other features identified are obligate for differentiation. But the importance of the other vCT morphological characteristic reported here can be addressed in the future with the development of live-cell imaging methodologies with organoids and targeting of, for example the microtubular network, which is important for nuclear positioning in other cell type.

3) *The authors did not provide any global gene expression analysis comparing the ST-denuded vCT and their ST-derivatives, to the non-denuded vCT, and the CT and ST placental cells.*

To better characterize our model, we have collected bulk RNA sequencing data on the explant ST regeneration model as seen in Figure 1D, 1E and referred to above (Point 1). We have collected RNA from donor-matched placental explants at 24hrs post-trypsinization when the ST is maximally removed and at 72hrs post-trypsinization where we see ST regeneration. In our differentially expressed gene (DEG) analyses, we probed for gene transcripts that are commonly used in single cell RNA sequencing studies to mark key trophoblastic subpopulations in the human placenta. We have included multiple genes that are enriched in bipotential TSCs (*ITGA2, CDX2, BCAM1*) (PMIDS: 38359834, 35020896), cytotrophoblasts (*ITGA6, TEAD4, GATA3, TFAP2X, TP63*) (PMIDS: 38359834, 35020896), fusion-competent vCTs (*CGA, GREM2, ERVFRD-1*) (PMIDS: 35020896; https://www.biorxiv.org/content/10.1101/2024.07.01.601571v1.full), EVT (*HLA-G, NOTCH2, ITGA5, ITGA1, CD9*) (PMIDS: 38359834, 35020896), as well as additional ST marker genes (*CSH, CGB, PAPPA, PSG, GCM1, SDC1*) to better characterize our explant and organoid models.

4) *While the authors confirmed previous reports that ezrin is required for syncytialization, the analysis is very superficial, based on expression of 4 markers.*

To address this point, we have performed more in-depth analysis of our ezrin inhibitor treated explants also using bulk RNA-seq (Figure 6E, 6F). As described in our response to Reviewer 3, part 3, we have included multiple genes that are enriched in bipotential TSCs, vCT, fusion- competent vCTs, EVT, as well as additional ST marker genes to strengthen our conclusions. Consistent with our protein expression (staining) data and RT-PCR analyses, these new data show that compared to controls, ezrin inhibitor treated explants express relatively lower levels of ST marker genes and have increased expression of marker genes associated with fusion- competent vCTs (Figure 6F). We feel this conclusion is now very well supported.

5) *The authors do not corroborate their observations independently, using models such as 2D hTSC or 3D trophoblast organoids.*
Please see our response to Reviewer 2, part 2A above. Analyses with an organoid model has now been included in the manuscript.

6) *The authors suggested that formation of extensive microvilli on the apical vCT is*

© 2025. Published by The Company of Biologists under the terms of the Creative Commons Attribution License (https://creativecommons.org/licenses/by/4.0/).

morphological adaptation associated with the fusion competent state. Could the use of life-imaging provide additional insights into a better understanding of this process? An orthogonal stem cell model could be used to observe and corroborate this process. Could microvilli be induced (fluid shear, Miura et al., 2015) to demonstrate that this is required for fusion and not just associated?
We agree a live cell imaging system would be ideal for showing that microvilli are formed, but this is a very technically difficult experiment due to the need for membrane and actin labelling, the optimization of live cell imaging conditions, and the spontaneous nature of vCT fusion in our organoid model. We plan to develop the conditions necessary to perform this sort of experiment in the future, but this would take considerably longer than this revision period.

The second experiment suggested (inducing microvilli with shear stress) is an interesting idea. Sparse microvilli are also present under static conditions in Bewo choriocarcinoma cells in Miura *et. al's* work, therefore, shear stress would be useful in demonstrating whether increased microvilli would enhance fusion, but not to show that they were obligatory for fusion. In fact, this experiment has recently been published (PMID:37930227). These authors saw an increase in fusion in iPSC-derived cytotrophoblasts exposed to flow and increased microvilli on the cells with flow induction. Therefore, we do not plan to address this experimentally, but this topic and reference is now included in the discussion (Line 438-440), as it supports that microvilli are important for trophoblastic cell fusion.

Reviewer 4:

******* Reviewer 4 Advance Summary and Potential Significance to Field:**
The study by Duan et al. addresses the mechanisms responsible for fusion of villous cytotrophoblast into the overlying syncytiotrophoblast layer in the human placenta. This is a topic of fundamental translational and clinical importance, for maintaining the integrity of the syncytiotrophoblast layer is critical for successful pregnancy, as evidenced by alterations of this layer in several adverse pregnancy outcomes. Selection of an appropriate experimental model to study this process is challenging because the currently available models all have clear limitations. The authors chose to utilize first trimester explants, the strength of which lie in the fact that they constitute intact placental villi and thus contain all of the villous cells within the accompanying natural architecture. However, as they state in lines 308-311, this model may more accurately represent syncytiotrophoblast regeneration rather than fusion. Overall, it appears that the experiments were carefully performed with necessary controls. In addition, the immunofluorescence images are beautiful. The primary advances that this study provides to the field lies in the association of cytotrophoblast cell shape factors in fusion competence, and elucidation of the roles of ezrin in cytotrophoblast fusion. Overall, the study provides a useful contribution to our understanding of trophoblast biology.

We thank this reviewer for this very positive assessment of our work.

Reviewer 4 Comments for the Author:
Prior to acceptance for publication, however, the authors should address the following points.

1) *Can the authors quantify the results of Figures 2, 3B and 3C? As it currently stands, the observations are strictly qualitative in nature. For example, they state that apical microvilli are present in a "subset" of vCT in first trimester placenta.*
This has been carried out and has been included in Figures 2G, 3C, and 4C. Thank you for this suggestion, as it strengthens our conclusions and makes it easier for the reader.

2) *The authors state in the methods that they generated z-stacks of the explants in their whole mount studies. It would add to the study if they could include supplemental figures of "z-stack movies" showing the progression of moving through the trophoblast layers, particularly from the 60x imaging.*
These have been included with the revised manuscript as Movies 1, 5, and 6. We agree that this makes it easier for readers to understand our analyses and have used movies to show characteristics of our organoid model as well in the resubmission (Movies 2-4)

© 2025. Published by The Company of Biologists under the terms of the Creative Commons Attribution License (https://creativecommons.org/licenses/by/4.0/).

***3)** It would be helpful for readers if the authors would include arrows to indicate the presence of the hair-like F-actin projections in Figure 4A.*
Arrows have been added in what was formerly Figure 4A (Figure 6A in the current manuscript).

***4)** The statement in lines 241-243 i.e., that the changes in ERVFRD-1 and ERVW-1 expression indicate that blocking ezrin activation stalls vCT differentiation is seemingly a little strong and should be qualified.*
This point was raised by multiple reviewers and is a very fair considering the data included in the previous version of the manuscript. Please see Reviewer 1 Comment 7 for a full response.

***5)** As the authors acknowledge, it is currently unclear whether the specific mechanisms/steps underlying fusion of vCTs is the same or different across gestation (although it is reasonable to anticipate that it is highly similar). While their rationale for choosing first trimester placentas makes sense, there are significant differences in the placental microenvironment during the course of gestation. For example, a low oxygen/hypoxic environment prevails in the first trimester, while oxygen levels are higher throughout the remainder of gestation. Additionally, production of interferons increases midway through the second trimester, which corresponds with upregulated expression of antiviral genes, some of which can block vCT fusion when expressed at higher levels. Since it appears that the authors cultured the explants at atmospheric oxygen levels, they should at least discuss the potential effects of this parameter, and the overall effects of the placental microenvironment, on their results.*
We agree that oxygen tension is an important factor to consider in all trophoblast differentiation work. We acknowledged that we were unable to emulate this physiologic parameter in Lines 404-407.

Second decision letter

MS ID#: DEVELOP/2024/202826

MS TITLE: Placental cytotrophoblast microvillar stabilization is required for cell-cell fusion

AUTHORS: Wendy K. Duan; Sumaiyah Z. Shaha; Juan F. Garcia Rivas; Bethan L. Wilson; Khushali J. Patel; Ivan K. Domingo; Meghan Riddell

I have now received all the referees reports on the above manuscript, and have reached a decision. The referees' comments are appended below, or you can access them online: please go to BenchPress and click on the 'Manuscripts with Decisions' queue in the Author Area.

As you will see, the overall evaluation is positive and all reviewers are enthusiastic about the study, as are we. We would like to publish a revised manuscript in Development, provided that the referees' remaining comments can be satisfactorily addressed. Please attend to all of the reviewers' comments in your revised manuscript and detail them in your point-by-point response. I believe that the comments can be addressed through changes to the text and minor updates to the figures. If you do not agree with any of their criticisms or suggestions explain clearly why this is so. If it would be helpful, you are welcome to contact us to discuss your revision plans in greater detail.

Reviewer 1
Advance Summary and Potential Significance to Field:
The revised manuscript shows clear improvement over the initial submission. The authors have made a significant effort to address prior concerns, including enhanced figure clarity, additional experiments, and expanded narrative sections.

© 2025. Published by The Company of Biologists under the terms of the Creative Commons Attribution License (https://creativecommons.org/licenses/by/4.0/).

While many previous comments have been adequately addressed, some issues remain unresolved, and new concerns arise from the additional data. Below is a detailed evaluation of the manuscript.

Reviewer 1 Comments for the Author:
Responses to previous comments

Improvement of Figures (1A, 2B, etc.): The authors provided higher magnification insets and annotations for figures, improving their interpretability. This response is satisfactory and resolves the earlier concern.

Contradictory data on ITGA6 and fusogens (Figure 6): While the authors provided a plausible explanation linking transcriptional and post-translational effects, the conflict between ITGA6 downregulation and increased fusogen expression remains unresolved. This concern has only been partially addressed.

Trypsin effects on cytotrophoblasts: The manuscript still lacks a control group without trypsin treatment to evaluate potential artifacts. This critical issue has not been addressed.

Rescue experiment for ezrin specificity: The authors explain technical challenges preventing this experiment but fail to provide alternative data. This limitation has been partially addressed but remains a significant gap.

Statistical and sample size issues: Some analyses (e.g., Figures 4D and 7G) still rely on small sample sizes, weakening their conclusions. Additional biological replicates are necessary. This issue has not been adequately addressed.

Major concerns

Microvillar stabilization mechanisms. The manuscript demonstrates a clear role for microvilli in fusion but lacks mechanistic depth. Exploring signaling pathways or interactions beyond ezrin phosphorylation would add value.

Model limitations. The differences between the explant and organoid models are underexplored. Clarifying their biological relevance and potential clinical implications is necessary.

Contradictory data. The apparent disconnect between increased fusogen expression and impaired fusion (Figures 6 and 7) suggests unexplored regulatory mechanisms. A more in-depth discussion or additional data are needed.

Statistical robustness. Analyses with insufficient sample sizes compromise the reliability of conclusions. Either provide more replicates or justify the sample sizes used.

Minor comments

Explanations for quantitative metrics. Figures 2D and 2F require clear definitions of parameters like shape factor and apical volume.

N-values in figures. Statistical figures should include n-values for transparency and reproducibility.

Figure clarity. Magnified insets for Figures 6D and 6F would help highlight critical observations.

Concentration of ezrin inhibitor. Justify the concentrations of NSC668394 used, supported by experimental data.

Language and grammar. Simplify and refine phrasing throughout the manuscript to improve readability.

Recommendation

The manuscript has made significant progress but still requires further revisions.

© 2025. Published by The Company of Biologists under the terms of the Creative Commons Attribution License (https://creativecommons.org/licenses/by/4.0/).

Addressing the major and minor points outlined above will strengthen the study's rigor and impact. A minor revision is recommended to resolve these issues comprehensively.

Reviewer 2
Advance Summary and Potential Significance to Field:
The authors's revisions now provide a much more developed mechanistic insight into how ezrin-mediated actin protrusions are facilitating trophoblast cell-cell fusion. Using two excellent model systems, the authors show that ezrin is important for potentially creating a cellular structure that accumulates fusion proteins. This work is novel, builds on previous findings of ezrin, and advances the fields knowledge.

Reviewer 2 Comments for the Author:
The authors have made significant efforts to improve their manuscript.

In response to my previous comments, the authors attempted or addressed every single point. New experiments performed improve the overall quality of their work, and importantly, provide mechanistic insight into the role of actin-rich microvilli protrusions in trophoblast cell-cell fusion. In particular, their use of the CT29 TSC line cultured in a bioreactor to induce syncytialization in correct orientation is to be commended. Relatedly, work using bulk RNA-seq to examine gene pathways that are impacted in response to ezrin inhibition in the organoid system also add strength to the revised manuscript. Lastly, new work examining the localization of CD98 within actin-rich structures provides new insight into how actin-rich protrusions are contributing to trophoblast syncytialization, and the role in which ezrin is facilitating this potential trafficking event.

As indicated, this revision is a marked improvement over the initial submission.

My only ask is that the authors perform a more thorough comparison between the explant and organoid bulk RNA-seq analyses. Because the effect of the ezrin inhibitor in the organoids impacted cell viability and proliferation, I feel a more careful examination in warranted. Integrating both RNA-seq objects and examining global differences/similarities could be helpful (PCs, PCC, or cluster analyses). I would expect there to be differences between the two systems, but visualizing these differences with a statistical inference will bolster the data as there seem to be important caveats related to the organoid data.

Reviewer 4
Comments for the Author:
Comments were uploaded and are available as a PDF at https://submit-dev.biologists.org.

Second revision

<u>Author response to reviewers' comments</u>

Duan *et al*. (MS ID# DEVELOP/2024/202826)

We would like to extend our thanks to all the reviewers for their enthusiasm for our revised manuscript. Point-by-point responses have been included below to address reviewer comments.

<u>Reviewer 1:</u>
Advance Summary and Potential Significance to Field:
The revised manuscript shows clear improvement over the initial submission. The authors have made a significant effort to address prior concerns, including enhanced figure clarity, additional experiments, and expanded narrative sections. While many previous comments have been adequately addressed, some issues remain unresolved, and new concerns arise from the additional data. Below is a detailed evaluation of the manuscript.

© 2025. Published by The Company of Biologists under the terms of the Creative Commons Attribution License (https://creativecommons.org/licenses/by/4.0/).

We thank this reviewer for their comments and have included our responses below. We believe with the revisions added that our manuscript is clearer.

Reviewer 1 Comments for the Author:
Responses to previous comments:
1. Improvement of Figures (1A, 2B, etc.): The authors provided higher magnification insets and annotations for figures, improving their interpretability. This response is satisfactory and resolves the earlier concern.

Thank you for your comment concerning our figures. We agree that adding higher magnification insets improved the clarity.

2. Contradictory data on ITGA6 and fusogens (Figure 6): While the authors provided a plausible explanation linking transcriptional and post-translational effects, the conflict between ITGA6 downregulation and increased fusogen expression remains unresolved. This concern has only been partially addressed.

We apologize but we are unsure where the contradiction is with *ITGA6* downregulation and fusogen upregulation. Single cell RNA-sequencing on first trimester placenta conducted by Shannon *et al.* (2022) (PMID: 35020896) shows that the *ERVFRD-1* and *ERVW1* positive fusion-competent vCT subpopulation (what they called ST precursors) have downregulated average *ITGA6* expression compared to other vCT subpopulations. This aligns with our data showing that ezrin inhibition in explants causes upregulation in genes associated with fusion-competent vCTs. Coupled with our data showing that functional fusion is impaired with ezrin inhibition, this suggests that fusogen post-transcriptional expression, localization, or function is impaired. This is further expanded on below in response to Reviewer 1, Major concerns Point 3.

3. Trypsin effects on cytotrophoblasts: The manuscript still lacks a control group without trypsin treatment to evaluate potential artifacts. This critical issue has not been addressed.

We apologize that we did not explicitly address this issue that reviewer 2 had brought up in our previous response to reviewers, but with the addition of the TSC, this issue has been addressed to the best of our abilities. TSC cells are passaged with TrypLE, an enzymatic alternative to trypsin that is gentler than traditional porcine trypsin (Gibco, cat: 12604-013). Therefore, this is not a perfect control since there is still enzymatic cleavage of surface proteins, but vCT in both our explant model and the organoid model become highly decorated in microvilli. We also expanded our analyses examining the degree of microvilliation that was observed in intact placenta in our revised manuscript which were not cultured after collection. These analyses revealed that vCT with varying degrees of apical microvilli are also present without exposure to trypsin. Altogether these results strongly suggest that the accumulation of apical microvilli on vCT is not an artifact of *in vitro* manipulation.

4. Rescue experiment for ezrin specificity: The authors explain technical challenges preventing this experiment but fail to provide alternative data. This limitation has been partially addressed but remains a significant gap.

We agree that performing a rescue experiment of reintroducing a phospho-resistant ezrin would serve as a strong control for our ezrin inhibitor treatment. As stated in the previous round of revisions, we will be developing and optimizing a protocol for knocking down ezrin in primary human trophoblasts or trophoblastic stem cell (TSC) lines in 2D culture, reintroducing phospho-resistant ezrin, and assessing ST formation in 3D organoid culture in the future.

5. Statistical and sample size issues: Some analyses (e.g., Figures 4D and 7G) still rely on small sample sizes, weakening their conclusions. Additional biological replicates are necessary. This issue has not been adequately addressed.

This reviewer raises a valid point of sample size with our organoid model analyses. We agree that the power of these analyses would be stronger with more experimental replicates and with the addition of biological replicates using different TSC lines. Due to the cost associated with RNA-sequencing we sent two replicates of CT29 trophoblasts in 2D TSC culture, 18hrs in rotational

© 2025. Published by The Company of Biologists under the terms of the Creative Commons Attribution License (https://creativecommons.org/licenses/by/4.0/).

culture, and 48hrs in rotational culture and for our experiments using ezrin inhibitor treatment with the TSC model. Therefore, we prioritized validating our primary outcomes such as fusion, expression of ß-hCG and syndecan-1, microvillar formation, proliferation, viability, etc. through staining. Our representative images in Figures 4 and 7 are from 3-5 experimental replicates. Therefore, the sum of the data is from higher sample numbers (experimental replicates) than the RNA-sequencing. Our TSC experiments were conducted with a single TSC line due to the rate limiting availability of space in our bioreactor, meaning we could not conduct simultaneous experiments with multiple TSC lines. Future work using this model will include multiple lines with the acquisition of an additional bioreactor.

Major concerns:
1. Microvillar stabilization mechanisms. The manuscript demonstrates a clear role for microvilli in fusion but lacks mechanistic depth. Exploring signaling pathways or interactions beyond ezrin phosphorylation would add value.

We agree that a better understanding of the signalling pathways and mechanisms governing microvillar establishment and maintenance would provide additional insight into how microvillar stabilization supports trophoblast fusion. Unfortunately, aside from ezrin and actin, the molecular components of vCT microvilli are unknown. This precludes our ability to target microvillar stability in any other way without blocking whole cell actin dynamics. Our work attempting to destabilize ezrin via LOK inhibitor treatment that was included in the last revision highlights that trophoblast microvilli appear to be regulated in a cell-type specific manner. LOK is a highly conserved regulator of Ezrin Thr567 phosphorylation in other cell types (PMID: 19255442, 28430576), but this regulatory pathway is not conserved in trophoblasts. Without knowledge of the specific actin bundling proteins that are active within trophoblast microvilli, or the specific phospholipid profile of this cellular sub-compartment in vCT it is not possible to take a multipronged approach. We believe our data shows that future work on how vCT regulate the emergence and stability of microvilli should be a priority for our field.

2. Model limitations. The differences between the explant and organoid models are underexplored. Clarifying their biological relevance and potential clinical implications is necessary.

To address this comment, we have expanded on our discussion and included new analyses as requested by Reviewer 2 (Lines 215-230 and 386-408; Figures S3, S4, and S5). We emphasize that the explant model most accurately models ST regeneration while the organoid model models *de novo* ST development. We more explicitly contrast the two models in terms of cell composition, the presence or absence of extracellular matrix, and how this might affect our data analyzing polarization and ezrin inhibitor treatment.

3. Contradictory data. The apparent disconnect between increased fusogen expression and impaired fusion (Figures 6 and 7) suggests unexplored regulatory mechanisms. A more in-depth discussion or additional data are needed.

This is a valid comment and a fascinating direction of future study. These results suggest that while the machinery governing fusogen transcription is upregulated, that post-transcriptional factors lead to poor fusogen efficiency.

We believe that the downregulation of glycosylation conserved across explants and organoids with ezrin inhibition (Figures 6G, 7I) contributes to poor fusogen function. It has been established by Cui *et al.* (2016) (PMID: 26853155) that N-glycosylation is critical for mediating fusogenic function of syncytin-2. In the future, better understanding how ezrin inhibition leads to dysregulation of glycosylation in trophoblasts will be critical for understanding factors mediating fusion. This has been noted in lines 462-464 of the discussion.

Another hypothesis is that disrupting microvillar stabilization decreases the efficiency of fusogen engagement. Production of membrane protrusions on fusion-competent cells is very conserved across cell types and species (PMID: 30936162, 9695816) and seems to be necessary for increasing contact sites between apposing cells (PMID: 31527149), resulting in efficient fusogen engagement and fusion pore formation. This has been noted in lines 475-490 of the discussion. Unfortunately, there are currently no specific antibodies available that can detect syncytin-1 or syncytin-2,

© 2025. Published by The Company of Biologists under the terms of the Creative Commons Attribution License (https://creativecommons.org/licenses/by/4.0/).

therefore in this study we could not examine syncytin localization. In the future, we will develop CRISPR knock-in TSC to examine syncytin-1 and/or syncytin-2 localization with microvilli disruption.

4. Statistical robustness. Analyses with insufficient sample sizes compromise the reliability of conclusions. Either provide more replicates or justify the sample sizes used.

This comment has been addressed above in response to Reviewer 1, “responses to previous comments”, Point 5.

Minor comments:
1.Explanations for quantitative metrics. Figures 2D and 2F require clear definitions of parameters like shape factor and apical volume.

Thank you for this comment. We have included these definitions in the Methods section in Lines 610-615.

2. N-values in figures. Statistical figures should include n-values for transparency and reproducibility.

N-values for all statistical figures are included in the figure legends. For all bar graphs we have also included individual data points to represent n-values. For explants, all replicates represented are biological replicates and for organoids, all replicates represented are experimental replicates.

3. Figure clarity. Magnified insets for Figures 6D and 6F would help highlight critical observations.

Higher magnification insets have been added for Figures 6D and 6F.

4. Concentration of ezrin inhibitor. Justify the concentrations of NSC668394 used, supported by experimental data.

This is a valid point. We determined our concentrations of NSC668394 by quantifying the total amount of active phospho-Thr567-ezrin signal in explants pulsed for 2hrs with 0μM, 5μM, 10μM, 25μM, and 50μM of NSC668394. At 50μM NSC668394, we see the most statistically significant loss of phospho-Thr567-ezrin signal from the vCTs. This data has been added to Figure S6.

5. Language and grammar. Simplify and refine phrasing throughout the manuscript to improve readability.

The manuscript has been edited to improve readability.

Reviewer 2:
Advance Summary and Potential Significance to Field:
The authors’ revisions now provide a much more developed mechanistic insight into how ezrin-mediated actin protrusions are facilitating trophoblast cell-cell fusion. Using two excellent model systems, the authors show that ezrin is important for potentially creating a cellular structure that accumulates fusion proteins. This work is novel, builds on previous findings of ezrin, and advances the fields knowledge.

Reviewer 2 Comments for the Author:
The authors have made significant efforts to improve their manuscript.

In response to my previous comments, the authors attempted or addressed every single point. New experiments performed improve the overall quality of their work, and importantly, provide mechanistic insight into the role of actin-rich microvilli protrusions in trophoblast cell-cell fusion. In particular, their use of the CT29 TSC line cultured in a bioreactor to induce syncytialization in correct orientation is to be commended. Relatedly, work using bulk RNA-seq to examine gene pathways that are impacted in response to ezrin inhibition in the organoid system also add strength to the revised manuscript. Lastly, new work examining the localization of CD98 within actin-rich

© 2025. Published by The Company of Biologists under the terms of the Creative Commons Attribution License (https://creativecommons.org/licenses/by/4.0/).

structures provides new insight into how actin-rich protrusions are contributing to trophoblast syncytialization, and the role in which ezrin is facilitating this potential trafficking event.

As indicated, this revision is a marked improvement over the initial submission.

We would like to thank this reviewer for their positive assessment of this revised manuscript and their thoughtful suggestions throughout the process.

1. My only ask is that the authors perform a more thorough comparison between the explant and organoid bulk RNA-seq analyses. Because the effect of the ezrin inhibitor in the organoids impacted cell viability and proliferation, I feel a more careful examination in warranted. Integrating both RNA-seq objects and examining global differences/similarities could be helpful (PCs, PCC, or cluster analyses). I would expect there to be differences between the two systems, but visualizing these differences with a statistical inference will bolster the data as there seem to be important caveats related to the organoid data.

This is an excellent point, and we have included these new analyses in Figures S3, S4, S5 discussed in Lines 215-230 and 386-408. When comparing organoids at 18hrs and 48hrs in culture and explants at 24hrs and 72hrs in culture using PCA (Fig. S3A) and hierarchical clustering analyses (Fig. S3B), we found that samples clustered primarily by model type and secondarily by time in culture. To further elucidate the genes driving these global differences, we provided heatmaps and GO pathway terms comparing organoids at 18hrs with explants at 24hrs (Fig. S4) and comparing organoids at 48hrs with explants at 72hrs (Fig. S5). Interestingly for ST-related markers, organoids expressed relatively more *CGB, GCM1*, and *SDC1* while explants expressed more *PSG, PAPPA,* and *CSH2*. This suggests that different models may bias vCT differentiation into transcriptionally distinct ST or that ST gene expression varies depending on the culture method. GO pathway terms enriched in explants included extracellular matrix components and actin binding, likely contributed from the non-trophoblastic cell populations present. GO pathway terms enriched in organoids included helicase activity and histone kinase activity potentially due to the relatively higher proportion of proliferative cells in this model. Thus, there are significant transcriptional differences between the two models and in the future these differences will be critical when selecting models or interpreting results. We intend to use single cell RNA-seq and deconvolution methods to directly address the differential gene expression in trophoblastic populations between the models in the future when funding permits.

Reviewer 4:

Reviewer 4 Comments for the Author:

The study by Duan et al. addresses the mechanisms responsible for fusion of villous cytotrophoblast into the overlying syncytiotrophoblast layer in the human placenta. This is a topic of fundamental translational and clinical importance, for maintaining the integrity of the syncytiotrophoblast layer is critical for successful pregnancy, as evidenced by alterations of this layer in several adverse pregnancy outcomes. Overall, the author responses to the reviewer comments were both very thorough and thoughtful. Addition of the trophoblast organoid model to demonstrate the conserved nature of ezrin inhibitor treatment on the fusion process in two very different model systems strengthens the manuscript. In addition, the authors performed a number of important control experiments to more clearly delineate the effects of ezrin inhibitor treatment on trophoblast, including measures of cell proliferation and viability. While ezrin inhibitor treatment blocked trophoblast proliferation in the trophoblast organoid model but not in explants, this is not really surprising, as it is well established that little proliferation occurs in placental explants cultured under ex vivo conditions (with the exception of columnar cytotrophoblast cultured on Matrigel). Thus, suppression of VCT fusion by ezrin inhibitor appears to occur independently of effects of proliferation in the placental explant model system. Similarly, inclusion of the RNAseq data for both the explants and organoids markedly strengthened the manuscript by not only providing a more comprehensive assessment of the effects of ezrin inhibitor treatment on gene expression, but also demonstrating that ezrin inhibition alters pathways involved in control of protein glycosylation and processing. Lastly, the authors provided new experiments to implicate the transmembrane glycoprotein CD98 in trophoblast fusion.

© 2025. Published by The Company of Biologists under the terms of the Creative Commons Attribution License (https://creativecommons.org/licenses/by/4.0/).

While reviewer 3 is absolutely correct that ezrin was previously shown by Pidoux et al. to play an essential role in villous cytotrophoblast fusion into syncytiotrophoblast, there are some fundamental differences between the focuses and measured endpoints of the previous study and the current manuscript. Pidoux et al. focused primarily on identification of the key cellular signaling components (i.e., PKA-ezrin-Cx43) involved in trophoblast fusion using isolated, term villous cytotrophoblast as a model. In contrast, the current study now includes utilization of two different model systems to provide new information on the role of cytotrophoblast microvilli in fusion. Importantly, the revised manuscript also provides new information on the downstream consequences of blocking ezrin activity in trophoblast.

We would like to thank this reviewer for their kind comments and clear enthusiasm for the revised manuscript and throughout the review process. We agree that this manuscript was much improved with revisions and appreciate this reviewer's positive assessment of our work.

Third decision letter

MS ID#: dev.204619R2

MS TITLE: Placental cytotrophoblast microvillar stabilization is required for cell-cell fusion

AUTHORS: Wendy K. Duan; Sumaiyah Z. Shaha; Juan F. Garcia Rivas; Bethan L. Wilson; Khushali J. Patel; Ivan K. Domingo; Meghan Riddell
ARTICLE TYPE: Research Article

I am happy to tell you that your manuscript has been accepted for publication in Development, pending our standard publication integrity checks.

Reviewer 1: No comments provided

Reviewer 2: SUMMARY OF THE ADVANCE MADE IN THIS PAPER AND ITS POTENTIAL SIGNIFICANCE TO THE FIELD

As stated in my previous review, the manuscript by Duan et al examines in more detail the role of ezrin in trophoblast cell fusion. I feel the findings advance the field's knowledge and provide new areas of investigation related to the role of microvillous protrusions in trophoblast fusion. The author's revision (round 2) significantly improved the mechanistic of the role of ezrin in promoting trophoblast fusion, and my only suggestion was for a more indepth comparison between explant and organoid bulk-seq data. The authors performed a sufficient characterization of these datasets and show data that are informative of differences between the two systems that are important for when comparing the two approaches to model cell fusion. This additional work further strengthens the manuscript.

SUGGESTIONS TO AUTHORS
I have no additional requests. There are interesting questions related to what these microvillous protrusions are doing during cell fusion, but I feel additional experimentation focussed on this biology falls outside the scope of this majorly revised and improved manuscript.

Reviewer 3: The authors have again responded to the reviewer comments in a comprehensive manner, collectively alleviating concerns and/or providing reasonable rationales for not providing additional data at this time. The collective modifications that the authors made in response to the critiques have significantly strengthened the manuscript and have resulted in a novel contribution to our understanding of the biology underlying trophoblast fusion. One significant remaining concern is that the authors felt constrained by space limitations to remove the previous discussions of: 1) the potential impact of the changing placental microenvironment (i.e., blood flow, oxygen levels etc.) during gestation on VCT fusion and 2) potential differences in the precise molecular

© 2025. Published by The Company of Biologists under the terms of the Creative Commons Attribution License (https://creativecommons.org/licenses/by/4.0/).

mechanisms driving fusion over the course of gestation. Since it is well-established that defects and/or alterations in the syncytiotrophoblast layer are associated with adverse pregnancy outcomes such as preeclampsia and FGR, it would seem that allowance for a slightly larger manuscript to include these discussion points would be valuable to the field.

© 2025. Published by The Company of Biologists under the terms of the Creative Commons Attribution License (https://creativecommons.org/licenses/by/4.0/).
